# Anionic Templates Drive Conversion between a Zn^II^_9_L_6_ Tricapped Trigonal Prism and Zn^II^_6_L_4_ Pseudo-Octahedra

**DOI:** 10.1021/jacs.3c03981

**Published:** 2023-07-13

**Authors:** Hua-Kui Liu, Tanya K. Ronson, Kai Wu, Dong Luo, Jonathan R. Nitschke

**Affiliations:** Yusuf Hamied Department of Chemistry, University of Cambridge, Cambridge CB2 1EW, U.K.

## Abstract

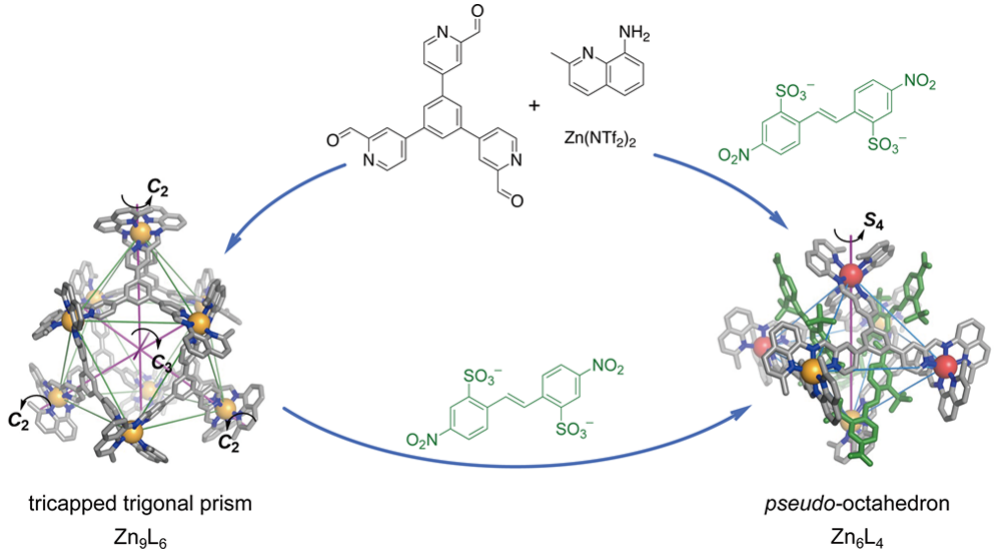

This work introduces the use of 8-aminoquinoline subcomponents to generate complex three-dimensional structures. Together with a tris(formylpyridine), 8-aminoquinoline condensed around Zn^II^ templates to produce a tris(tridentate) ligand. This ligand is incorporated into either a tricapped trigonal prismatic Zn^II^_9_L_6_ structure or a pair of pseudo-octahedral Zn^II^_6_L_4_ diastereomers, with *S*_4_ and *D*_2_ symmetries. Introduction of a methyl group onto the aminoquinoline modulated the coordination sphere of Zn^II^, which favored the Zn^II^_9_L_6_ structure and disfavored the Zn^II^_6_L_4_ assembly. The tricapped trigonal prismatic Zn^II^_9_L_6_ architecture converted into a single Zn^II^_6_L_4_ cage diastereomer following the addition of a dianionic 4,4′-dinitrostilbene-2,2′-disulfonate guest. Four of these guests clustered tightly at the four windows of the Zn^II^_6_L_4_ cage, held in place through electrostatic interactions and hydrogen bonding, stabilize a single diastereomeric configuration with *S*_4_ symmetry.

## Introduction

Three-dimensional coordination cages with well-defined enclosed cavities have found applications in stabilizing reactive species,^1,2^ binding and sensing guests,^3^ chemical separations,^4^ and catalyzing reactions.^5^ The subcomponent self–assembly strategy has enabled the preparation of many metal–organic capsules from simple building blocks, as intricate products form from amine and aldehyde precursors together with metal ions via the concurrent formation of dynamic coordinative and reversible covalent imine bonds.^6^

Cages such as tetrahedra,^7^ cubes,^8^ and octahedra^9−11^ have thus been prepared. These high-symmetry cages contain pseudo-spherical cavities,^12^ which enable them to bind approximately spherical guests. Metal–organic cages with lower symmetry^13^ could enable the binding of non-spherical guests, such as biomolecules and pharmaceuticals. For example, a triangular prismatic architecture with an anisotropic cavity is capable of binding a series of asymmetric drugs and natural products.^14^ Clever and co-workers used a low-symmetry bowl-shaped metal–organic cage^15^ to act as a supramolecular mask of bound C_60_, to effect mono-functionalization of the fullerene on its unprotected face, rather than the bi-functionalization that occurs within a cubic coordination cage or a metal–organic framework.^16^ New methods of preparing low-symmetry cages are thus very much worth pursuing.

**Figure 1:**
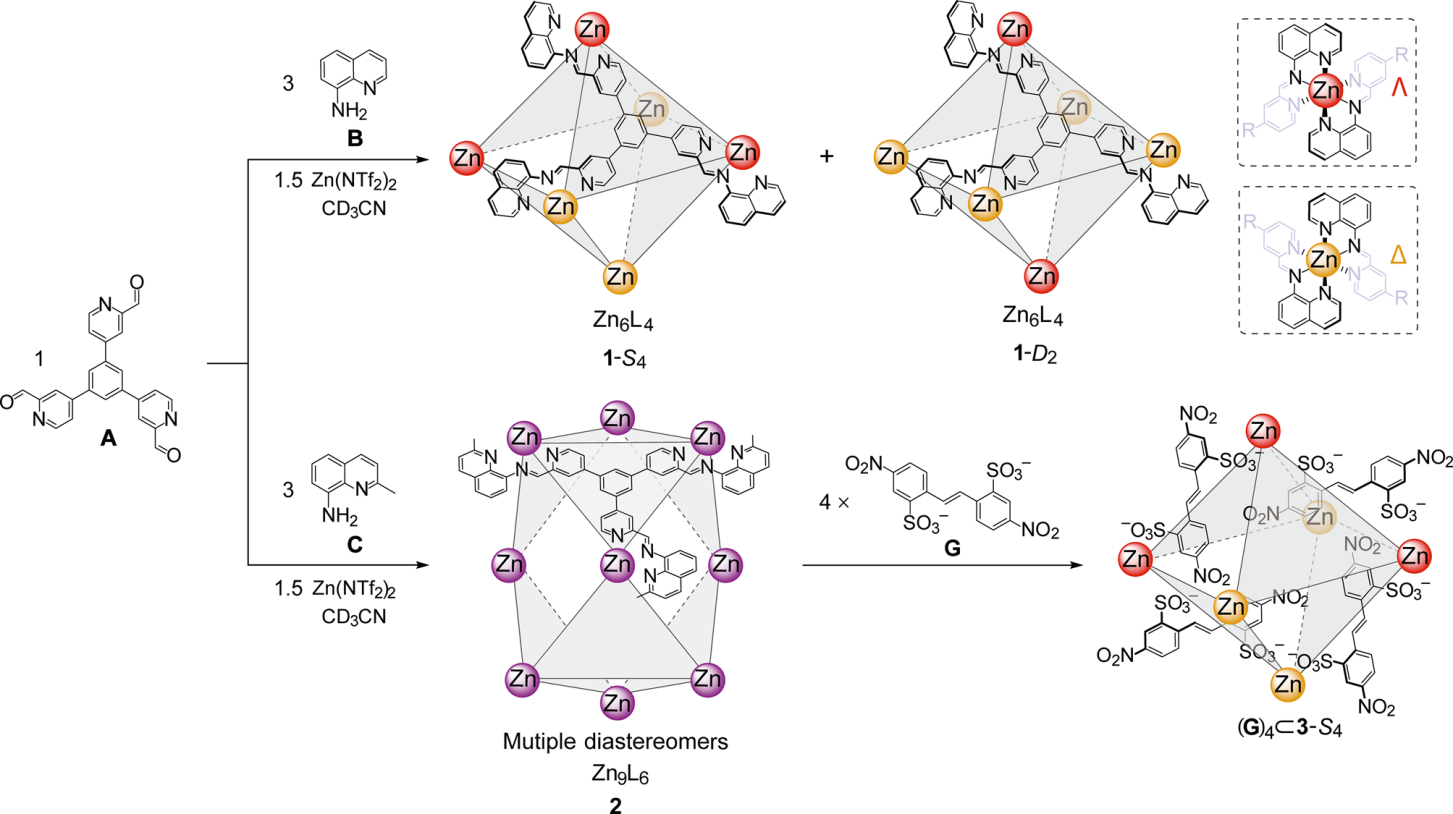
Subcomponent self–assembly of Zn^II^_6_L_4_**1** and Zn^II^_9_L_6_**2** and the guest-templated conversion of **2** to (**G**)_4_⊂**3**-*S*_4_. Δ and Λ metal stereoconfigurations are shown in yellow and red, respectively.

Methods that have been developed to construct low-symmetry cages^13^ include the use of low-symmetry or flexible ligands,^17^ solvent effects,^18^ the use of anions and other templates,^9f,19^ heteroleptic architectures,^14^ and multimetallic assemblies.^20^ However, creating low-symmetry cages by engineering the stereochemistry of metal vertices has proven challenging.^21^ M_6_L_4_ architectures with different *C*_2_-symmetric vertices (Table S1) usually display high symmetry, with rare exceptions.^22^ We hypothesized that the coordination-vector geometry of tris(tritopic) ligands shown in Figure 1 would lead to the generation of a new class of M_6_L_4_ cages, where steric hindrance within a ligand (Table S2) might lead to the formation of a lower-symmetry cage, and clashes between ligands may favor the formation of higher-nuclearity structures. The formation of structures **1** and **2** (Figure 1) supported these hypotheses, as detailed below.

Here, we report the preparation of two novel types of low-symmetry metal–organic cage structures—a Zn^II^_9_L_6_ tricapped trigonal prism, and two Zn^II^_6_L_4_ architectures with octahedral metal-ion frameworks, but *S*_4_ and *D*_2_ point symmetries, assembled from the same tris(formylpyridine) subcomponent **A** (Figure 1) under different reaction conditions. The Zn^II^_9_L_6_ architecture, which is not among the more commonly observed Archimedean and Platonic solids, converts into a *S*_4_-symmetric Zn^II^_6_L_4_ cage through the action of disulfonate templates (Figure 1).

## Results and Discussion

### Self–Assembly of Zn^II^_9_L_6_ and Zn^II^_6_L_4_ Metal–Organic Cages

Subcomponent **A** was prepared through Suzuki–Miyaura cross-coupling of 1,3,5-tris(4,4,5,5-tetramethyl-1,3,2-dioxaborolan-2-yl) benzene and 4-bromopicolinaldehyde (see Supporting Information Section 2). The reaction of **A** (4 equiv) and 8-aminoquinoline **B** (12 equiv) with zinc(II) bis(trifluoromethanesulfonyl)imide (triflimide or NTf_2_^–^, 6 equiv) in CD_3_CN at 70 °C resulted in the formation of Zn^II^_6_L_4_ cage **1**. HR–ESI–MS showed a sharp set of peaks, corresponding to charge states from +8 to +4, all of which confirmed a Zn^II^_6_L_4_ composition (Figures S15 and S16).

**Figure 2:**
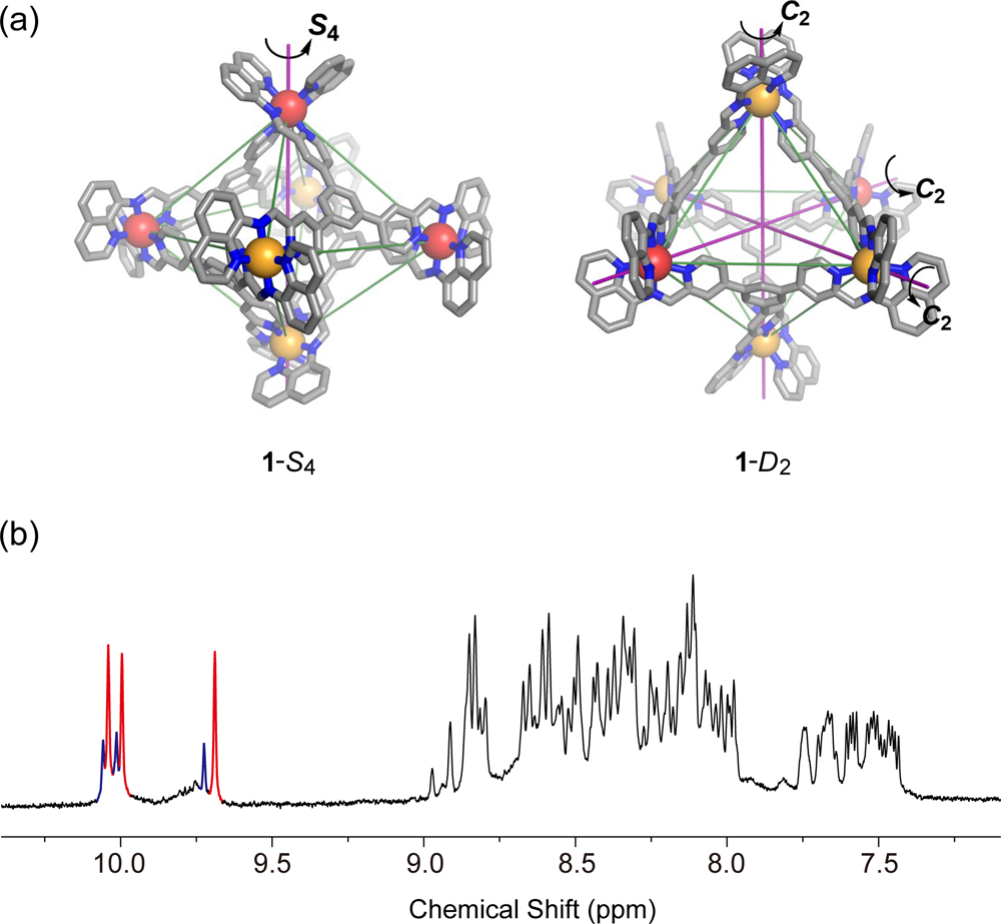
(a) PM7-optimized structure of the *S*_4_ and *D*_2_ diastereomers of cage **1**. Color scheme: Δ-Zn, yellow; Λ-Zn red. (b) ^1^H NMR spectrum (400 MHz, 298 K, CD_3_CN) of cage **1**. The two sets of imine peaks with a 1:1:1 integration ratio are highlighted.

The ^1^H NMR spectrum of **1** contained two sets of ligand signals in a 2:1 ratio (Figure 2b). Three magnetically distinct chemical environments for the ligand protons of **1** were observed, in a 1:1:1 integrated ratio. The ^1^H NMR diffusion-ordered spectrum (DOSY) of **1** showed that all of its signals had the same diffusion coefficient of 4.05 × 10^–6^ cm^2^ s^–1^ (Figure S10), consistent with the formation of multiple diastereomeric species with a common size of 3.0 nm modeled using the PM7^23^ force field of Scigress^24^ (Figure S64). All of the protons of **1** were assigned using different two-dimensional NMR techniques (Figures S8, S11, and S12).

We sought to elucidate the configurations adopted by cage **1** through NMR analysis. The ligands adopt configurations with different torsions between the central phenyl and peripheral pyridine rings to minimise ring eclipsing, similar to, but less regular than, the propeller-like configurations observed in higher-symmetry structures.^25^ Geometrical analysis of cage **1** reveals six enantiomeric pairs of diastereomers, with distinct point symmetries: Δ_6_/Λ_6_, *T*; Δ_5_Λ/ΔΛ_5_, *C*_2_; Δ_4_Λ_2_/Δ_2_Λ_4_, *C*_1_; Δ_4_Λ_2_/Δ_2_Λ_4_, *D*_2_; Δ_3_Λ_3_, *C*_3_; and Δ_3_Λ_3_, *S*_4_. These stereoisomers are shown in Figure S64.

M_6_L_4_ cages with *T* point symmetry^11^ afford ^1^H NMR spectra with only one set of ligand peaks, whereas such cages with reduced symmetry, *e.g.*, with *C*_2_, *C*_1_, *C*_3_, *S*_4_, or *D*_2_ point symmetry, are predicted to give rise to spectra with 6, 12, 4, 3, or 3 imine singlet peaks, respectively. Because the ^1^H NMR spectrum of **1** exhibited two sets of three 1:1:1 imine singlet peaks (Figure 2b), we inferred that **1** existed in solution as a mixture of diastereomers with *S*_4_ and *D*_2_ symmetries.

To confirm this conclusion, energy minimization of the six stereoisomers was carried out at the PM7^23^ level of theory (Figure S64 and Tables S8–S13) using Scigress.^24^ Although the calculated energies of these isomers were similar, the **1**-*S*_4_ and **1**-*D*_2_ diastereomers adopted larger dihedral angles between the ligand phenylene and pyridyl rings, which are closer to the values adopted by the free ligand, than for the other four isomers (Table S6). We infer that the larger dihedral angles may favor the **1**-*S*_4_ and **1**-*D*_2_ diastereomers, because in these diastereomers, the steric eclipsing of phenylene and pyridyl hydrogen atoms on the same ligand is reduced. These dihedral angles in the PM7-optimized model of **1**-*S*_4_ are similar to those observed in the crystal structure of **3**-*S*_4_ (Table S6, entries 6–7), and the Zn···Zn separations are similar between model and structure, further suggesting that the *S*_4_ diastereomer of **1**, and its *D*_2_-symmetry analog, with larger dihedral angles according to the model, are energetically favored. We thus conclude that torsional steric hindrance of our new ligand, combined with the coordinate geometry of its vertices, led to the formation of M_6_L_4_ cages with *D*_2_ and *S*_4_ point symmetries, rather than the higher-symmetry pseudo-octahedra (Table S2).

As concentration dictates the formation of cages with different nuclearities according to Le Chatelier’s principle, we hypothesized that increasing the concentration might favor the conversion of a smaller cage into a larger one. Indeed, when the total concentration of **A** was increased from 0.5 to 2.0 mM during the synthesis of **1**, a new species with a Zn^II^_9_L_6_ composition was detected by HR–ESI–MS alongside Zn^II^_6_L_4_**1**. To promote the selective formation of Zn^II^_9_L_6_**2**, we sought to modulate the coordination sphere of Zn^II^ through methylation α to the quinoline nitrogen atom of subcomponent **B**. The analysis of models using the PM7^23^ force field of Scigress^24^ (Table S7) suggests that the introduction of such a methyl group would favor the formation of Zn^II^_9_L_6_ cage **2** over Zn^II^_6_L_4_ cage **1**. Thus, the assembly of subcomponent 2-methyl-8-aminoquinoline **C** (18 equiv) together with **A** (6 equiv) and Zn^II^(NTf_2_)_2_ (9 equiv) at [**A**] = 5.0 mM resulted in the formation of Zn^II^_9_L_6_**2** as the uniquely observed product.

The ^1^H NMR spectrum of **2** (Figure S17) was complex, with many signals, which we ascribe to the presence of multiple diastereoisomers. The ^1^H DOSY spectrum of **2** (Figure S19) indicated that all signals assigned to cage diastereomers had the same diffusion coefficient, consistent with the formation of isomers with similar sizes. The complex ^1^H NMR spectrum was assigned using different two-dimensional NMR techniques (Figures S20–S23). The construction of Zn^II^_9_L_6_ cage **2** is thus enabled through a detailed understanding of the subtle steric effects of the aminoquinoline methyl groups and the phenylene-pyridine torsion angles (Table S6).

### Anionic Templates Drive Conversion of Zn^II^_9_L_6_ to Zn^II^_6_L_4_

The Zn^II^_6_L_4_ and Zn^II^_9_L_6_ frameworks of coordination cages **1** and **2** have distinct geometries, which imply different guest-binding preferences. We hypothesized that the conversion between these frameworks might be achieved following the addition of a suitable guest. Therefore, the use of anionic guests as templates (Figure S26) was investigated to effect the guest-induced conversion from Zn^II^_9_L_6_**2** to Zn^II^_6_L_4_**3**, an analogue of cage **1** that incorporated methylated **C** instead of **B**.

**Figure 3:**
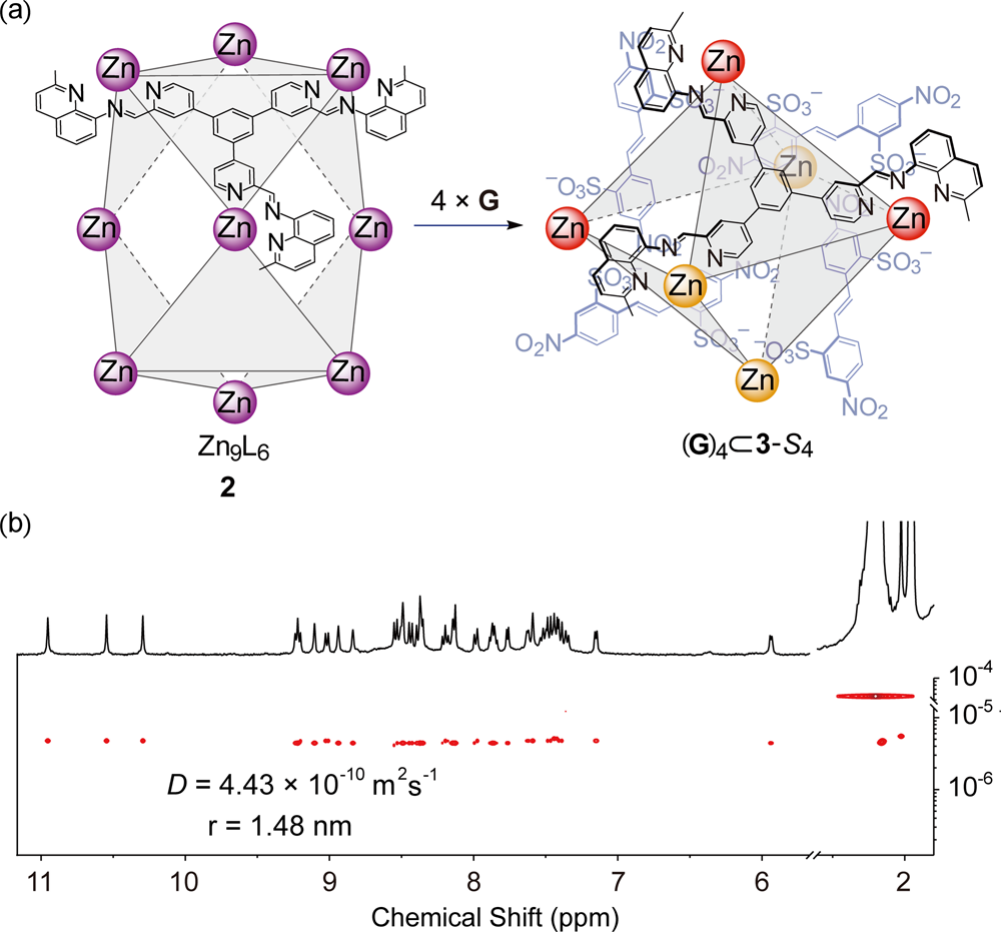
(a) Guest-templated transformation of **2** into (**G**)_4_⊂**3**-*S*_4_. (b) ^1^H DOSY NMR spectrum (400 MHz, 298 K, CD_3_CN) of (**G**)_4_⊂**3**-*S*_4_. **G** = 4,4′-dinitrostilbene-2,2′-disulfonate.

Complete conversion of Zn^II^_9_L_6_**2** to the octahedral Zn^II^_6_L_4_ framework of **3** occurred after the addition of anionic metal cluster [PO_4_(WO_3_)_12_]^3–^ in CD_3_CN. The NMR spectra of **3** were complex (Figure S27), suggesting the presence of multiple diastereomers. However, HR–ESI–MS showed only peaks corresponding to the [PO_4_(WO_3_)_12_]^3–^ adduct of **3** (Figures S28 and S29). The addition of **G** (**G** = 4,4′-dinitrostilbene-2,2′disulfonate) to Zn^II^_9_L_6_**2** in acetonitrile, in contrast, led to complete conversion of **2** to (**G**)_4_⊂**3** after 6 h,^26^ as confirmed by HR–ESI–MS (Figures S38 and S39). The conversion of **2** to (**G**)_4_⊂**3** in dilute solution was accelerated due to the poor solubility of **G** and (**G**)_4_⊂**3** (Figures S40–S42). NMR spectra (Figures 3b and S31–S37) indicated the formation of a single isomer with either *S*_4_ or *D*_2_ symmetry, as reflected in the presence of only three imine peaks in a 1:1:1 integral ratio. DOSY confirmed that all the ligand and guest peaks exhibited a single diffusion rate (Figure 3b).

**Figure 4:**
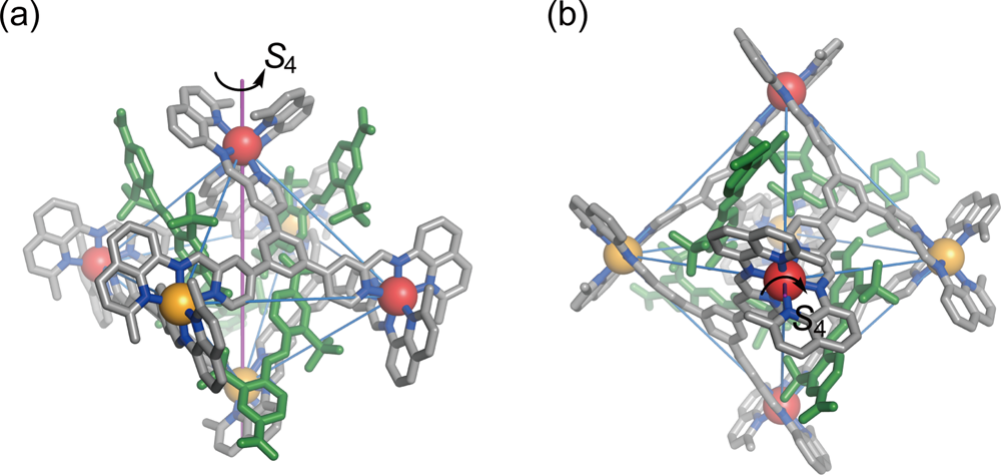
X-ray crystal structure of host–guest complex (**G**)_4_⊂**3**-*S*_4_. (a) View orthogonal to the *S*_4_ axis, showing the compression of the octahedral framework along this axis. (b) View down the *S*_4_ axis, showing the equatorial expansion of the framework. Color scheme: Δ-Zn, yellow; Λ-Zn, red; guest, green; *S*_4_ axis, purple. Ligand nitrogen atoms are blue, and the carbon atoms are gray.

Vapor diffusion of diethyl ether into an acetonitrile solution of (**G**)_4_⊂**3** containing KSbF_6_ resulted in the formation of cube-shaped yellow crystals.^27^ Single-crystal X-ray diffraction analysis revealed the solid state structure of (**G**)_4_⊂**3** (Figure 4), having an *S*_4_ symmetry consistent with the NMR spectrum recorded in solution (Figure 3b). Inspection of the structure revealed three of the Zn^II^ centers to have Δ handedness, with the other three adopting Λ handedness, lending the capsule achiral *S*_4_ point symmetry. Each metal center is thus related by the *S*_4_ symmetry operation (Figure 4) to a metal center of the opposite stereochemical configuration.

We infer the structure of (**G**)_4_⊂**3** to be stabilized by electrostatic attraction and hydrogen bonding (Figure S43) between the cationic cage framework and the anionic guests. Each guest occupies an open face of the octahedral host framework, with each sulfonate group oriented toward a Zn^II^ center. This arrangement thus appears to stabilize the octahedral Zn^II^_6_L_4_**3** framework with respect to the larger tricapped trigonal prismatic Zn^II^_9_L_6_**2**.

Structures having an octahedral metal framework but lower symmetry are rare;^22^ other M_6_L_4_ octahedra are observed to adopt *O*_*h*_,^9^*T*_*d*_,^10^ or *T*(11) symmetry. The complex (**G**)_4_⊂**3**-*S*_4_ displayed antipodal metal···metal distances of 20.6 and 16.1 Å, with the Zn^II^ centers on the *S*_4_ axis more closely spaced. We infer this axial compression and equatorial expansion to result from the reduced steric eclipsing of the phenylene and pyridyl hydrogen atoms within ligands, as compared to diastereomers with *C*_1_, *C*_2_, *C*_3_, and *T* configurations, as supported by PM7 calculations^23^ (Table S6). The dihedral angles between the chelate planes of two imino-quinoline moieties coordinated to each Zn^II^ center range from 84–88°.

### Host–Guest Chemistry of Zn^II^_6_L_4_

The complex NMR spectra of Zn^II^_9_L_6_ cage **2** hampered studies of its host–guest chemistry. We thus focused upon the host–guest chemistry of cage **1**. To determine the binding stoichiometry and affinities for different guests, we performed titration experiments by ^1^H NMR spectroscopy. Job plots^28^ of the titration of B(*p*-C_6_H_4_Cl)_4_^–^ into a solution of **1** were consistent with a 1:4 host–guest binding ratio (Table 1, and Figures S44 and S45). Given the size of the guest, we infer the guests to bind peripherally, at the four cage windows, in contrast to previously reported 1:1 peripheral binding of this guest to a different M_6_L_4_ cage with *T* symmetry.^11c^

**Table 1:** Summary of the Binding Constants of Anionic Guests to Cage **1**

guest	volume (Å^3^)^a^	*K*_a_^b^	inferred H/G stoichiometry
B(*p*-C_6_H_4_Cl)_4_^–^	386.8	(2.16 ± 0.05) × 10^2^	1:4
B(*p*-C_6_H_4_F)_4_^–^	339.6	(1.36 ± 0.04) × 10^2^	1:4
IO_4_^–^	64.2	(5.73 ± 0.32) × 10^2^	1:2
ReO_4_^–^	77.3	(5.98 ± 0.33) × 10^2^	1:2
[PO_4_(WO_3_)_12_]^3–^	692.4	_	1:1^c^

a Van der Waals volumes of anions based on the crystal structures (Table S5) were calculated by MoloVol.^31^

b *K*_a_ (M^–1^) were determined by ^1^H NMR spectroscopy.

c Bound by Zn^II^_6_L_4_ cage **3**.

The NMR titration data of guests B(*p*-C_6_H_4_Cl)_4_^–^ and B(*p*-C_6_H_4_F)_4_^–^ with **1** were plotted and fitted to the Hill function.^29^ These anions displayed Hill coefficients of ca. 1.3 and 1.4, respectively, indicating a weakly cooperative binding mode (Figures S50–S53). The association constants for the B(*p*-C_6_H_4_Cl)_4_^–^ and B(*p*-C_6_H_4_F)_4_^–^ were determined to be 2.16 × 10^2^ and 1.36 × 10^2^ M^–1^, respectively.

In contrast to the anionic guests B(C_6_H_5_)_4_^–^, B(*p*-C_6_H_4_Cl)_4_^–^, and B(*p*-C_6_H_4_F)_4_^–^, the more electron-deficient pentafluorophenyl and tetrakis[3,5-bis(trifluoromethyl)phenyl] borates were not bound by **1** (Figures S50–S53, S59, and S63). We infer that the increased electron-deficiency of the polyfluorinated tetraphenylborates could prevent binding due to the weaker electrostatic interaction between these anionic guests and cationic host **1**.

The oxoanions IO_4_^–^ and ReO_4_^–^ bound with a 1:2 host–guest stoichiometry, with similar association constants of 5.73 × 10^2^ and 5.98 × 10^2^ M^–1^, respectively (Figures S46–S49 and S54–S57). Noting that four equivalents of the peripherally binding larger anions B(*p*-C_6_H_4_Cl)_4_^–^ and B(*p*-C_6_H_4_F)_4_^–^ bound to **1**, we infer that the smaller ones ReO_4_^–^ and IO_4_^–^ bound internally. Two equivalents of these oxoanions fit easily within the cavity of **1** (Tables 1, S4, and S5),^30,31^ and internal binding of similar anions was observed in related M_6_L_4_ species.^11c^ As the volume of [PO_4_(WO_3_)_12_]^3–^ exceeds the cavity volume of **3** (Tables S4 and S5), we infer that this cluster is bound peripherally.

To further study the binding ability of **1** toward neutral guests, we treated the cage with polyaromatic hydrocarbons such as pyrene, corannulene, triphenylene, and dibenzo[g, p]chrysene, all of which were observed to bind to cage **1** in fast exchange on the NMR timescale (Figures S58 and S60–S62). However, the poor solubility of these guests precluded *K*_a_ determination.

## Conclusions

Novel Zn^II^_9_L_6_ tricapped trigonal prism and Zn^II^_6_L_4_ structures with *S*_4_ and *D*_2_ symmetries thus establish the use of 8-aminoquinolines in subcomponent self–assembly of complex three-dimensional structures, beyond their use in copper(I) helicates.^32^ These zinc(II) architectures bind a diverse array of guests, and show the ability to reconfigure to optimize guest binding. Such dynamic reconfiguration might enable the preparation of new classes of heteroleptic structures with lower symmetries,^33^ capable of binding low-symmetry guests. Such species are potentially of interest in the purification of low-symmetry, complex molecules from mixtures.^4b^

## Supporting Information Available

Experimental procedures; NMR characterizations; mass spectrometry data; volume calculations; and X-ray crystallographic data (PDF)
