# Supplementary material for: Anionic Templates Drive Conversion between a ZnII9L6 Tricapped Trigonal Prism and ZnII6L4 Pseudo-Octahedra
Source: J Am Chem Soc. 2023 Jul 13;145(29):15990–6. doi: 10.1021/jacs.3c03981 (PMC10375523; doi:10.1021/jacs.3c03981)
Supplement: Supplementary file 1 — ja3c03981_si_001.pdf [file ja3c03981_si_001.pdf]

Anionic Templates Drive Conversion Between a $Zn^{II}_9L_6$ Tricapped Trigonal Prism and $Zn^{II}_6L_4$ *Pseudo*-Octahedra

Hua-Kui Liu, Tanya K. Ronson, Kai Wu, Dong Luo, Jonathan R. Nitschke*

Yusuf Hamied Department of Chemistry, University of Cambridge,

Cambridge CB2 1EW, UK.

jrn34@cam.ac.uk

Supporting Information

Table of Contents

1 General Information S3

2 Synthesis and Characterization of **A** S4

3 Self-Assembly and Characterization of Cages **1** and **2** S8

3.1 Self-Assembly and Characterization of Cage **1** S8

3.2 Self-Assembly and Characterization of Cage **2** S15

4 Conversion of $Zn^{II}_9L_6$ to $Zn^{II}_6L_4$ S24

4.1 Templates Exploration to Convert $Zn^{II}_9L_6$ into $Zn^{II}_6L_4$ S24

4.2 Trianionic Dodecatungstophosphate as a Template S25

4.3 Dianionic 4,4'-Dinitrostilbene-2,2'-disulfonate as a Template S28

5 NMR Titrations of Guests with Cage **1** S39

5.1 Job Plot of NMR Titrations S39

5.2 Hill Equation Analyses of NMR Titrations S42

6 Host-guest Studies Using Cage **1** S48

6.1 Binding Guests for Cage **1** S48

6.2 Non-binding Guests for Cage **1** S50

7 Contrast of Octahedra with Low or High Symmetry S51

8 X-ray Crystallography S53

9 Volume Calculations S56

10 Optimized Structures of $Zn^{II}_6L_4$ and $Zn^{II}_9L_6$ Cages S57

11 References........ S89

1 General Information

Unless otherwise specified, all reagents were purchased from commercial sources and used as received. **A** was prepared from commercially available 1,3,5-tris(4,4,5,5-tetramethyl-1,3,2-dioxaborolan-2-yl) benzene and 4-bromopicolinaldehyde. Self-assembly reactions were performed in either deuterated or distilled CH_3CN.

NMR spectra were recorded using the following NMR spectrometers: Bruker 400 MHz Avance III HD smart probe (^{1}H, and ^{1}H–DOSY), Bruker Avance 500 MHz DCH cryoprobe (^{1}H, ^{13}C, and 2D NMR) or Bruker Avance 500 MHz TCI cryoprobe (^{1}H NMR). Chemical shifts of the NMR spectra are reported relative to the residual $CHCl_3$ in $CDCl_3$ (^{1}H NMR: δ = 7.26 ppm, ^{13}C NMR: δ = 77.0 ppm), or CHD_2CN (^{1}H NMR: δ = 1.94 ppm, ^{13}C NMR: δ = 118.3 ppm). Data for ^{1}H NMR spectra were reported as follows: chemical shift (ppm), peak shape (s = singlet, d = doublet, m = multiplet), coupling constant (Hz), and integration. Data for ^{1}H NMR and ^{13}C NMR are reported in terms of chemical shift (ppm).

High resolution electrospray ionization mass spectra (HR–ESI–MS) were recorded on a Waters Synapt G2-Si instrument.

2 Synthesis and Characterization of A

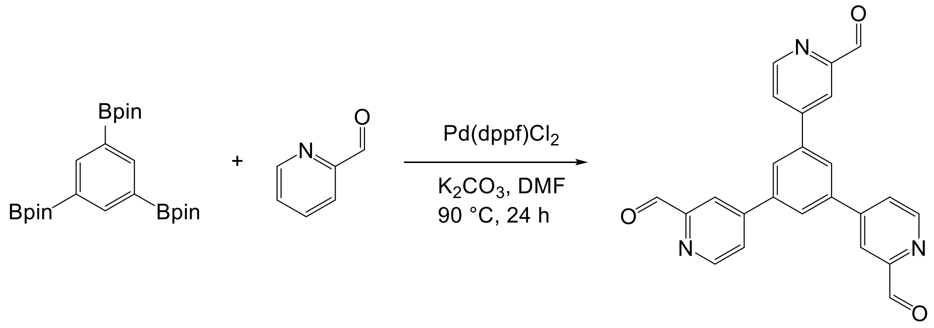

A suspension of 1,3,5-tris(4,4,5,5-tetramethyl-1,3,2-dioxaborolan-2-yl)benzene (0.47 g, 1.03 mmol, 1.0 equiv), 4-bromopicolinaldehyde (0.97 g, 5.2 mmol, 5 equiv), K_2CO_3 (1.70 g, 16 mmol, 15.5 equiv) and Pd(dppf)Cl_2 (0.25 g, 0.34 mmol, 0.34 equiv) in dimethylformamide (DMF, 60 mL) was degassed under a stream of nitrogen for 20 min.[1] The reaction mixture was heated at 90 °C for 24 hours under nitrogen. After cooling to room temperature, DMF was evaporated under reduced pressure. Brine (50 mL) was then added, and the suspension was extracted with CH_2Cl_2 (3 × 50 mL). The combined organic phases were dried over anhydrous Na_2SO_4, filtered, and the solvents evaporated under reduced pressure. Purification by flash column chromatography on silica gel using CH_2Cl_2/MeOH = 40/1 – 20/1 as eluent afforded **A** as a brown solid (0.25 g, 62%). R_f = 0.3 (MeOH/CH_2Cl_2 = 20/1).

^{1}H NMR (500 MHz, $CDCl_3$): δ 10.20 (s, 9H), 8.95 (d, J = 5.5 Hz, 1H), 8.29 (s, 1H), 8.05 (s, 1H), 7.87 (d, J = 5.5 Hz, 1H).

^{13}C NMR (125 MHz, $CDCl_3$): δ 193.2, 153.7, 151.1, 148.2, 139.8, 126.9, 125.7, 119.5.

HR–ESI–MS: m/z = 394.1187, $[M+H]^+$, (calc. 394.1186).

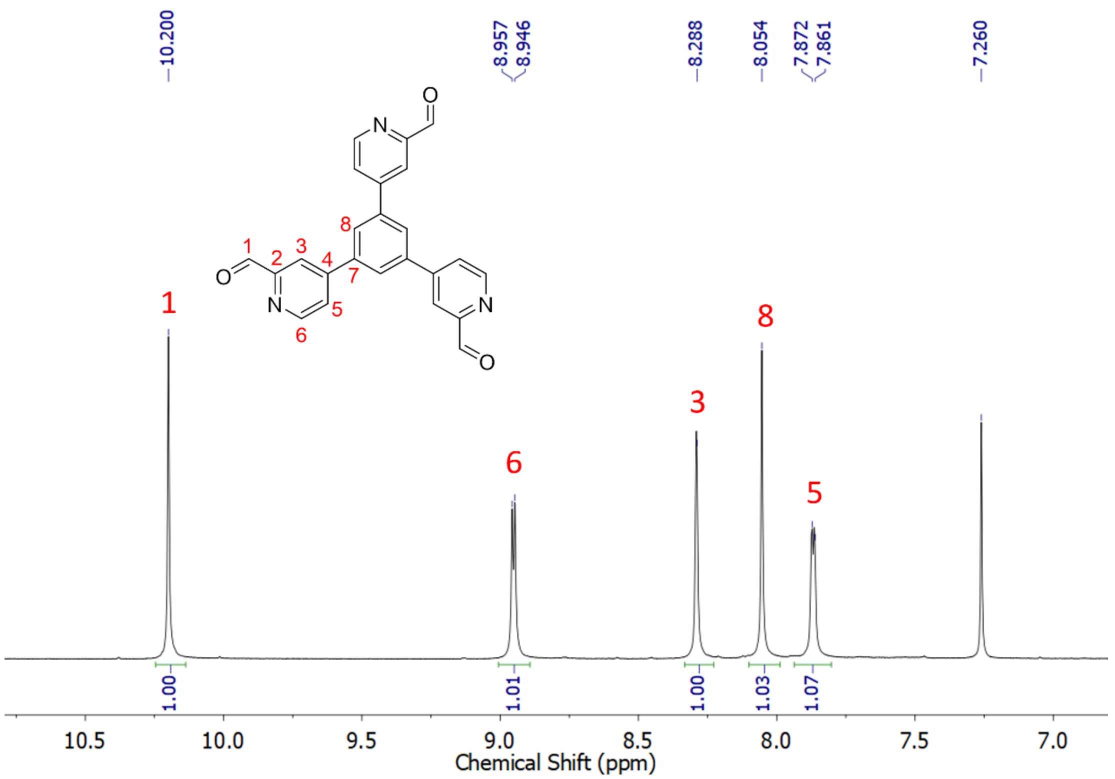

Figure S1. ^{1}H NMR spectrum of **A** (500 MHz, $CDCl_3$, 298 K).

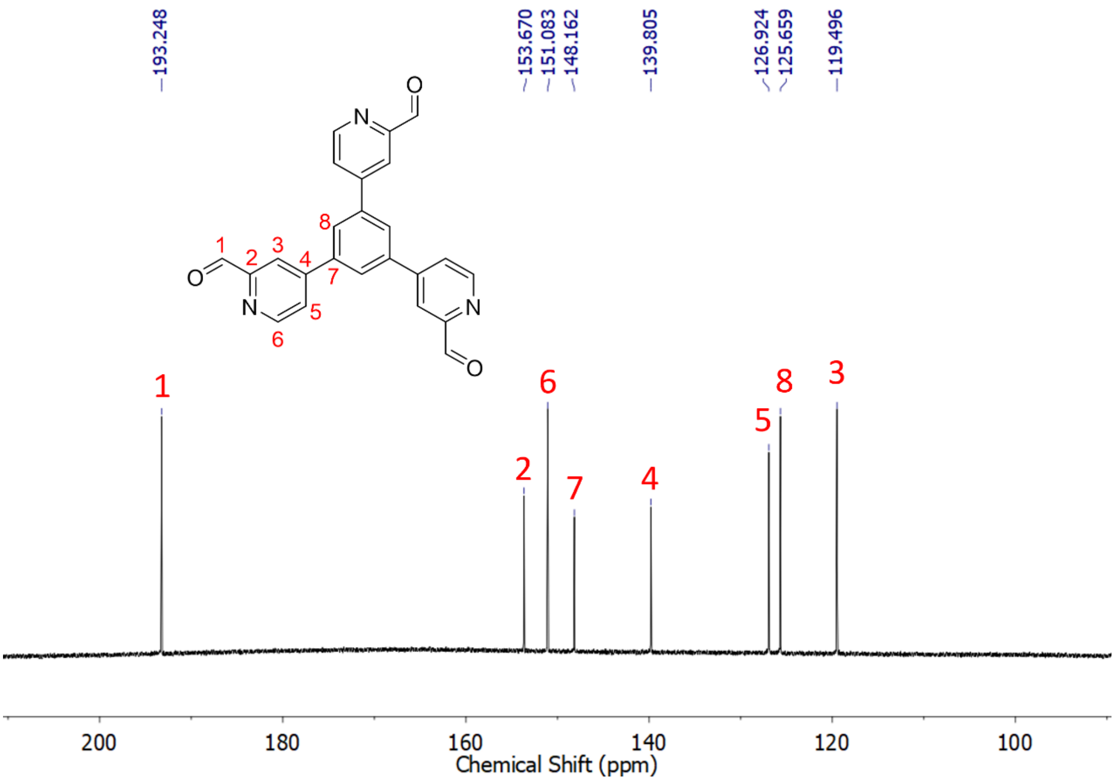

Figure S2. ^{13}C NMR spectrum of **A** (125 MHz, $CDCl_3$, 298 K).

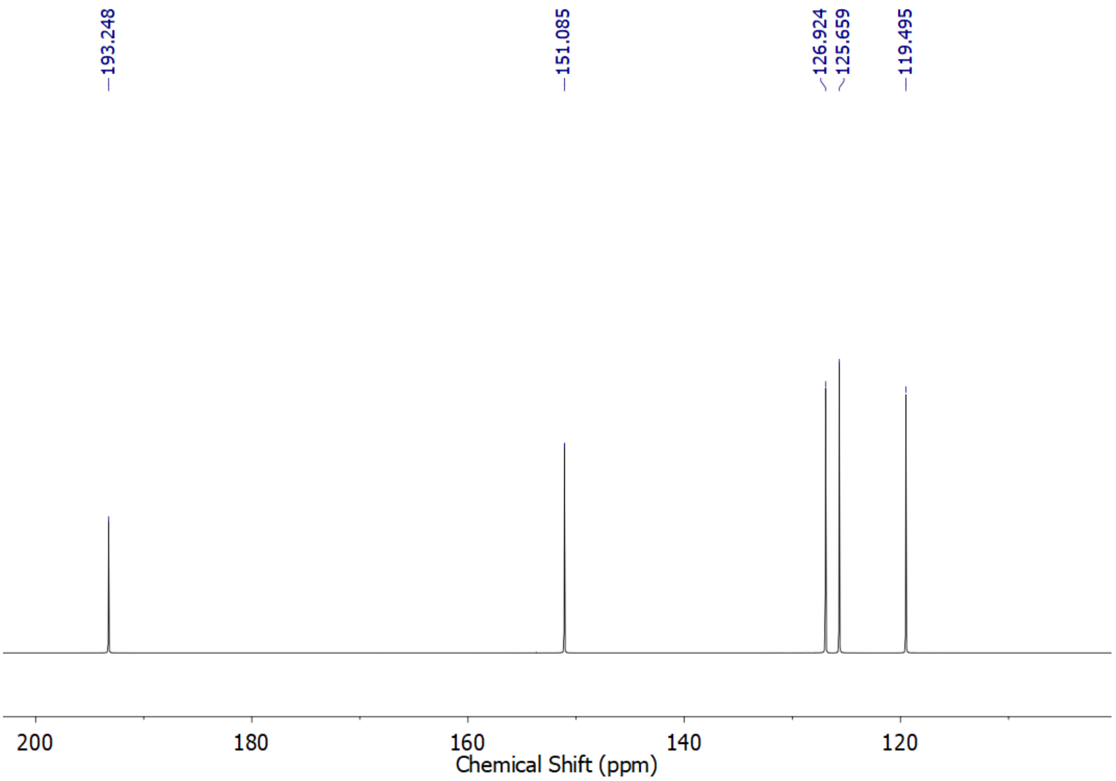

Figure S3. ^{13}C DEPT–135 NMR spectrum of **A** (125 MHz, $CDCl_3$, 298 K).

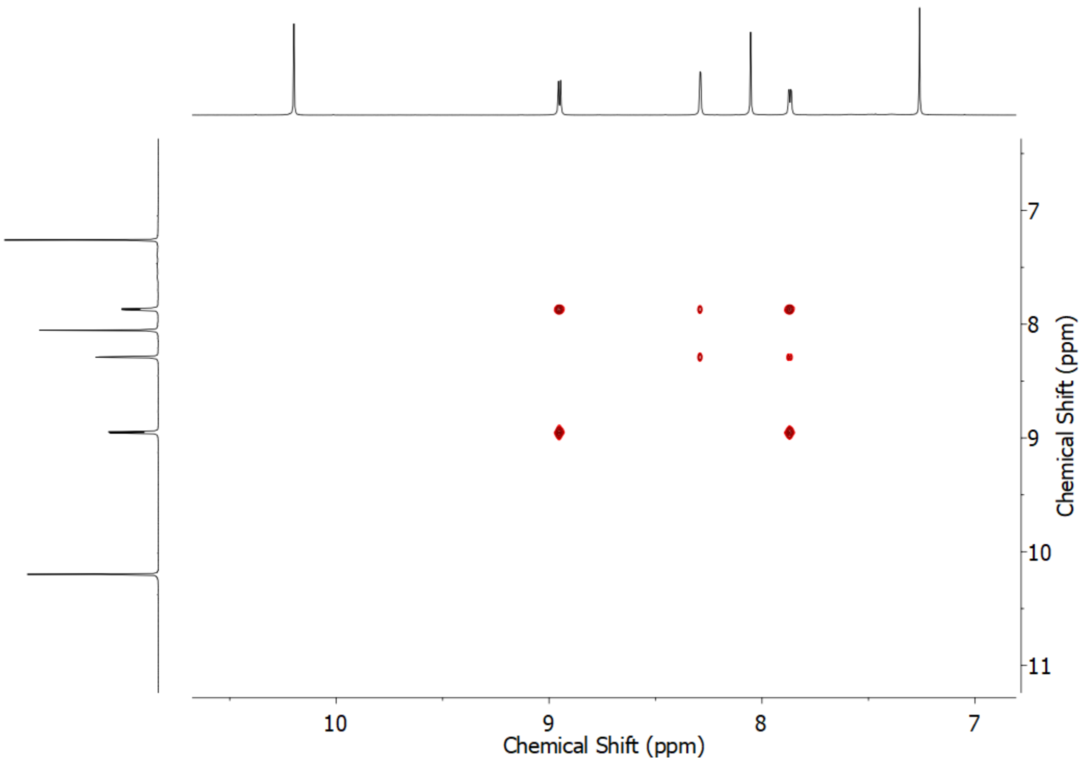

Figure S4. ^{1}H–^{1}H COSY NMR spectrum of **A** (500 MHz, $CDCl_3$, 298 K).

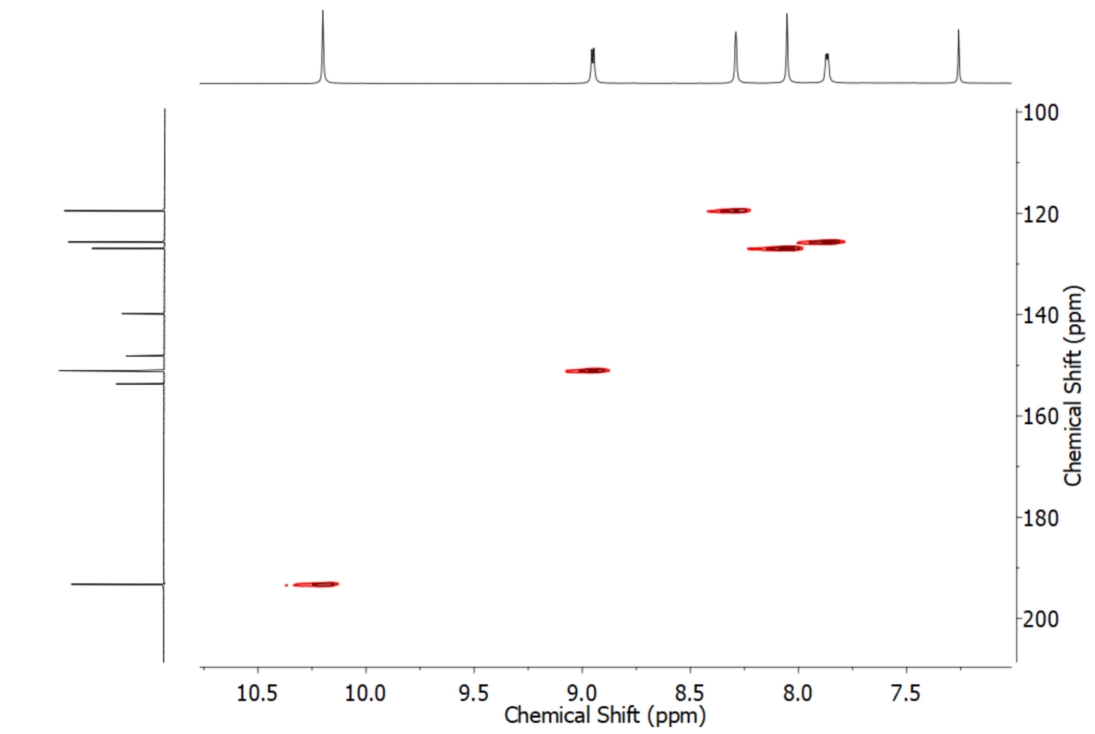

Figure S5. ^{1}H–^{13}C HSQC NMR spectrum of **A** (500 MHz, $CDCl_3$, 298 K).

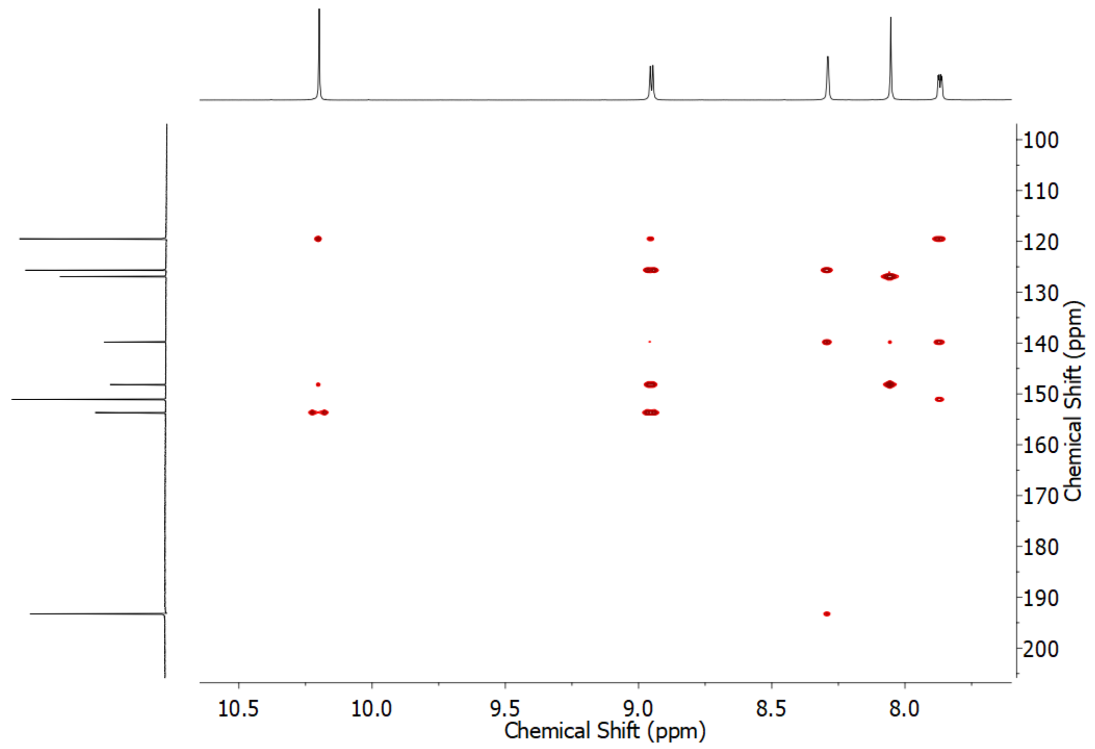

Figure S6. ^{1}H–^{13}C HMBC NMR spectrum of **A** (500 MHz, $CDCl_3$, 298 K).

3 Self-Assembly and Characterization of Cages 1 and 2

3.1 Self-Assembly and Characterization of Cage 1

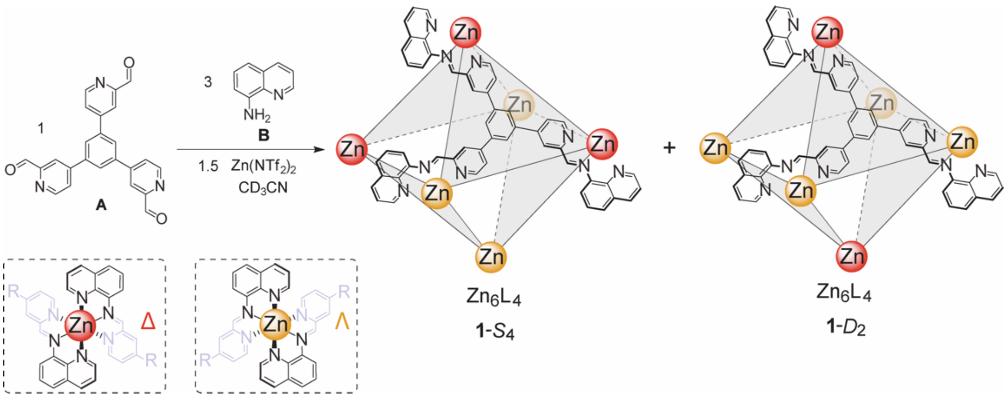

A (23.67 mg, 0.06 mmol, 4 equiv), 8-aminoquinoline (25.95 mg, 0.18 mmol, 12 equiv) and $Zn(NTf_2)_2$ (56.34 mg, 0.09 mmol, 6 equiv) were combined in CH_3CN (15 mL) in a 50 mL tube. The reaction mixture was sealed and stirred at 70 °C for 5 hours. The solvent was reduced to 0.7 mL, and Et_2O (30 mL) was then added. The precipitate was collected by centrifugation and washed with excess Et_2O, affording cage **1** as a dark brown solid (84.41 mg, 82%).

^{1}**H NMR** (500 MHz, CD_3CN): δ 10.05 (s, 0.5H), 10.03 (s, 1H), 10.00 (s, 0.5H), 9.99 (s, 1H), 9.72 (s, 0.5H), 9.69 (s, 1H), 8.96–8.79 (m, 5.5H), 8.68–8.47 (m, 9H), 8.46–8.29 (m, 8.5H), 8.28–8.18 (m, 4.5H), 8.17–8.09 (m, 4.5H), 8.09–7.96 (m, 4.5H), 7.76–7.72 (m, 1.5H), 7.70–7.62 (m, 3.0H), 7.60 –7.57 (dd, *J* = 8.0, 4.5 Hz, 1.0H), 7.53–7.50 (m, 1.0H), 7.50–7.47 (m, 1.0H), 7.46–7.43 (m, 1.0H).

^{13}**C NMR** (125 MHz, CD_3CN): δ 157.9, 157.8, 157.8, 152.1, 151.5, 151.4, 151.2, 151.1, 151.0, 150.3, 149.9, 149.8, 148.9, 148.8, 148.8, 148.7, 148.6, 141.4, 141.3, 141.3, 139.0, 138.4, 136.1, 135.99, 135.95, 132.7, 132.6, 132.5, 132.4, 130.7, 130.7, 130.6, 129.22, 129.16, 129.1, 129.0, 128.7, 128.6, 128.5, 128.3, 127.9, 127.5, 124.6, 124.5, 122.1, 120.6, 120.5, 119.5.

HR–ESI–MS: *m/z* = 575.0298, $[\mathbf{1}\cdot(NTf_2)_4]^{8+}$, (calc. 575.0555); 697.1657, $[\mathbf{1}\cdot(NTf_2)_5]^{7+}$, (calc. 697.2275); 860.1801, $[\mathbf{1}\cdot(NTf_2)_6]^{6+}$, (calc. 860.1236); 1088.2021, $[\mathbf{1}\cdot(NTf_2)_7]^{5+}$, (calc. 1088.1781); 1430.2286, $[\mathbf{1}\cdot(NTf_2)_8]^{4+}$, (calc. 1430.2599).

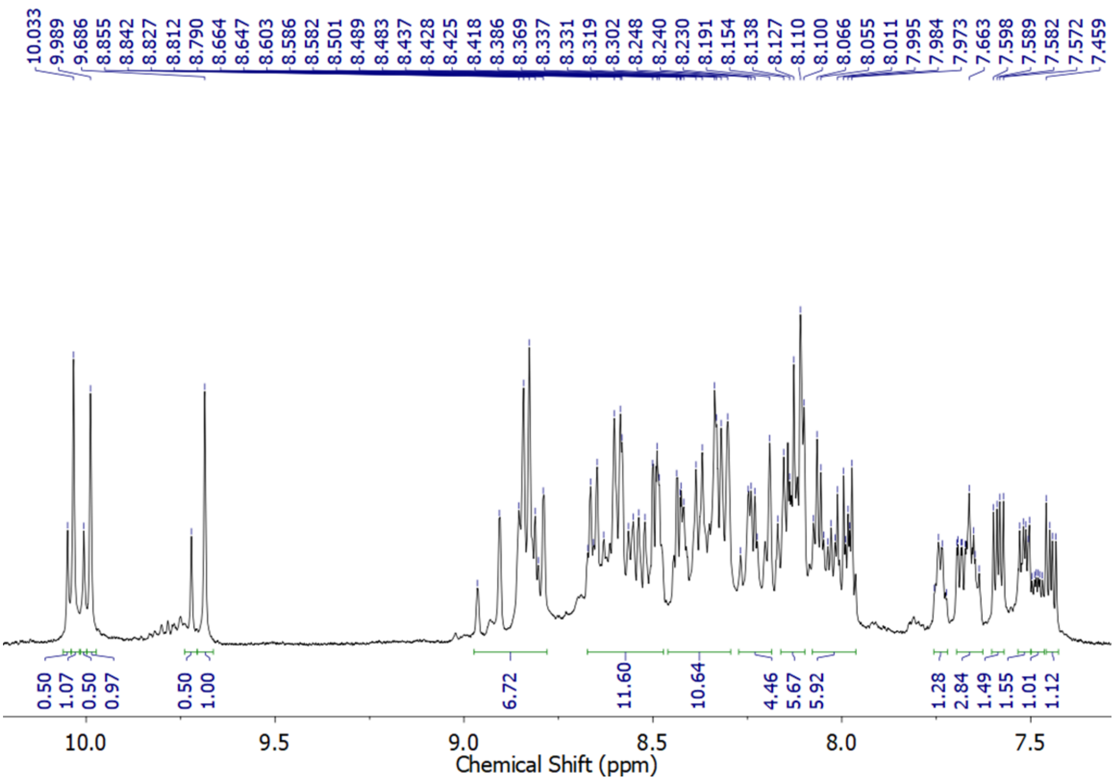

Figure S7. ^{1}H NMR spectrum of cage **1** (500 MHz, CD_3CN, 298 K) with the integrals of peaks shown.

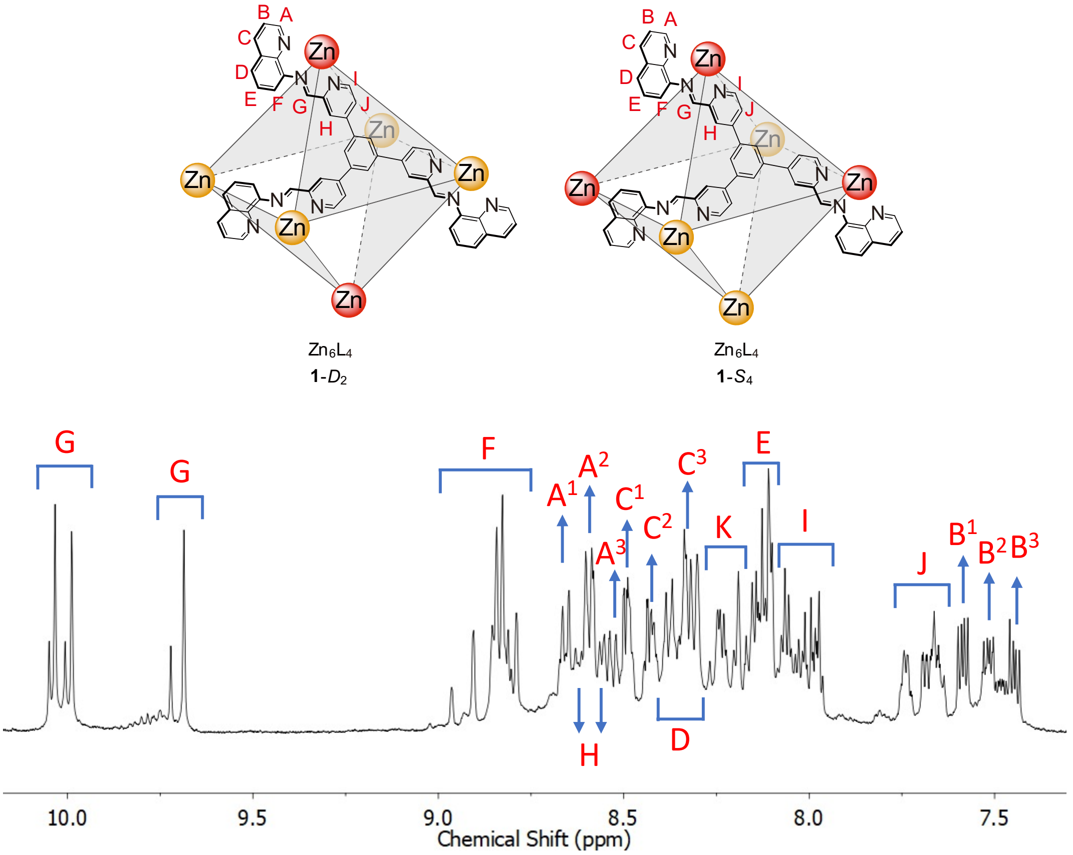

Figure S8. ^{1}H NMR spectrum of cage **1** (500 MHz, CD_3CN, 298 K) with the assignment of protons shown.

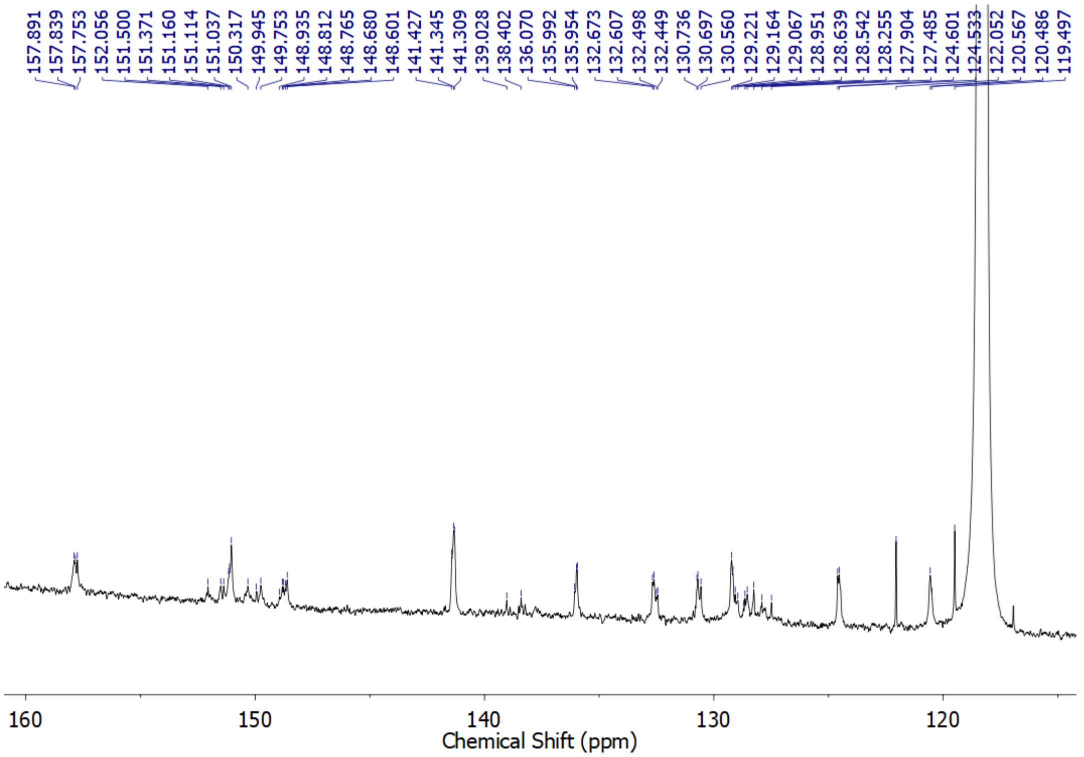

Figure S9. ^{13}C NMR spectrum of cage **1** (125 MHz, CD_3CN, 298 K).

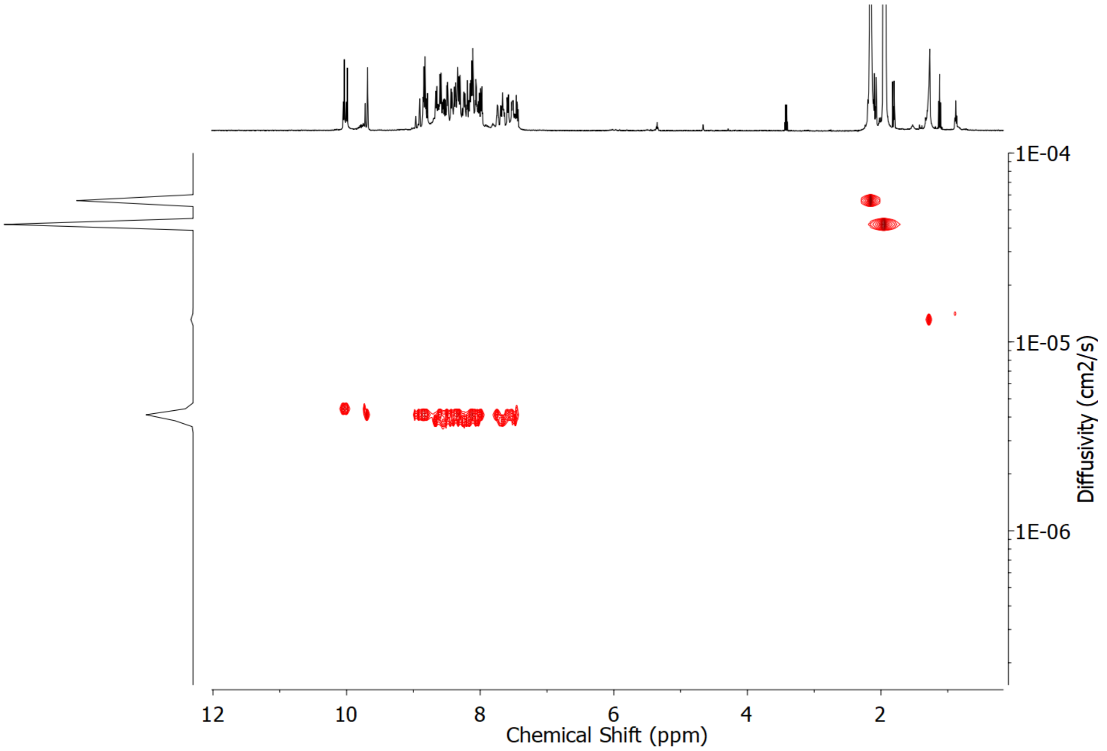

Figure S10. ^{1}H DOSY NMR spectrum of cage **1** (400 MHz, CD_3CN, 298 K). The diffusion coefficient for both diastereomers was measured to be 4.05×10^{-6} cm^2/s.

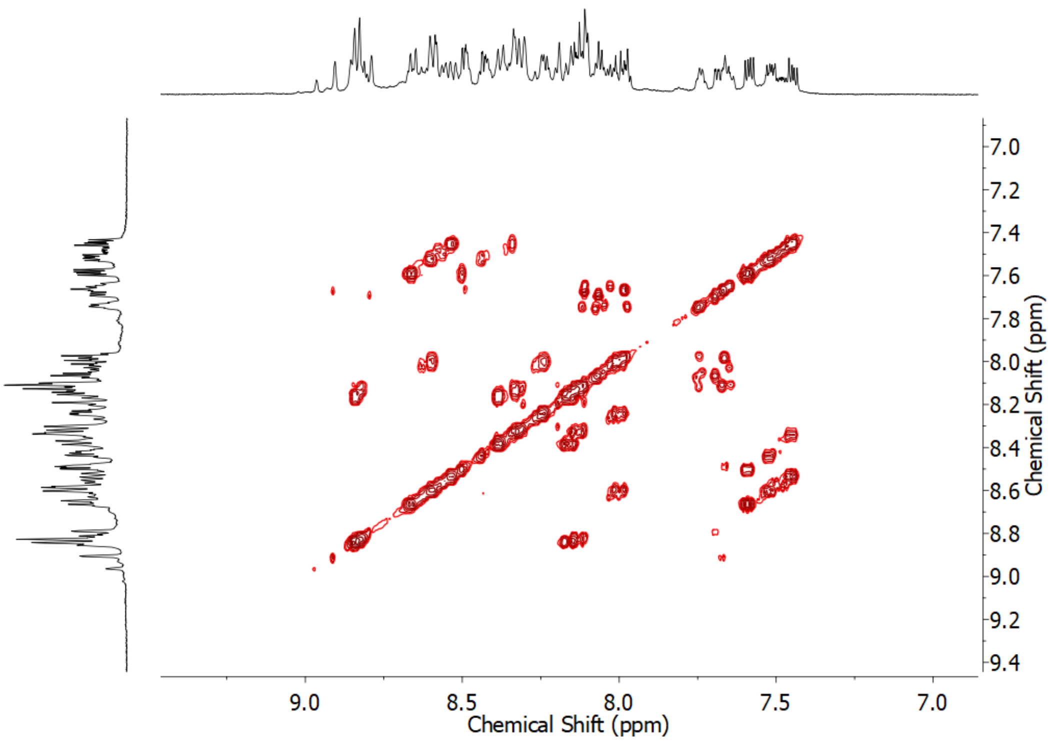

Figure S11. ^1H–^1H COSY NMR spectrum of cage **1** (500 MHz, CD_3CN, 298 K).

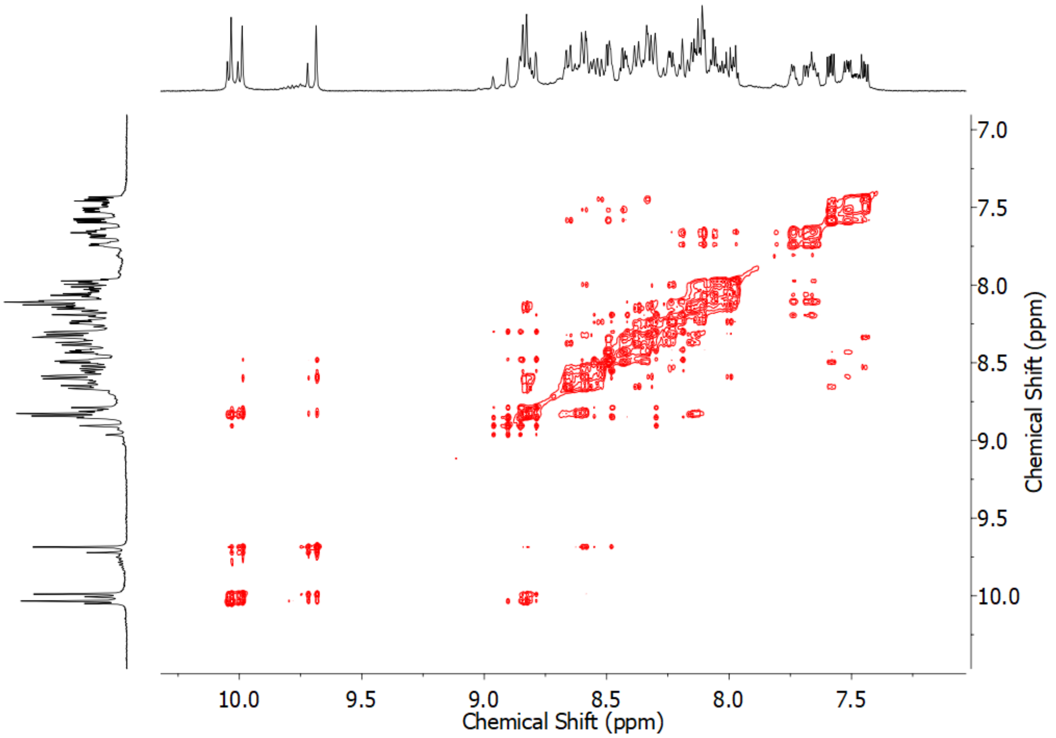

Figure S12. ^1H–^1H NOESY NMR spectrum of cage **1** (500 MHz, CD_3CN, 298 K).

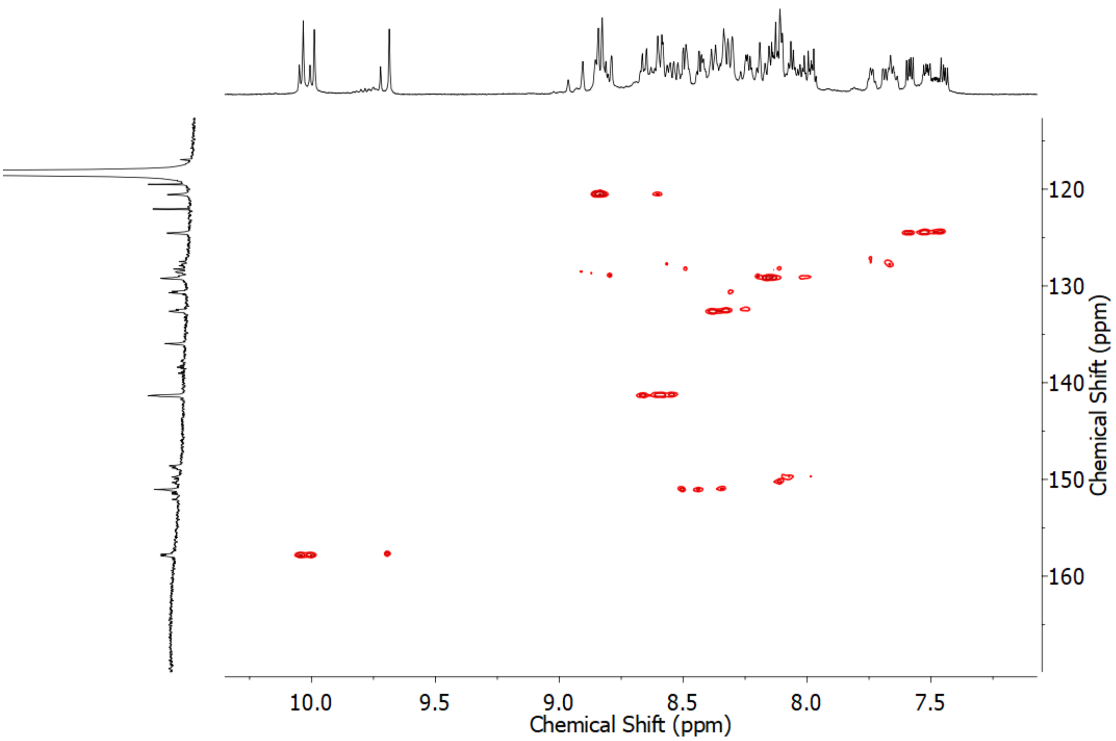

Figure S13. ^{1}H–^{13}C HSQC NMR spectrum of cage **1** (500 MHz, CD_3CN, 298 K).

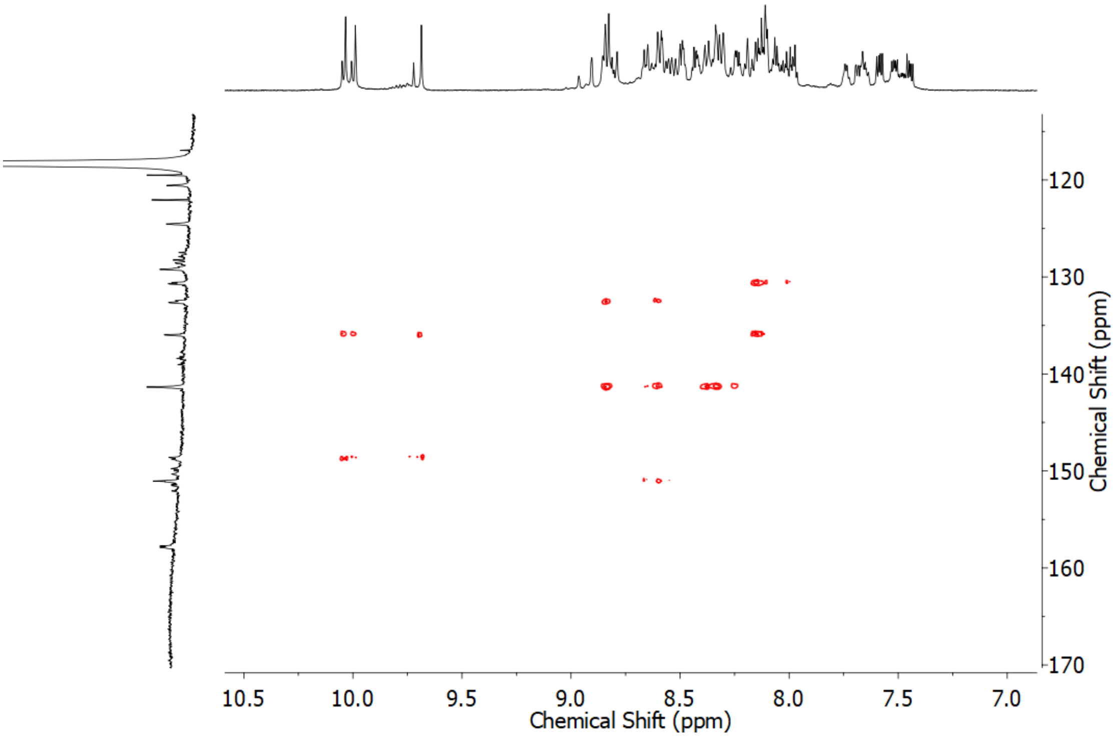

Figure S14. ^{1}H–^{13}C HMBC NMR spectrum of cage **1** (500 MHz, CD_3CN, 298 K).

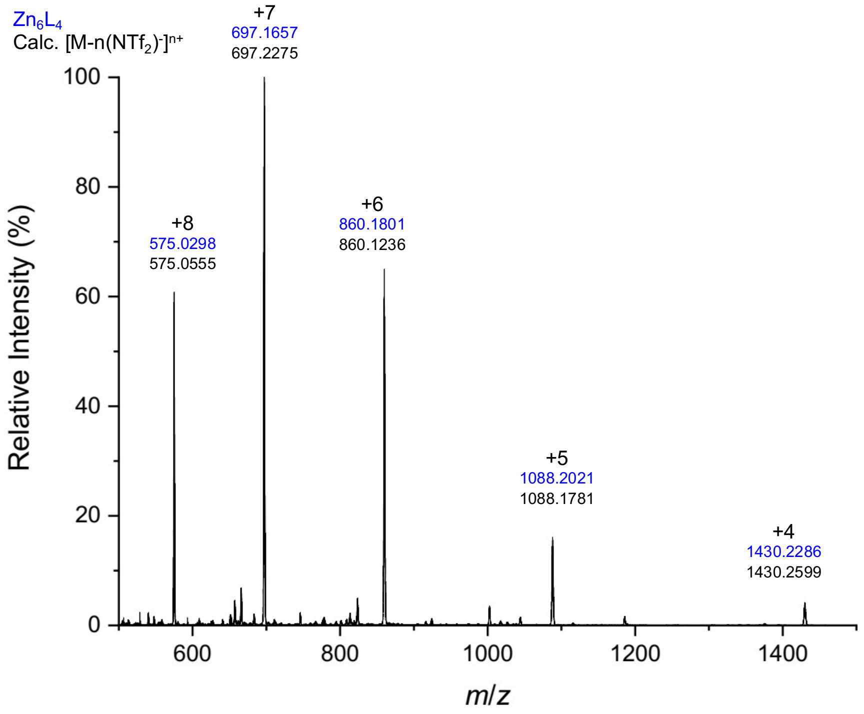

Figure S15. HR–ESI–MS spectrum of cage **1** in CH_3CN. Experimental (blue) and calculated (black) *m/z* values for each charge state are shown.

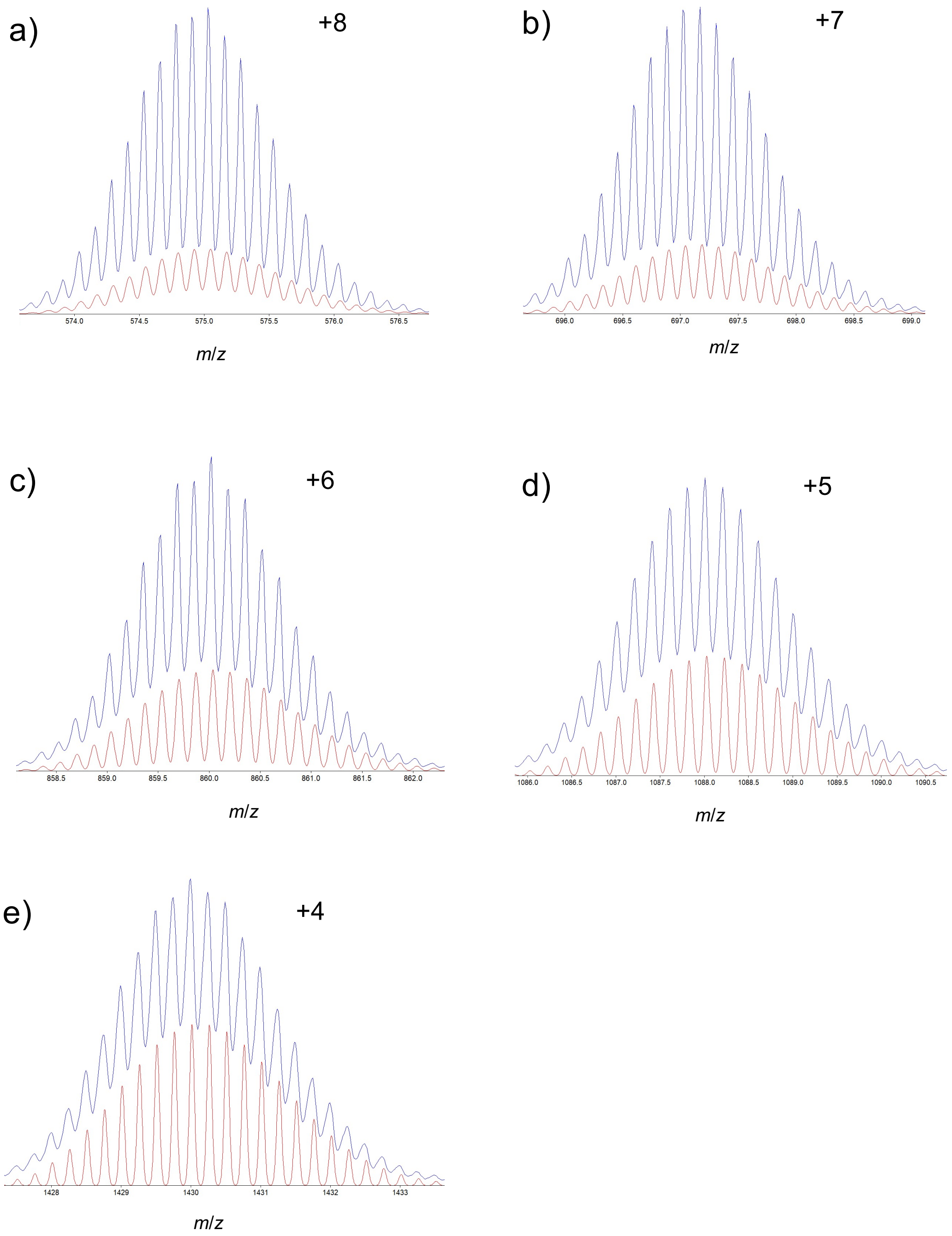

Figure S16. HR–ESI–MS spectra of cage **1** in CH_3CN. Experimental (blue) and calculated (red) peaks for a) [**1**·$(NTf_2)_4]^{8+}$; b) [**1**·$(NTf_2)_5]^{7+}$; c) [**1**·$(NTf_2)_6]^{6+}$; d) [**1**·$(NTf_2)_7]^{5+}$; e) [**1**·$(NTf_2)_8]^{4+}$.

3.2 Self-Assembly and Characterization of Cage 2

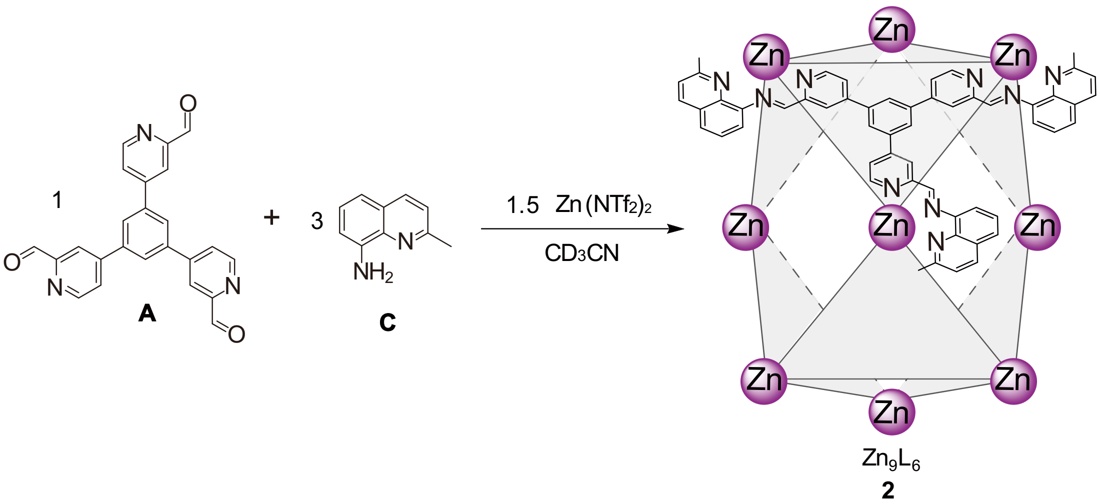

A (23.6 mg, 0.06 mmol, 6 equiv), 2-methyl-8-aminoquinoline **C** (28.48 mg, 0.12 mmol, 18 equiv) and $Zn(NTf_2)_2$ (56.32 mg, 0.09 mmol, 9 equiv) were combined in CH_3CN (3.0 mL) in a 15 mL tube. The reaction mixture was sealed and stirred at 70 °C overnight. The solvent was reduced to 0.3 mL, and Et_2O (10 mL) was then added. The precipitate was collected by centrifugation and washed with excess diethyl ether, affording cage **2** as a dark solid (98.65 mg, 91%).

^{13}C NMR (125 MHz, CD_3CN): δ 163.7, 163.4, 157.0, 150.3, 148.2, 141.6, 141.2, 134.7, 133.1, 129.4, 128.4, 126.6, 121.0, 24.9. Due to the presence of multiple isomers and low symmetry of cage **2**, many ^{13}C signals overlap, thus fewer than the expected number of signals are observed.

HR–ESI–MS: *m/z* = 771.2788, [**1**·$(NTf_2)_8$]$^{10+}$, (calc. 771.3443); 888.0794, [**1**·$(NTf_2)_9$]$^{9+}$, (calc. 888.1769); 1034.2069, [**1**·$(NTf_2)_{10}$]$^{8+}$, (calc. 1034.2176); 1221.9398, [**1**·$(NTf_2)_{11}$]$^{7+}$, (calc. 1221.9842); 1472.2504, [**1** · $(NTf_2)_{12}$]$^{6+}$, (calc. 1472.3398); 1822.6828, [**1** · $(NTf_2)_{13}$]$^{5+}$, (calc. 1822.8375).

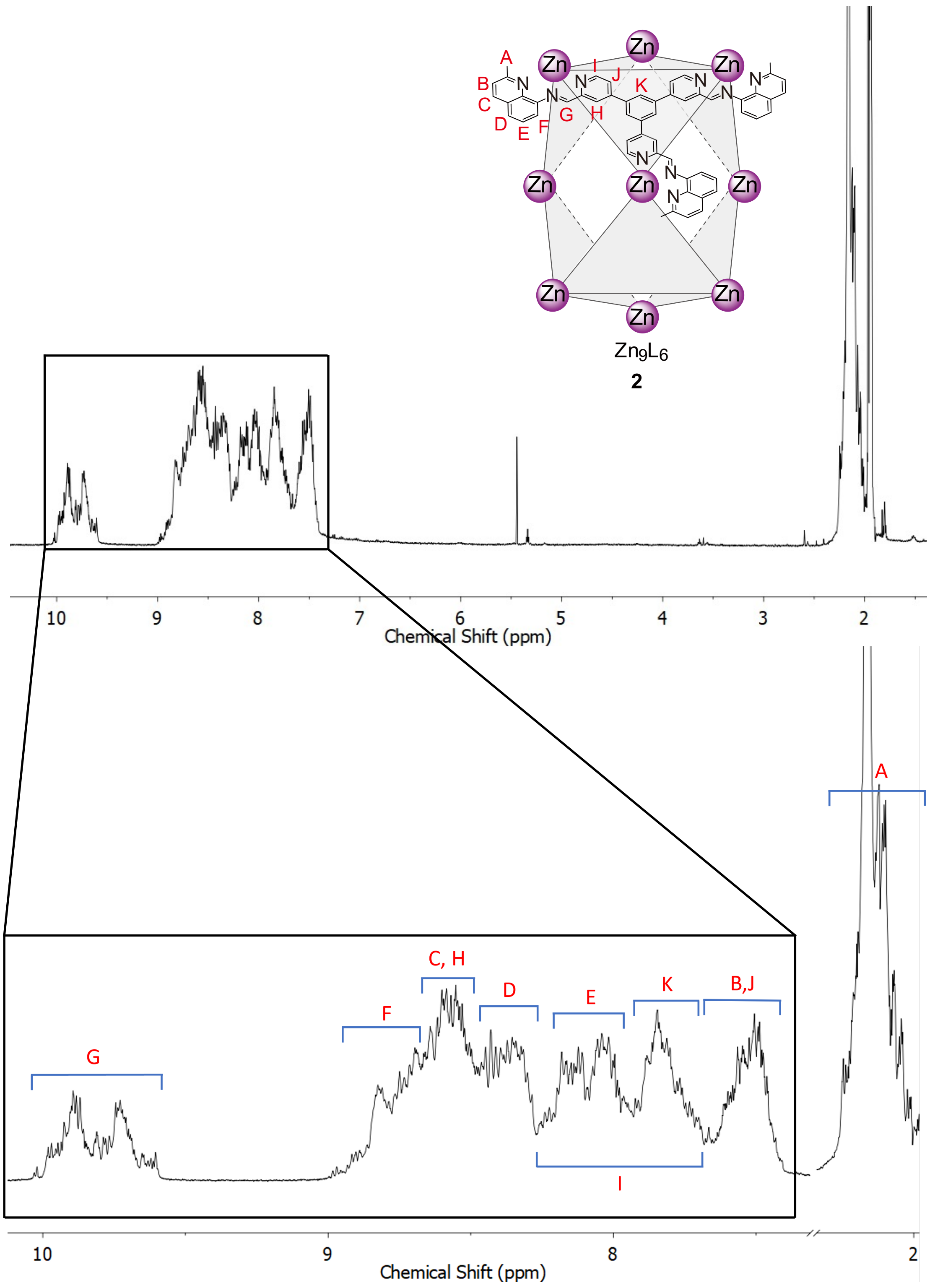

Figure S17. ^{1}H NMR spectrum of cage **2** (500 MHz, CD_3CN, 298 K) with assignments of protons shown.

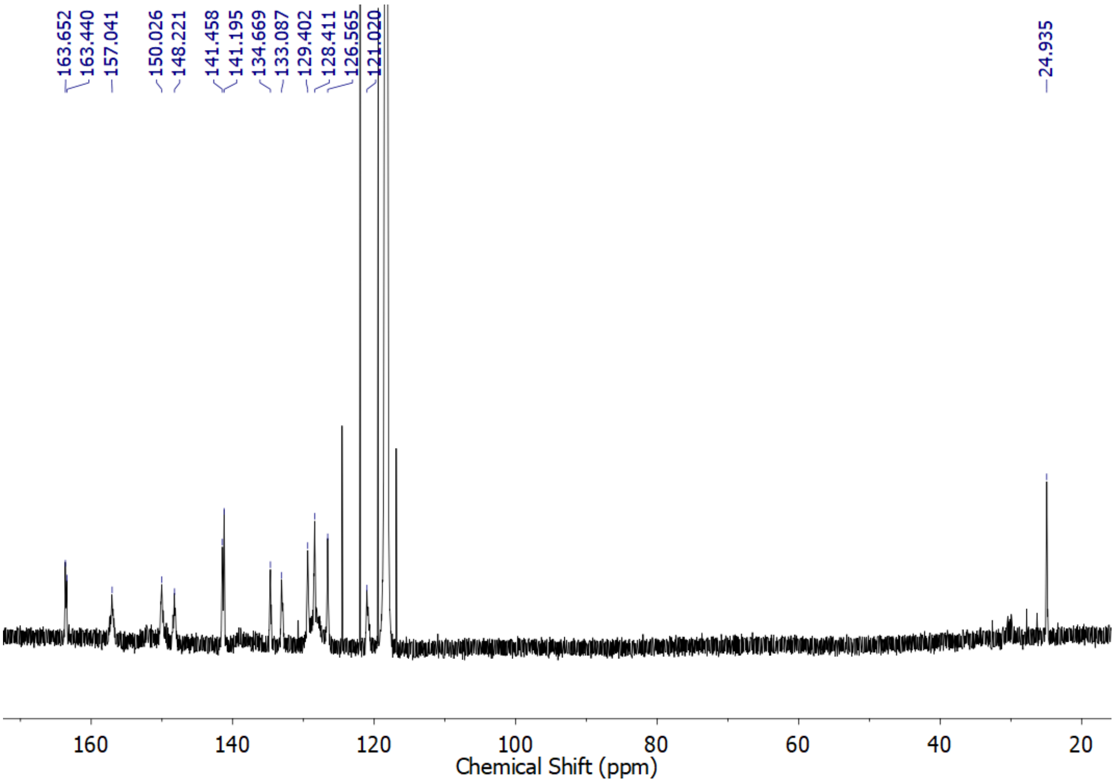

Figure S18. ^{13}C NMR spectrum of cage **2** (125 MHz, CD_3CN, 298 K).

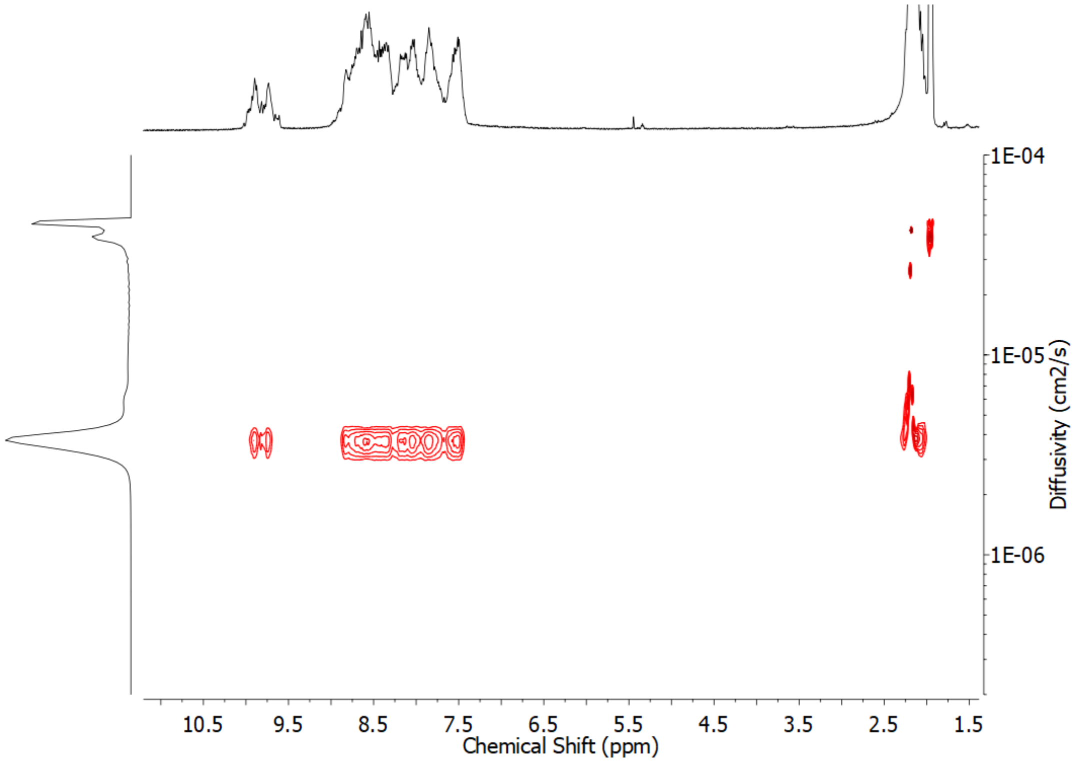

Figure S19. ^{1}H DOSY spectrum of cage **2** (400 MHz, CD_3CN, 298 K). The diffusion coefficient for all diastereomers was measured to be 3.75×10^{-6} cm^2/s.

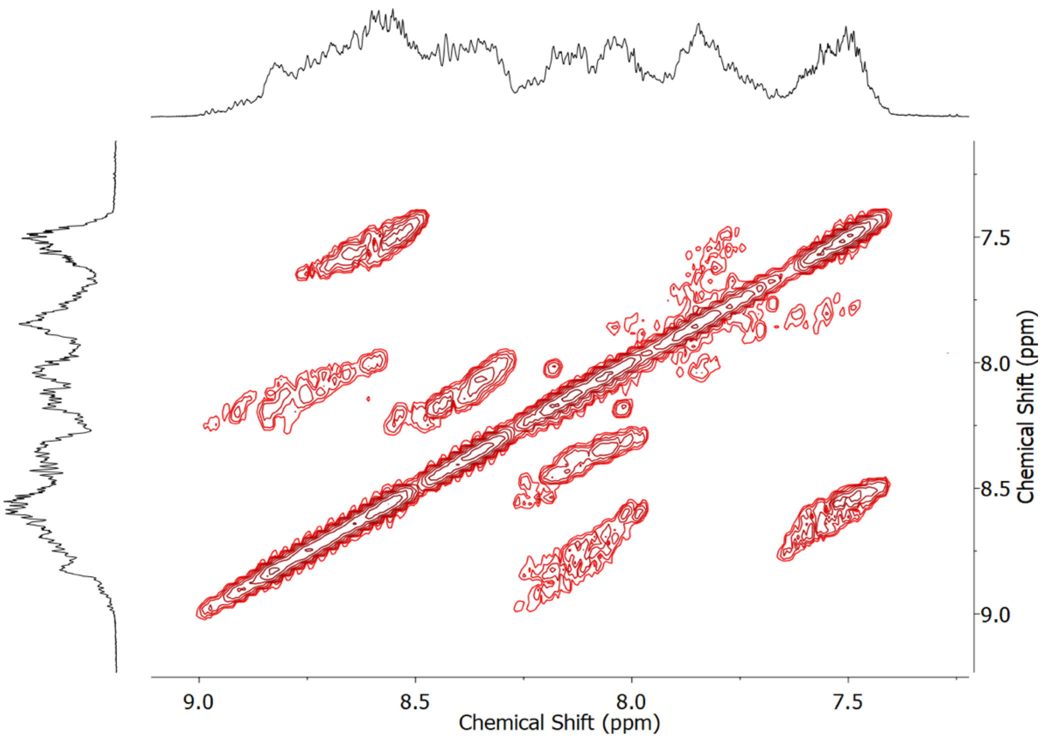

Figure S20. ^1H–^1H COSY NMR spectrum of cage **2** (500 MHz, CD_3CN, 298 K).

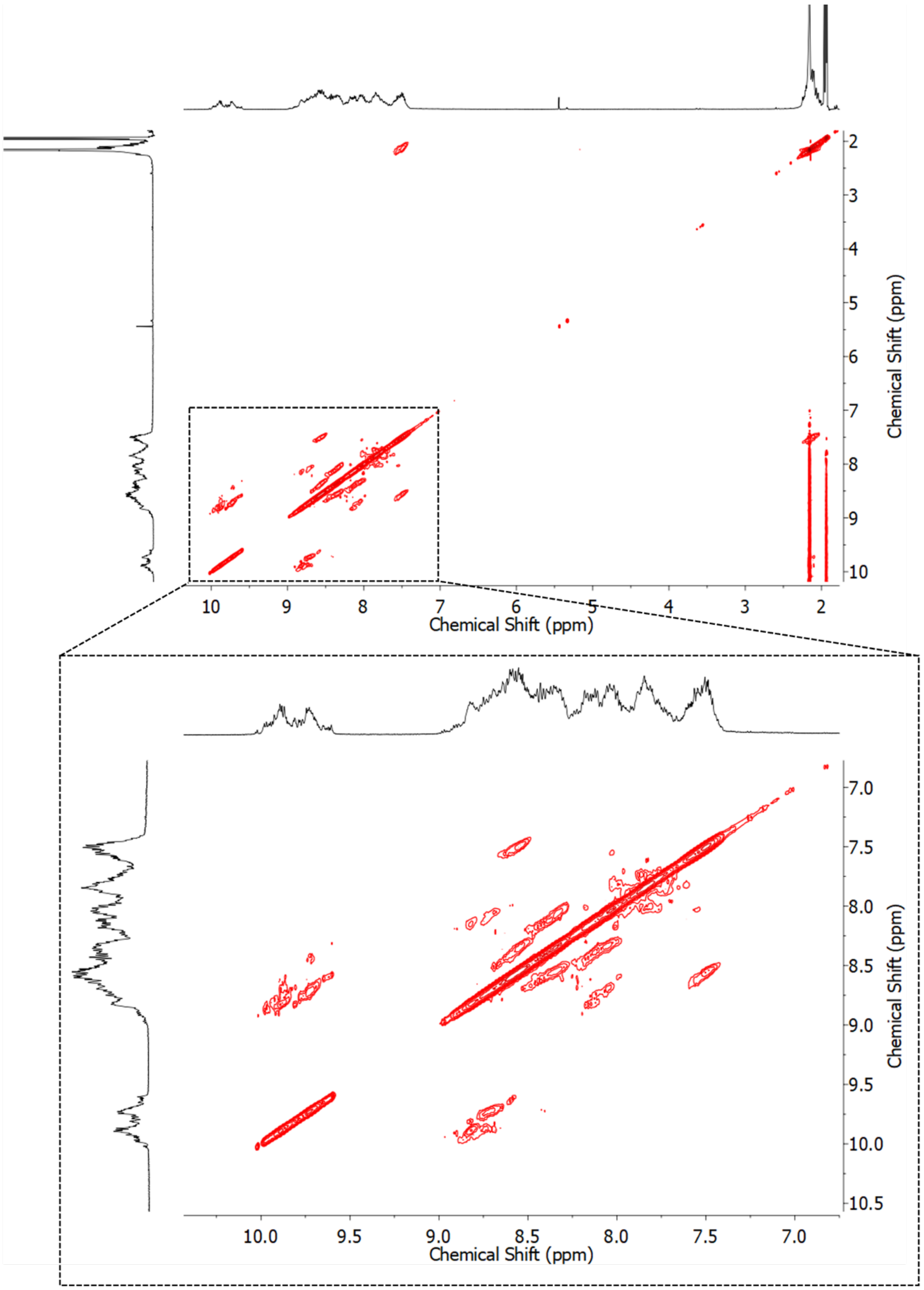

Figure S21. ^1H–^1H NOESY NMR spectrum of cage **2** (500 MHz, CD_3CN, 298 K).

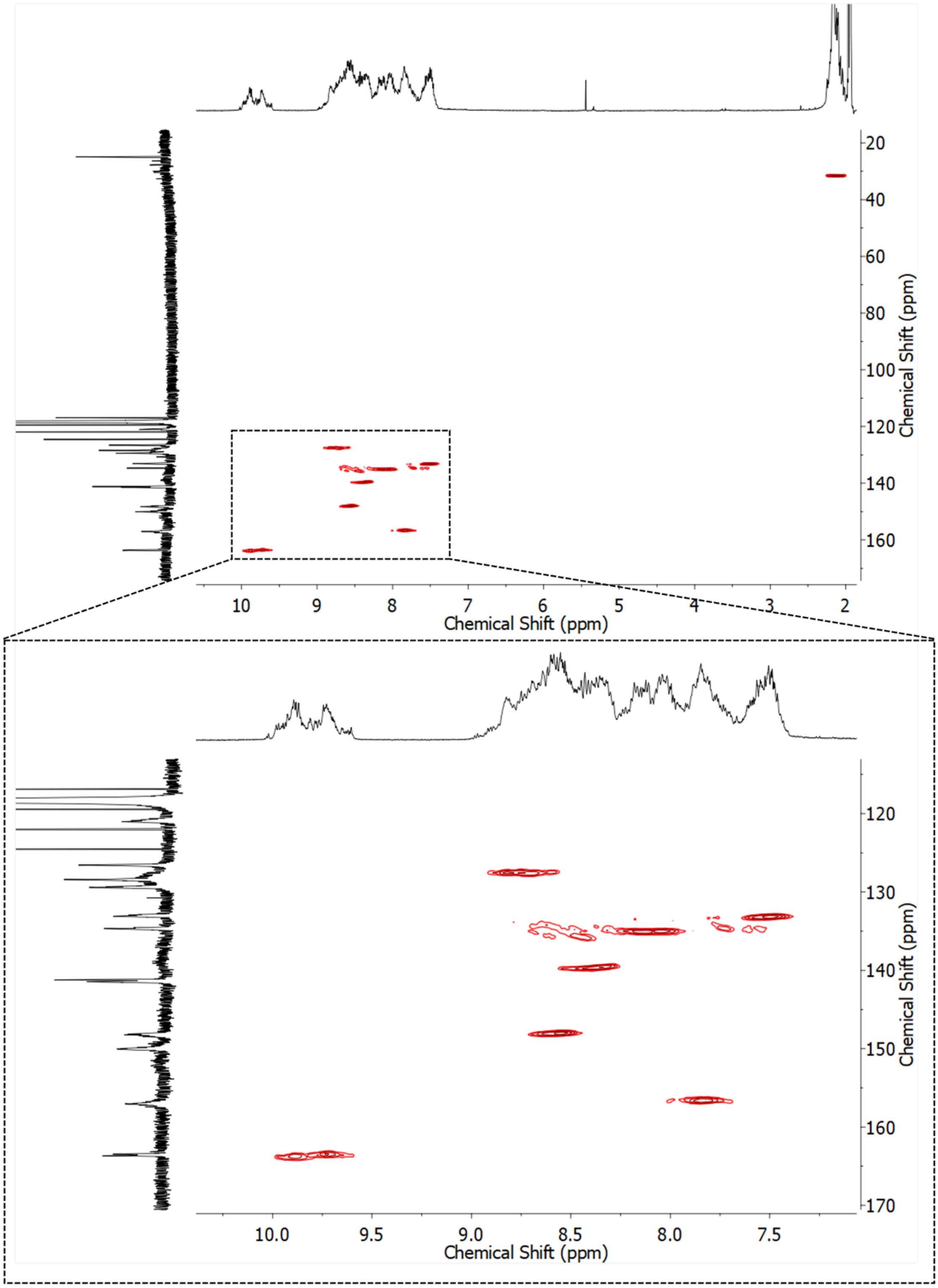

Figure S22. ^{1}H–^{13}C HSQC NMR spectrum of cage **2** (500 MHz, CD_3CN, 298 K).

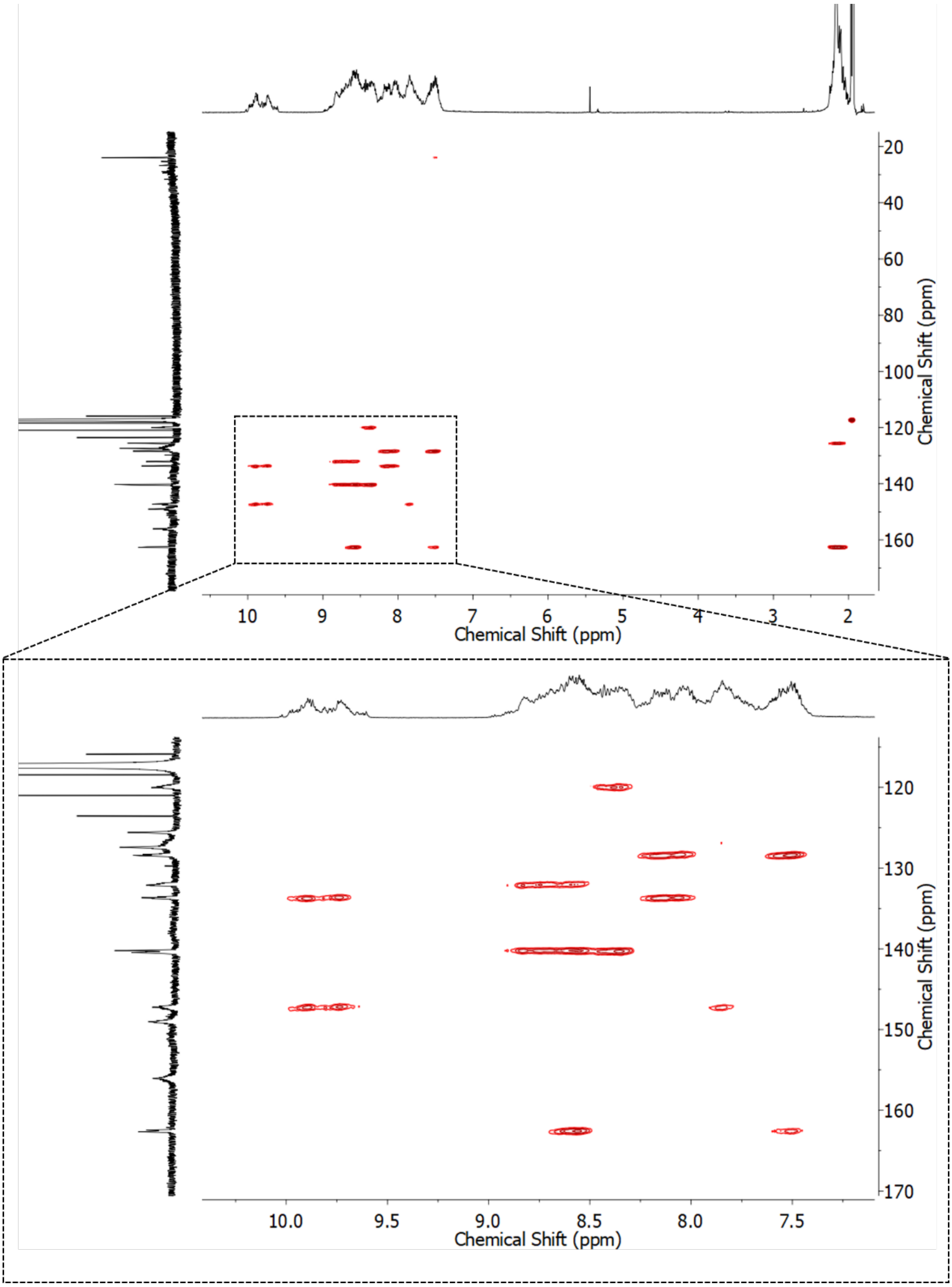

Figure S23. ^{1}H–^{13}C HMBC NMR spectrum of cage **2** (500 MHz, CD_3CN, 298 K).

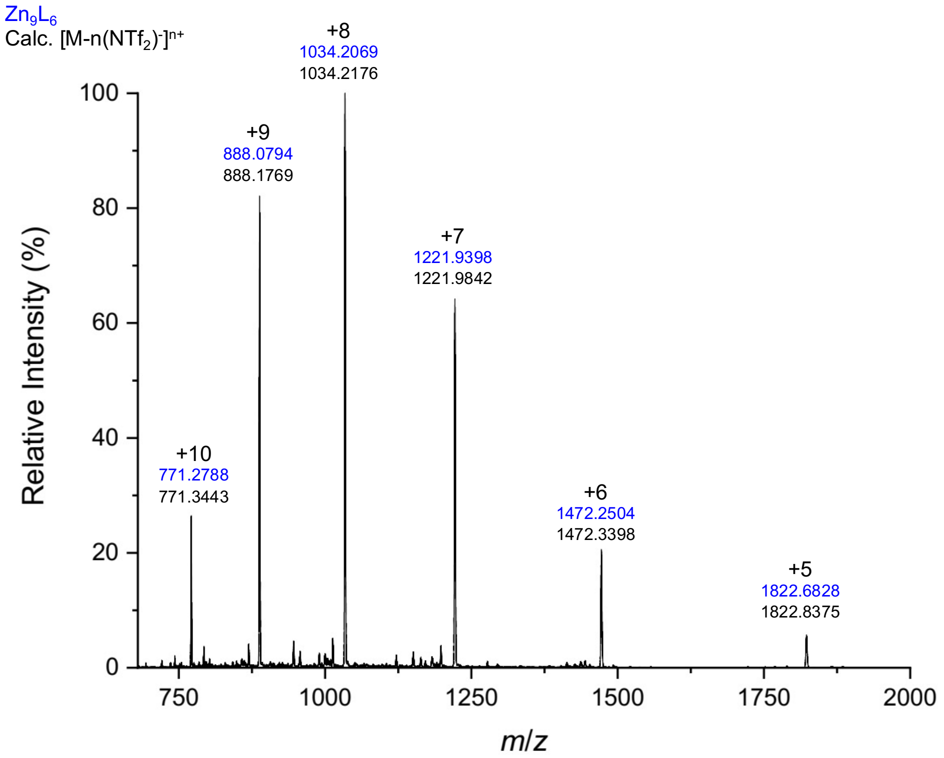

Figure S24. HR–ESI–MS spectrum of cage **2** in CH_3CN. Experimental (blue) and calculated (black) peaks.

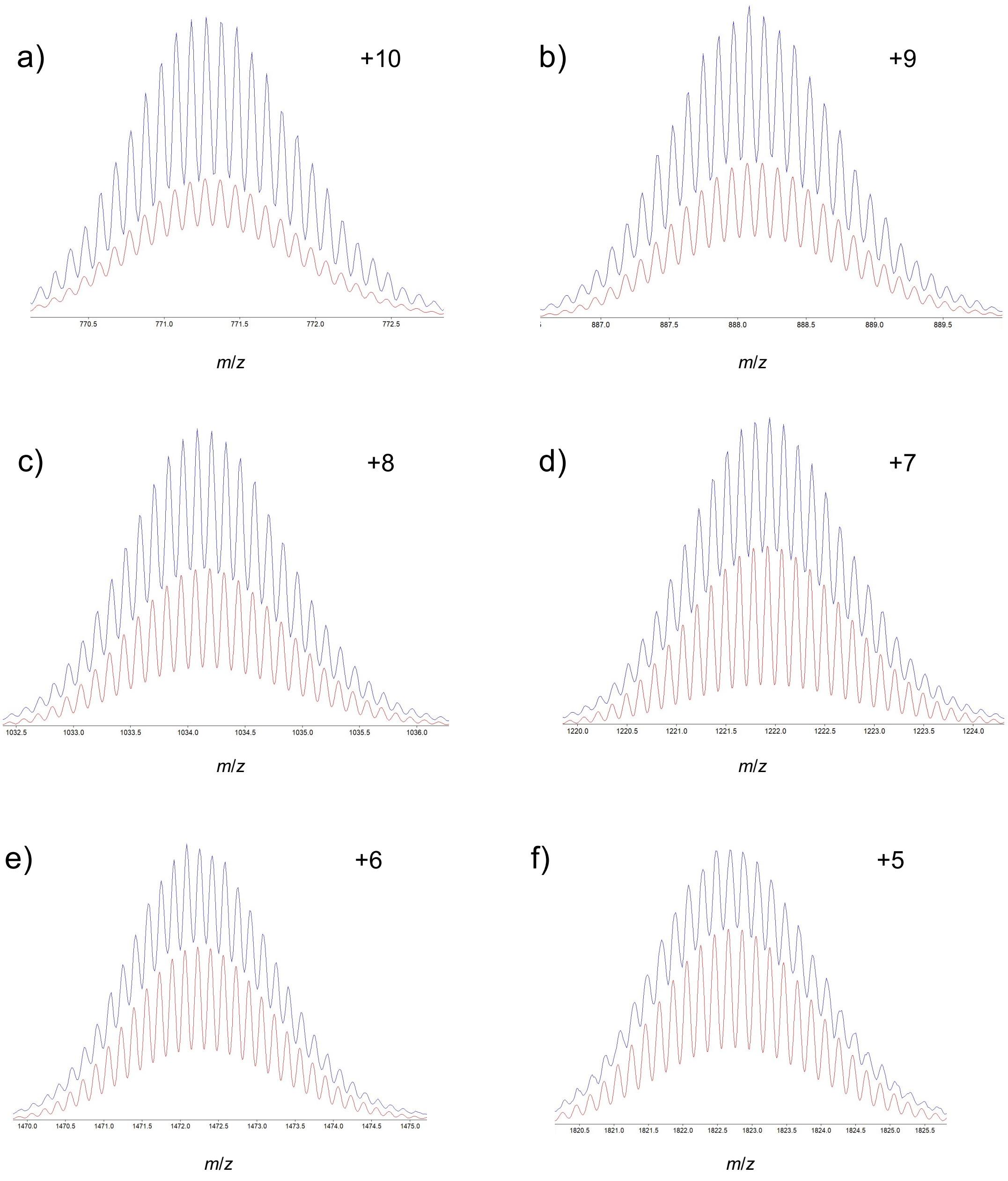

Figure S25. HR–ESI–MS spectra of cage **2** in CH_3CN. Experimental (blue) and calculated (red) peaks for a) [**2**·$(NTf_2)_8$]$^{10+}$; b) [**2**·$(NTf_2)_9$]$^{9+}$; c) [**2**·$(NTf_2)_{10}$]$^{8+}$; d) [**2**·$(NTf_2)_{11}$]$^{7+}$; e) [**2**·$(NTf_2)_{12}$]$^{6+}$; f) [**2**·$(NTf_2)_{13}$]$^{5+}$.

4 Conversion of $Zn^{II}{}_9L_6$ to $Zn^{II}{}_6L_4$

4.1 Templates Exploration to Convert $Zn^{II}{}_9L_6$ into $Zn^{II}{}_6L_4$

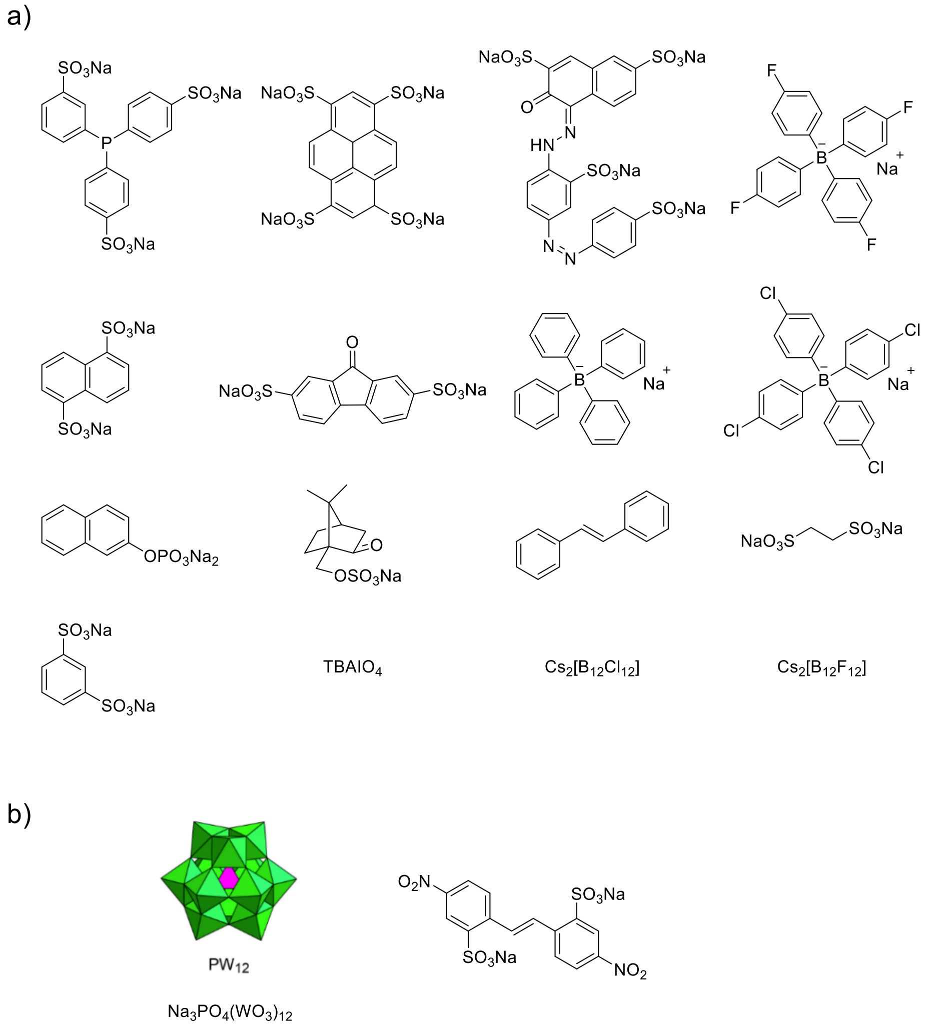

Figure S26. a) No conversion was observed of $Zn^{II}{}_9L_6$ to $Zn^{II}{}_6L_4$ after adding one of these guests. b) These guests drive the conversion of $Zn^{II}{}_9L_6$ to $Zn^{II}{}_6L_4$.

4.2 Trianionic Dodecatungstophosphate as a Template

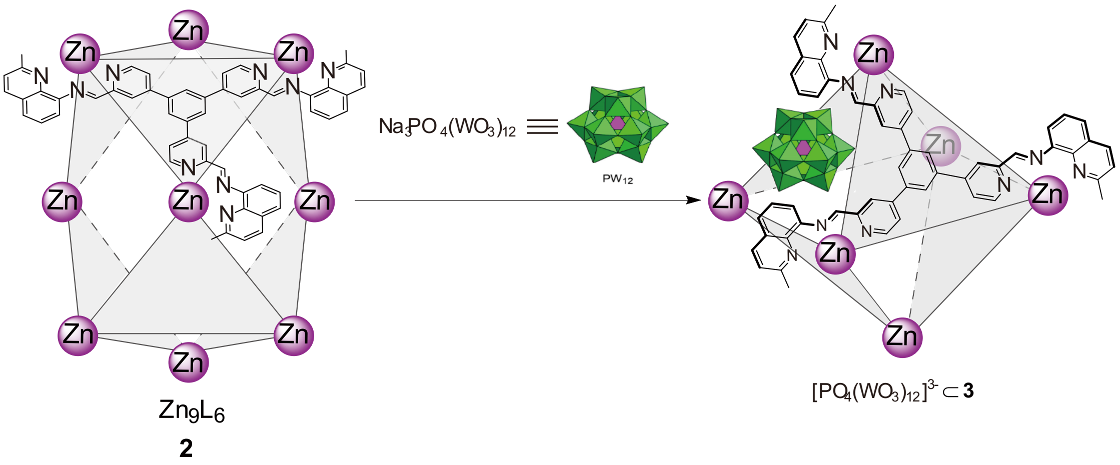

Cage **2** (1.60 mg, 1.0 equiv), sodium dodecatungstophosphate hydrate (1.13 mg, 1.2 equiv) were combined in CD_3CN (0.5 mL) in a 5 mL tube. The reaction mixture was sealed and stirred with 1200 rds at 70 °C for 5 days. The host–guest complex $[PO_4(WO_3)_{12}]^{3-}\subset$**3** with low solubility was obtained.

HR–ESI–MS: *m/z* = 850.6223, [**3**·$(PO_4(WO_3)_{12})(NTf_2)]^{8+}$, (calc. 850.6711); 1012.1293, [**3**·$(PO_4(WO_3)_{12})(NTf_2)_2]^{7+}$, (calc. 1012.2169); 1227.6374, [**3**·$(PO_4(WO_3)_{12})(NTf_2)_3]^{6+}$, (calc. 1227.6112); 1529.1494, [**3**·$(PO_4(WO_3)_{12})(NTf_2)_4]^{5+}$, (calc. 1529.1632); 1981.4172, [**3**·$(PO_4(WO_3)_{12})(NTf_2)_5]^{4+}$, (calc. 1981.4913).

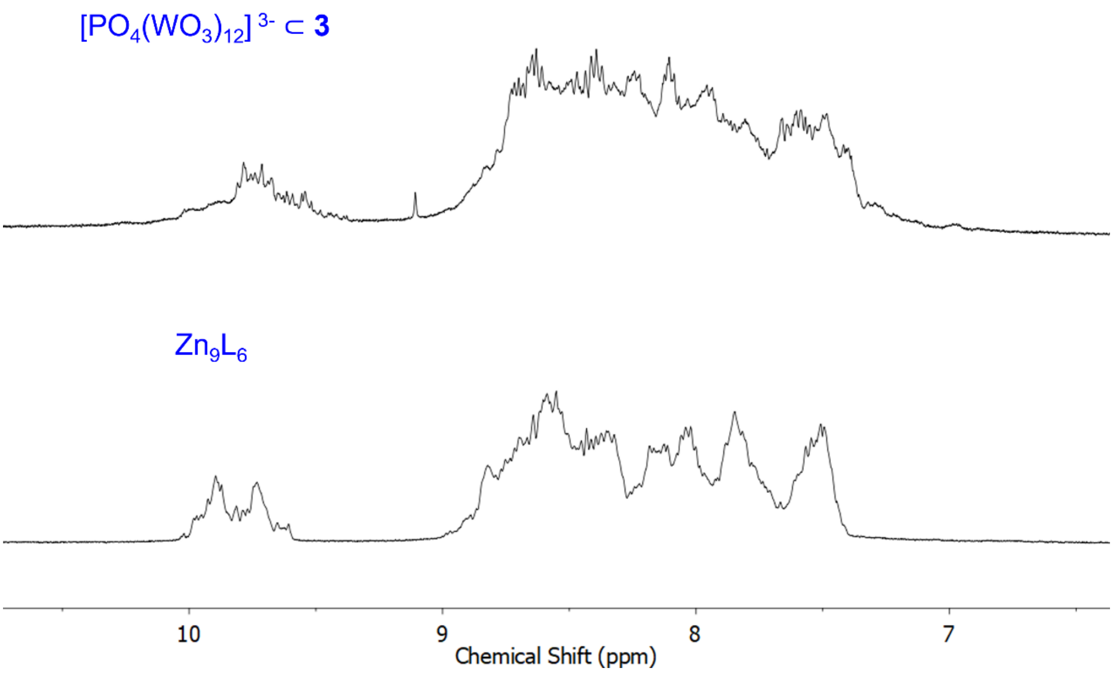

Figure S27. ^{1}H NMR spectra (500 MHz, CD_3CN, 298 K) of $[PO_4(WO_3)_{12}]^{3-}$⊂**3** (top spectrum), compared to the free cage **2** (bottom spectrum).

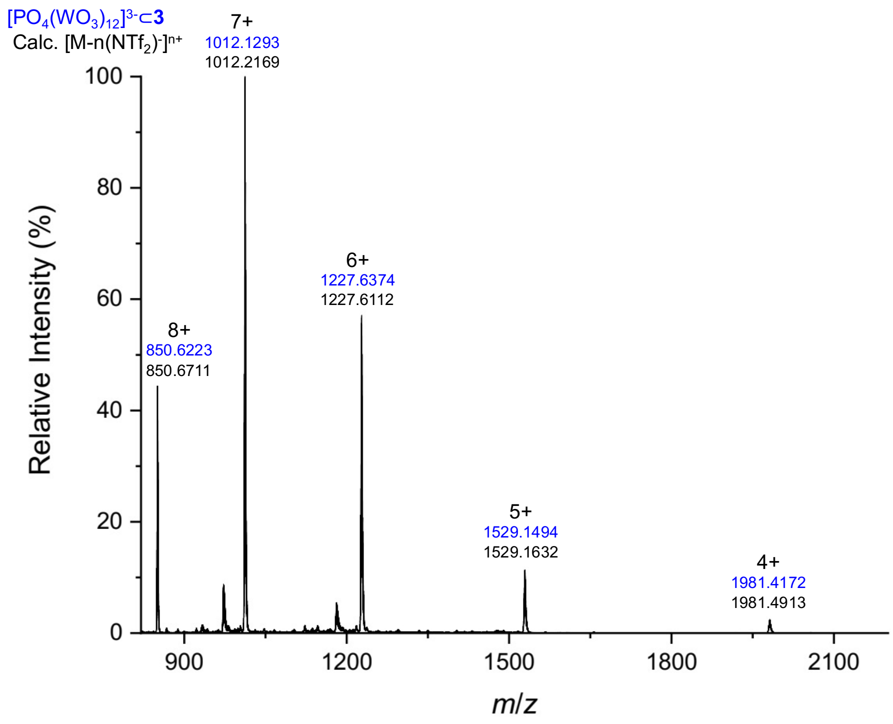

Figure S28. HR–ESI–MS spectrum of $[PO_4(WO_3)_{12}]^{3-}$⊂**3** in CH_3CN. Experimental (blue) and calculated (black) peaks.

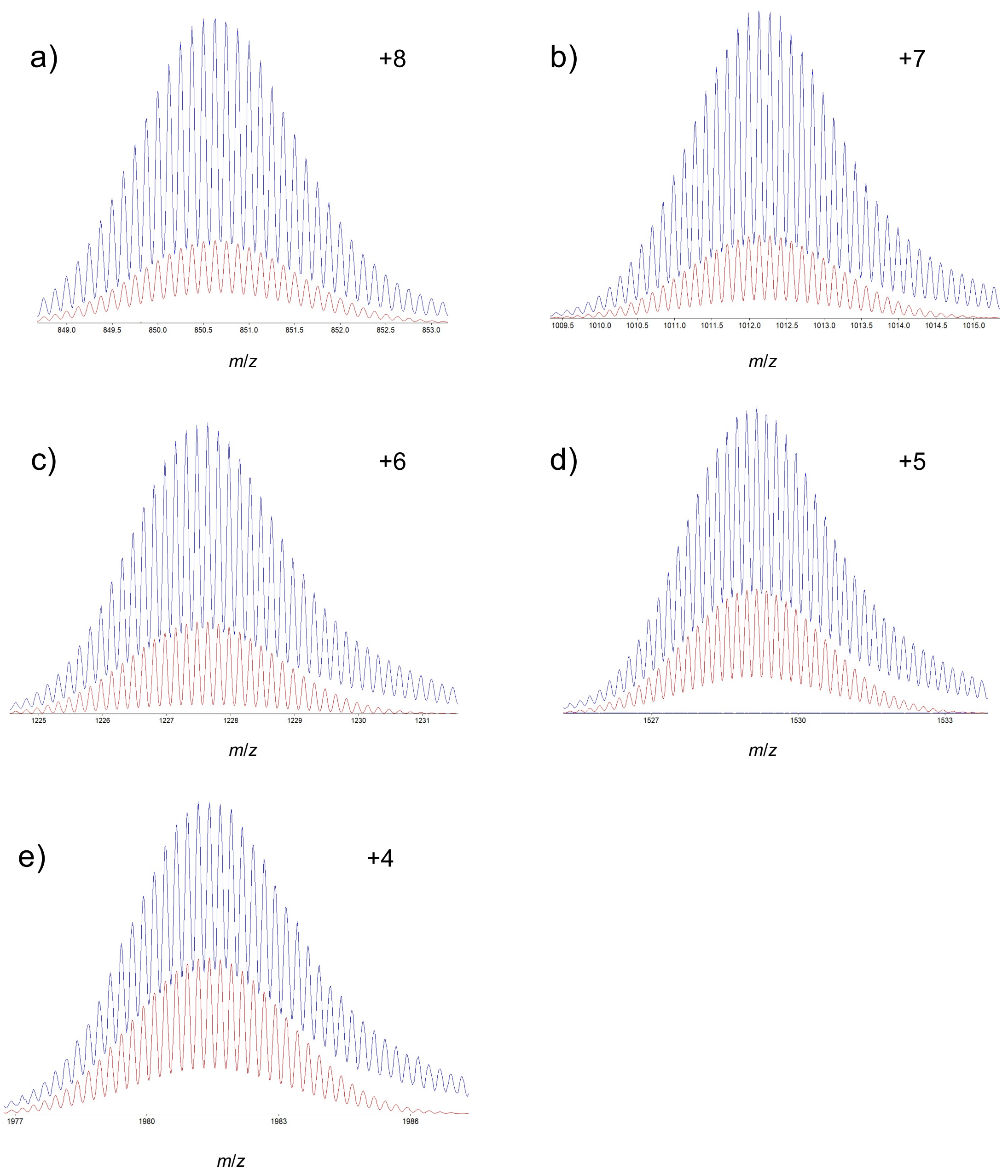

Figure S29. HR–ESI–MS spectra of $[PO_4(WO_3)_{12}]^{3-}\subset$**3** in CH_3CN. Experimental (blue) and calculated (red) peaks for a) [**3**·$(NTf_2)(PO_4(WO_3)_{12})]^{8+}$; b) [**3**·$(NTf_2)_2(PO_4(WO_3)_{12})]^{7+}$; c) [**3**·$(NTf_2)_3(PO_4(WO_3)_{12})]^{6+}$; d) [**3**·$(NTf_2)_4(PO_4(WO_3)_{12})]^{5+}$; e) [**3**·$(NTf_2)_5(PO_4(WO_3)_{12})]^{4+}$.

4.3 Dianionic 4,4’-Dinitrostilbene-2,2’-disulfonate as a Template

4.3.1 Characterization of (G)$_4$⊂3-S_4

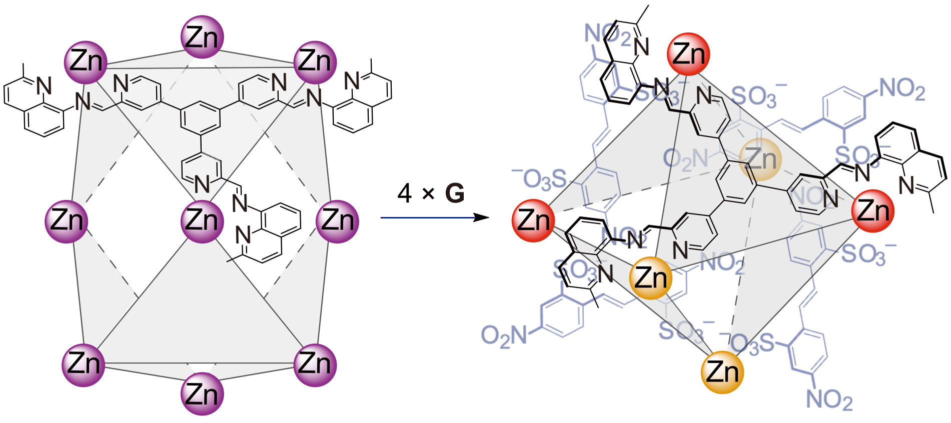

Zn_9L_6
2

(**G**)$_4$⊂**3**-S_4

Cage **2** (3.0 mg), disodium 4,4’-dinitrostilbene-2,2’-disulfonate (**G**, 3.0 mg, 0.12 mmol, 18 equiv) were combined in CD_3CN (0.5 mL) in a 5 mL tube. The reaction mixture was sealed and stirred with 1200 rds at 70 °C for 6 h. The complex (**G**)$_4$⊂**3**-S_4 with bad solubility was obtained.

^{1}H NMR (500 MHz, CD_3CN): δ 10.94 (s, 1H), 10.54 (s, 1H), 10.28 (s, 1H), 9.23 – 9.19 (m, 2H), 9.09 (s, 1H), 9.01 (d, J = 9.5 Hz, 1H), 8.93 (s, 1H), 8.83 (s, 1H), 8.54 – 8.52 (m, 2H), 8.49 – 8.48 (m, 2H), 8.43 (d, J = 10.5 Hz, 1H), 8.38 – 8.34 (m, 3H), 8.21 – 8.17 (m, 2H), 8.13 – 8.12 (m, 2H), 7.97 (d, J = 10.5 Hz, 1H), 7.88 – 7.83 (m, 2H), 7.75 (d, J = 6.0 Hz, 1H), 7.62 – 7.58 (m, 3H), 7.53 – 7.33 (m, 8H), 7.14 (d, J = 7.0 Hz, 1H), 5.93 (d, J = 6.0 Hz, 1H), 2.15 (s, 1H), 2.14 (s, 1H), 2.01 (s, 1H).

^{13}C NMR (125 MHz, CD_3CN): δ 163.5, 163.3, 163.1, 161.4, 159.1, 158.6, 151.4, 150.8, 150.5, 149.1, 149.0, 148.6, 148.6, 148.2, 147.1, 141.8, 141.4, 141.32, 141.26, 141.2, 141.0, 140.9, 139.8, 138.9, 136.2, 135.3, 135.1, 134.8, 132.3, 131.9, 131.4, 131.1, 129.8, 129.6, 129.0, 129.0, 128.9, 128.8, 128.1, 128.1, 127.9, 127.4, 127.3, 126.8, 126.7, 126.3, 126.2, 126.1, 124.7, 123.0, 122.3, 122.0, 121.6, 24.9, 24.8, 24.7.

HR–ESI–MS: m/z = 1340.4283, [**3** · (**G**)$_4$]$^{4+}$, (calc. 1340.3965); 1880.5372, [**3** · (**G**)$_4$(NTf_2)]$^{3+}$, (calc. 1880.5783). **G** = 4,4’-dinitrostilbene-2,2’-disulfonate.

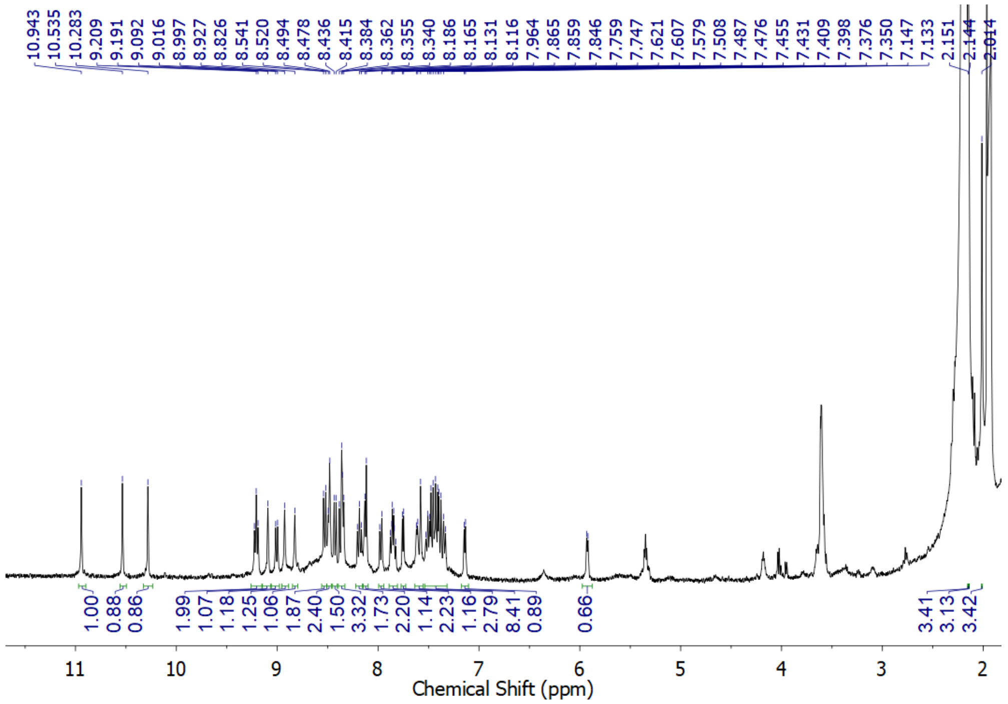

Figure S30. ^{1}H NMR spectrum of (**G**)$_4$⊂**3**-S_4 (500 MHz, CD_3CN, 298 K) with the integrals of peaks shown.

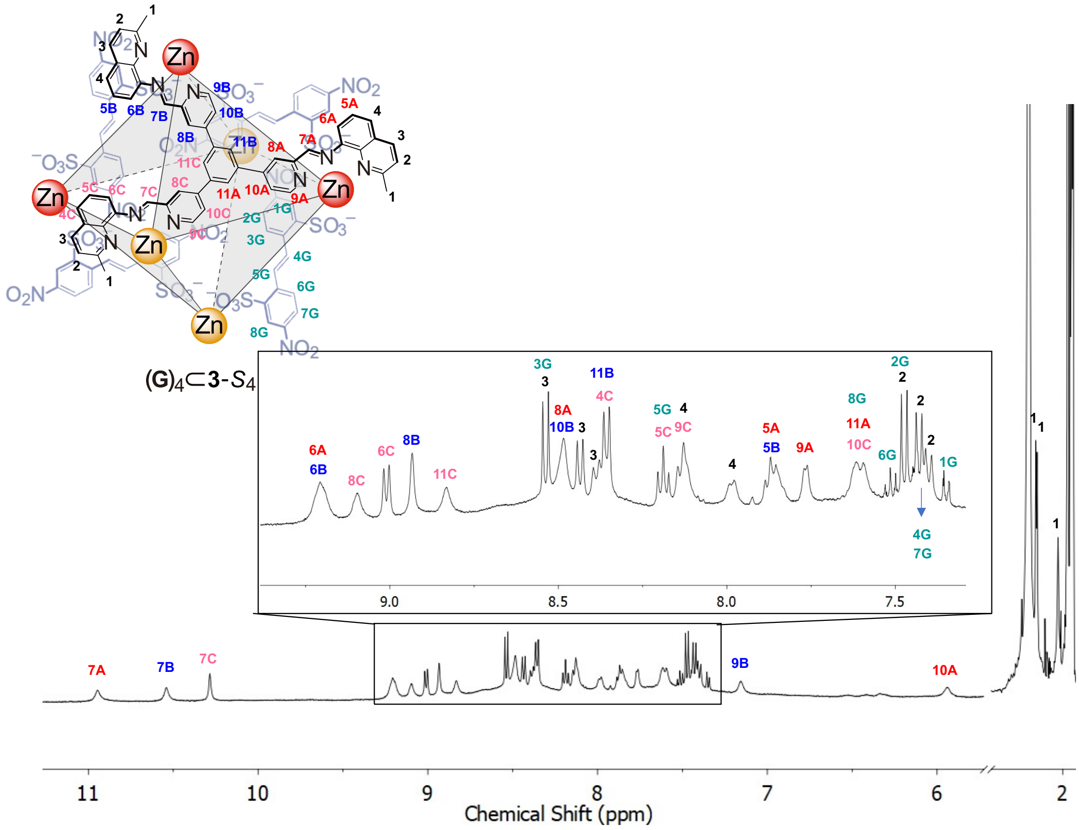

Figure S31. ^{1}H NMR spectrum of (**G**)$_4$⊂**3**-S_4 (500 MHz, CD_3CN, 298 K) with assignments of protons shown.

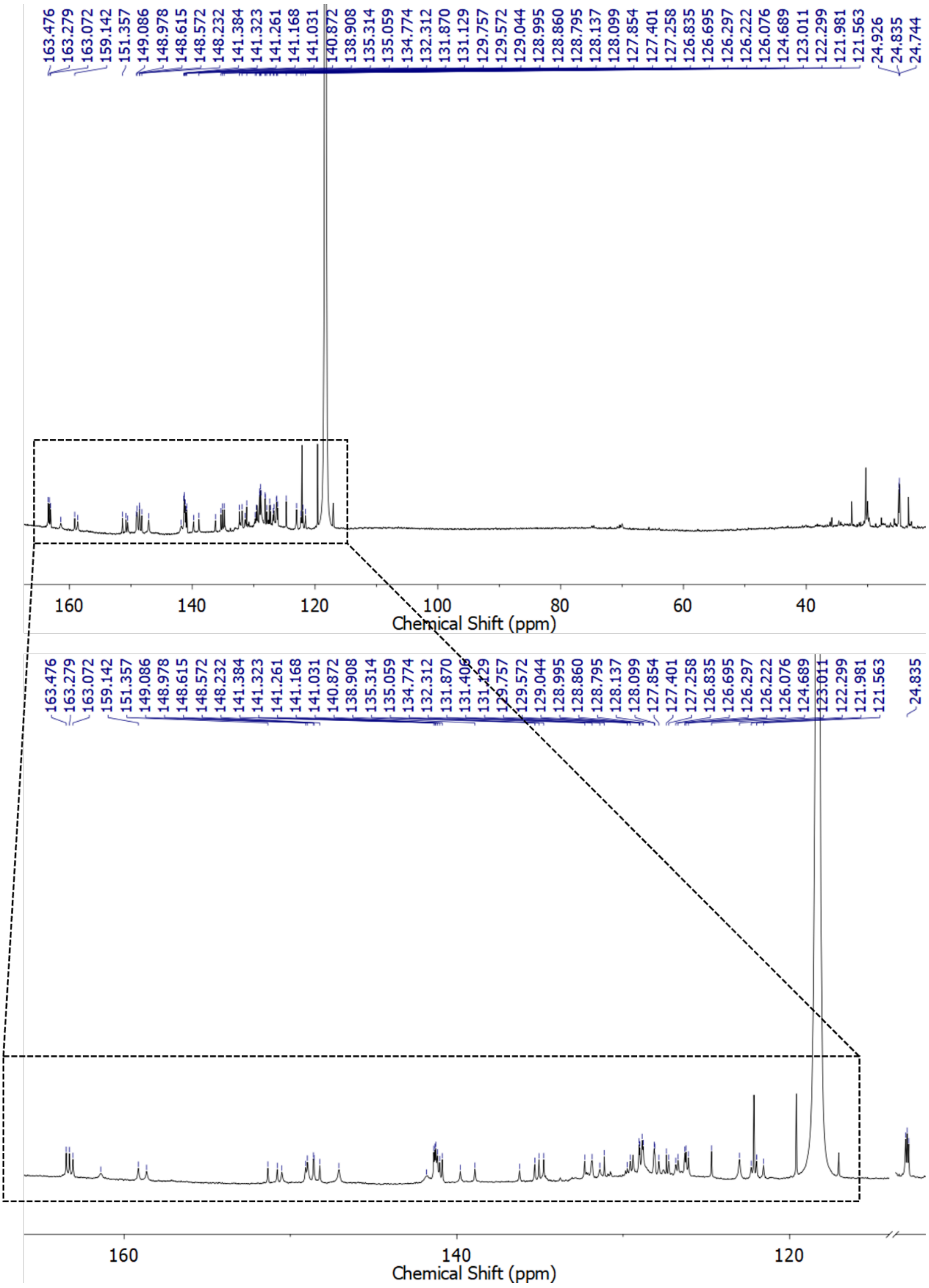

Figure S32. ^{13}C NMR spectrum of (**G**)$_4$⊂**3**-S_4 (125 MHz, CD_3CN, 298 K).

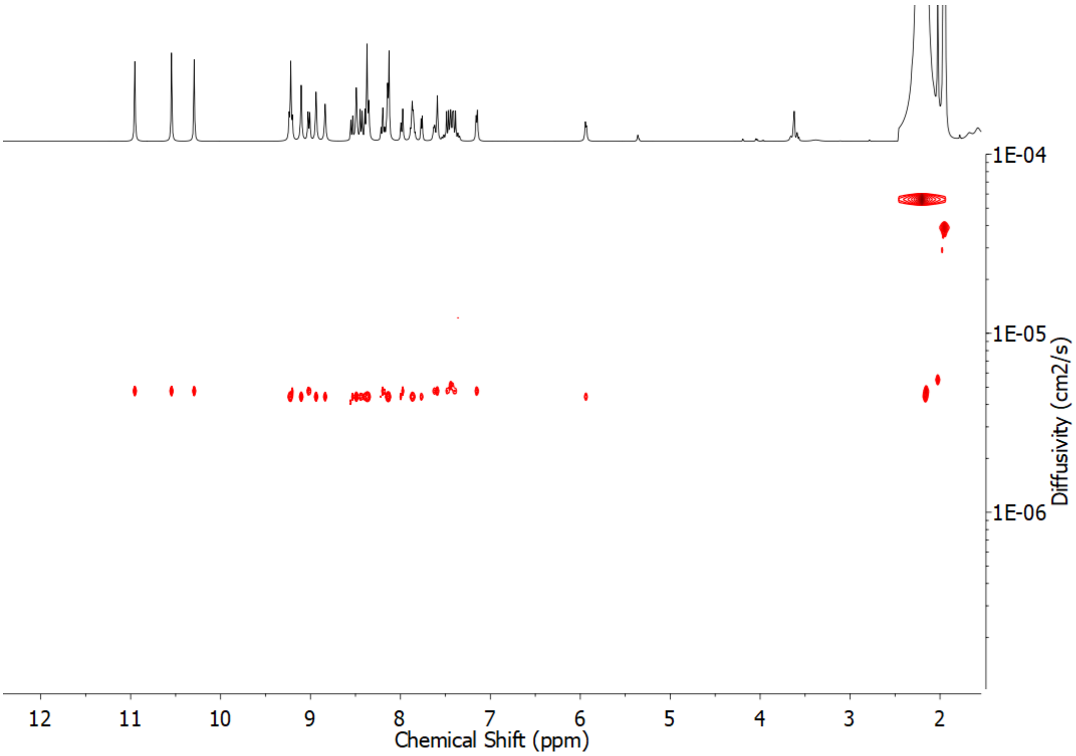

Figure S33. ^{1}H DOSY spectrum of (**G**)$_4$⊂**3**-*S*$_4$ (400 MHz, CD_3CN, 298 K). The diffusion coefficient for both diastereomers in CD_3CN was measured to be 4.53 × 10^{-6} cm^2/s.

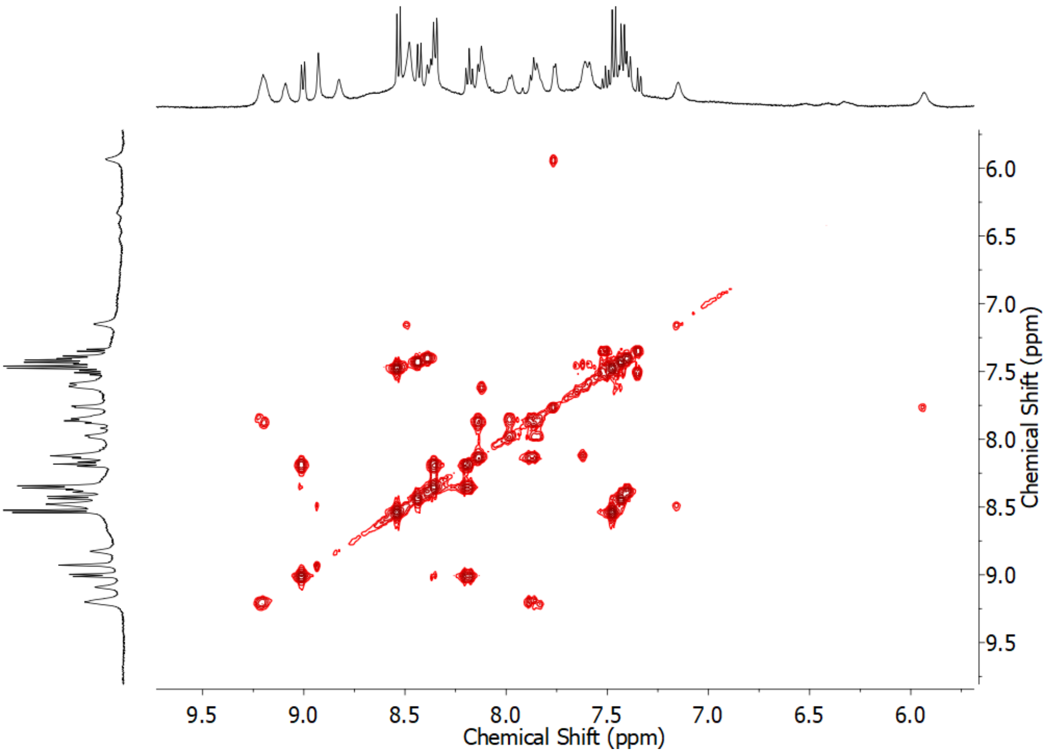

Figure S34. ^{1}H–^{1}H COSY NMR spectrum of (**G**)$_4$⊂**3**-*S*$_4$ (500 MHz, CD_3CN, 298 K).

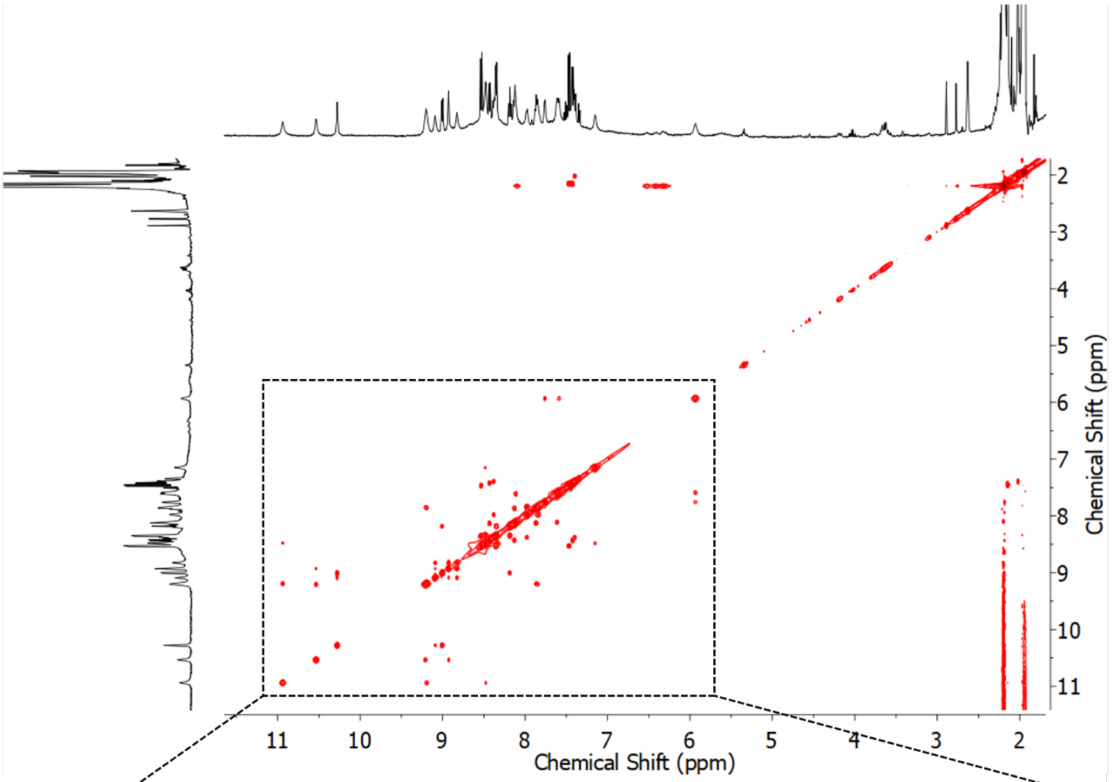

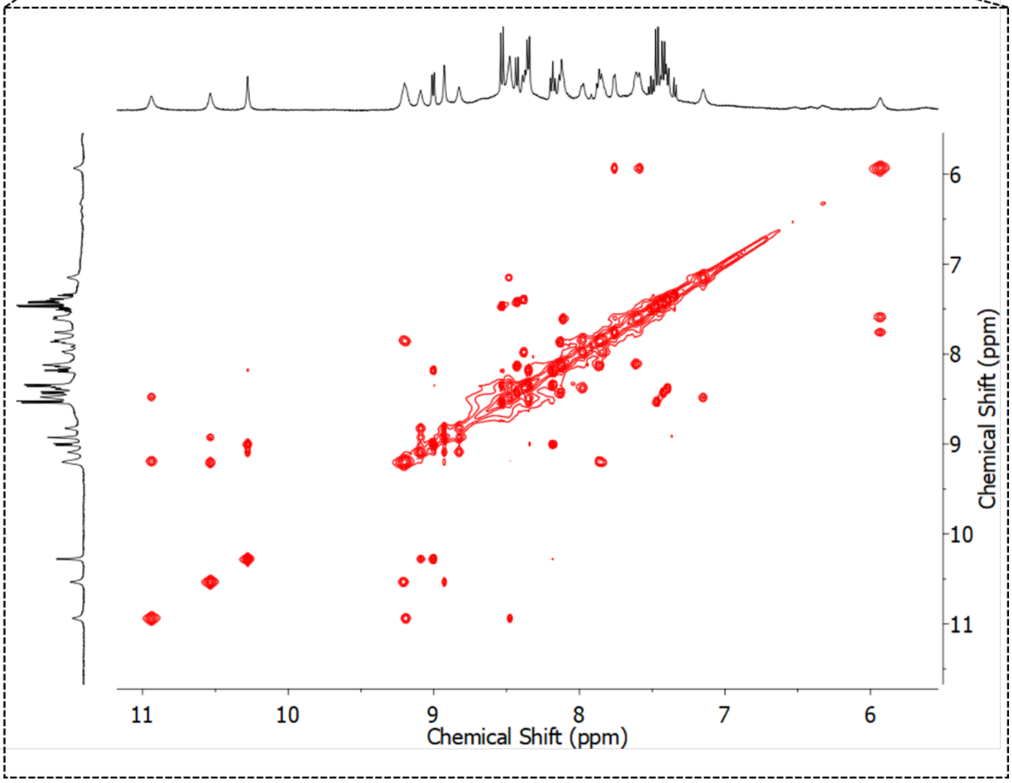

Figure S35. ^1H–^1H NOESY NMR spectrum of (**G**)$_4$⊂**3**-S_4 (500 MHz, CD_3CN, 298 K).

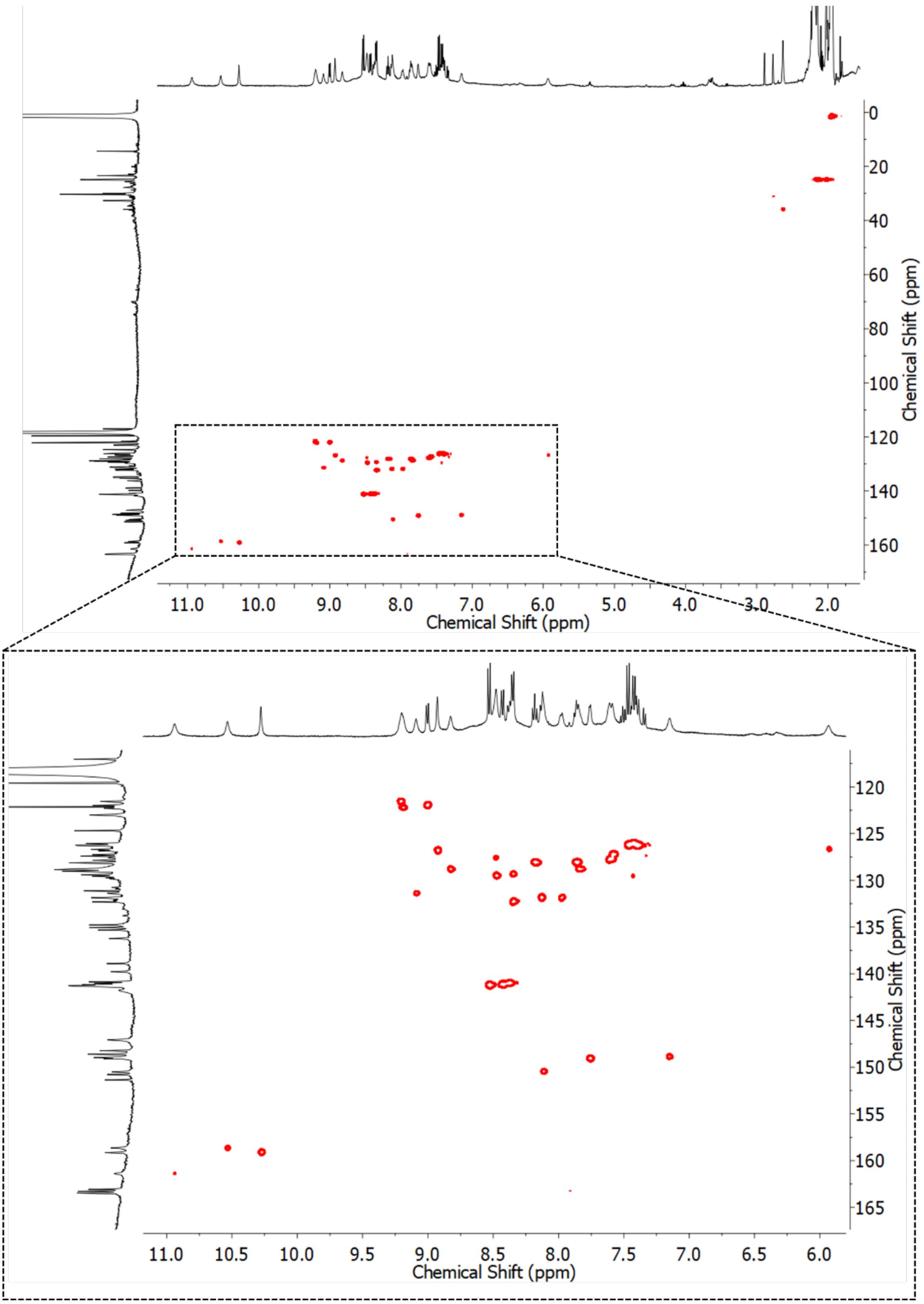

Figure S36. ^{1}H–^{13}C HSQC NMR spectrum of (**G**)$_4$⊂**3**-S_4 (500 MHz, CD_3CN, 298 K).

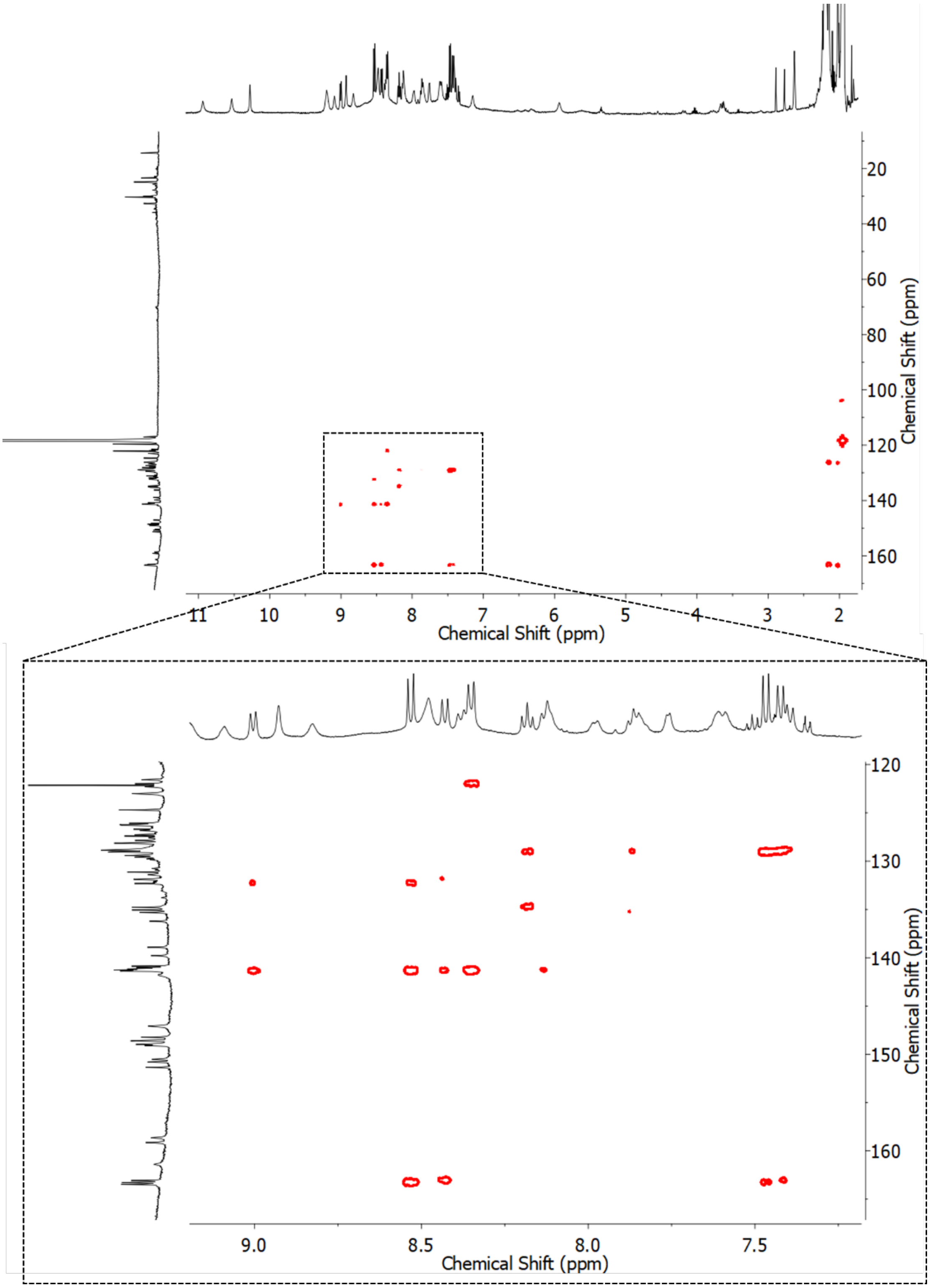

Figure S37. ^{1}H–^{13}C HMBC NMR spectrum of (**G**)$_4$⊂**3**-*S*$_4$ (500 MHz, CD_3CN, 298 K).

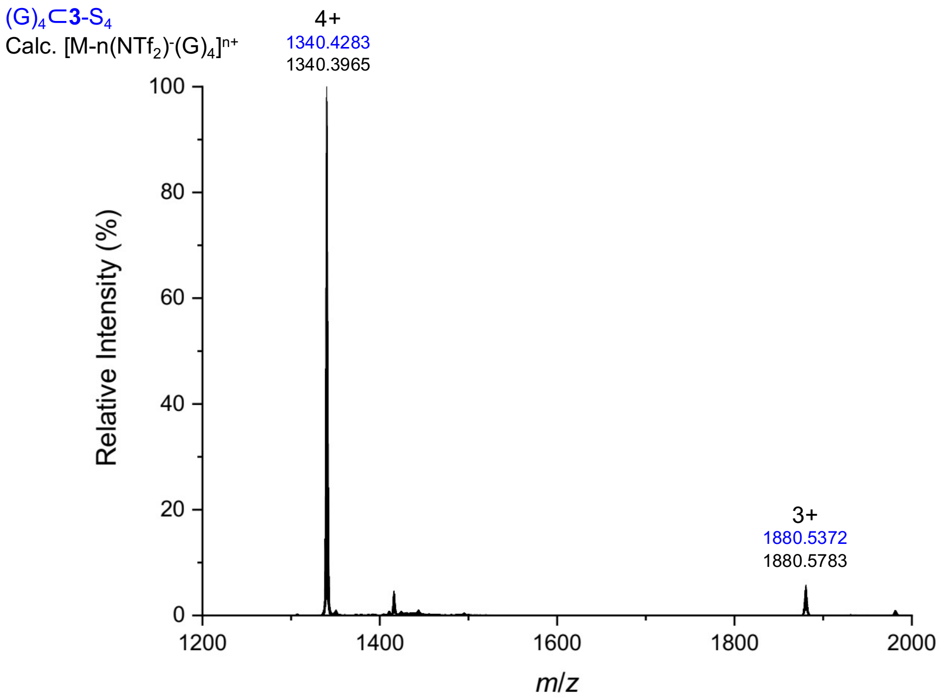

Figure S38. HR–ESI–MS spectrum of (**G**)$_4$⊂**3**-*S*$_4$ in CH_3CN. Experimental (blue) and calculated (black) peaks. **G** = 4,4'-dinitrostilbene-2,2'-disulfonate.

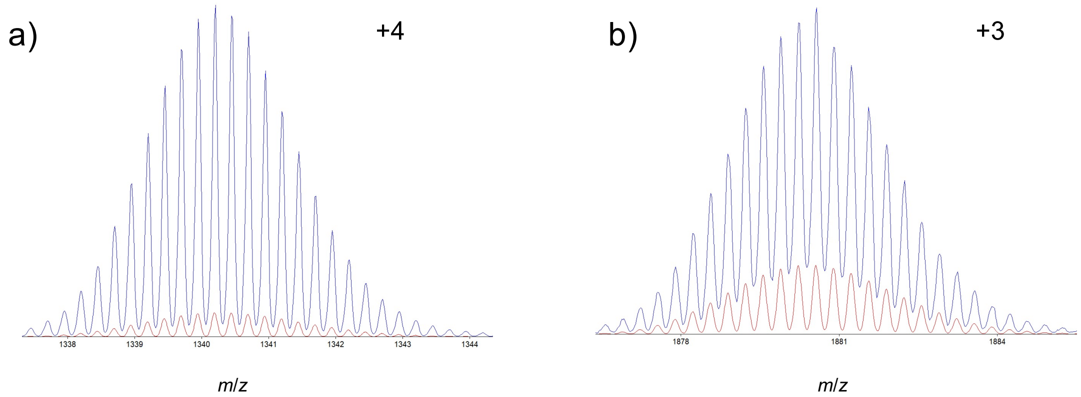

Figure S39. HR–ESI–MS spectra of (**G**)$_4$⊂**3**-*S*$_4$ in CH_3CN. Experimental (blue) and calculated (red) peaks for a) [**2**·(**G**)$_4$]$^{4+}$; b) [**2**·(NTf_2)(**G**)$_4$]$^{3+}$. **G** = 4,4'-dinitrostilbene-2,2'-disulfonate.

4.3.2 Investigation of whether dilution of 2 is a factor in conversion of 2 to (G)$_4$⊂3.

We monitored the reaction at ligand concentration of 5 or 2.5 mM at r.t. or 70 °C (Figures S40-S42). The dilution of the ligand accelerated the conversion of **2** into (**G**)$_4$⊂**3** because of the poor solubility of **G** and (**G**)$_4$⊂**3**.

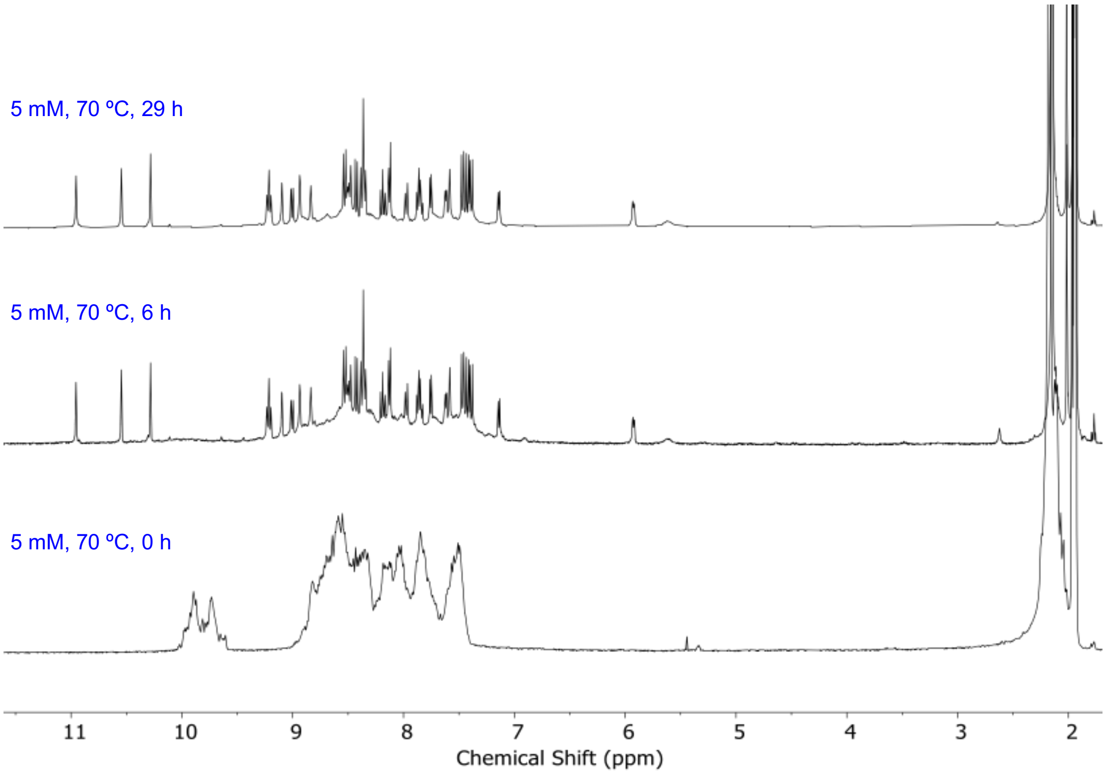

Figure S40. ^{1}H NMR spectra monitoring the conversion of **2** to (**G**)$_4$⊂**3** in the presence of **G** over different reaction times (400 MHz, 298 K, CD_3CN, 5 mM ligand concentration at 70 °C).

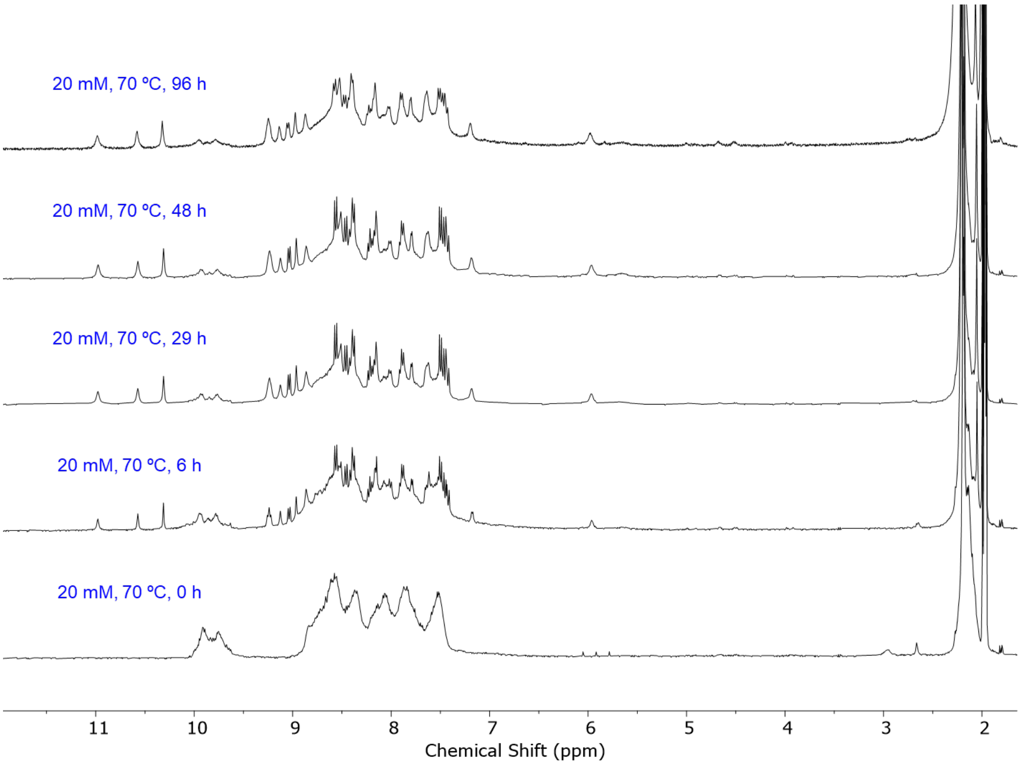

Figure S41. ^{1}H NMR spectra monitoring the conversion of **2** to (**G**)$_4$⊂**3** in the presence of **G** over different reaction times (400 MHz, 298 K, CD_3CN, 20 mM ligand concentration at 70 °C).

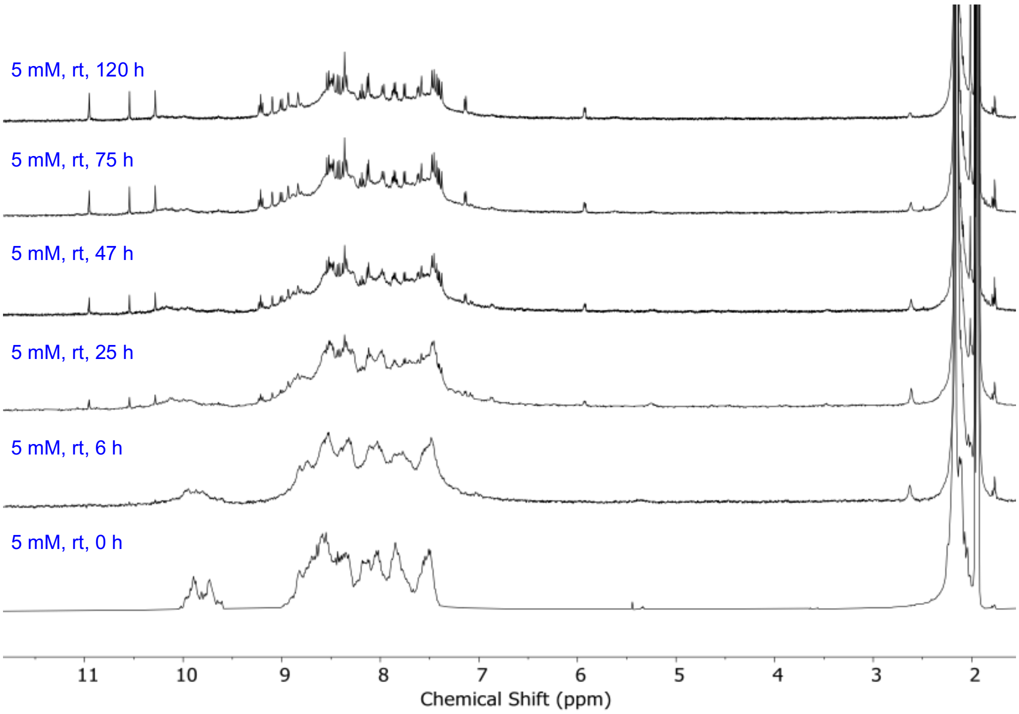

Figure S42. ^{1}H NMR spectra monitoring the conversion of **2** to (**G**)$_4$⊂**3** in the presence of **G** over different reaction times (400 MHz, 298 K, CD_3CN, 5 mM ligand concentration at room temperature).

4.3.3 Crystallographic analysis of interactions between $Zn^{II}_6L_4$ host and guests.

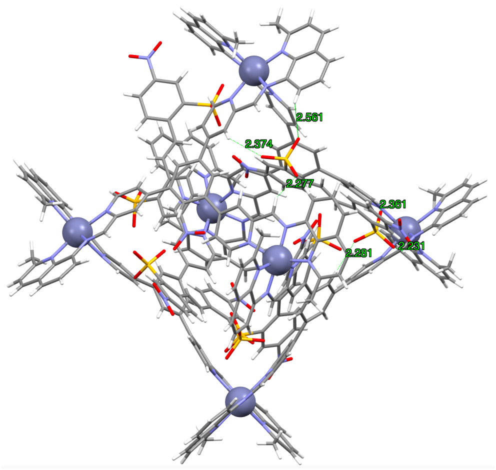

Figure S43. Distances between hydrogen atoms on the ligands of cage and oxygen atoms on the sulfonate groups of guests.

The driving force for the binding of negatively-charged guests by positively-charged hosts is primarily electrostatic attraction. The conversion from $Zn^{II}_9L_6$ to $Zn^{II}_6L_4$ was realized not only due to electrostatic interactions, but also to van der Waals interactions and hydrogen bonding between the cage and the guests.

In the crystal structure of $(\mathbf{G})_4{\subset}\mathbf{3}$, the distances between hydrogen atoms on the host ligands and oxygen atoms on the sulfonate groups of guests are 2.23 to 2.56 Å, less than the 2.6 Å sum of the van der Waals radii, which suggests the existence of hydrogen bonding between them.[2] Evidence for the presence of these interactions in solution is given by NMR spectroscopy, for example by the changes in chemical shift of the imine protons following guest addition (Figures S46, S48, S54 and S56).

5. NMR Titrations of Guests with Cage 1

5.1 Job Plots of NMR Titrations

5.1.1 Job Plot of Tetrakis(4-chlorophenyl)borate as a Guest for 1

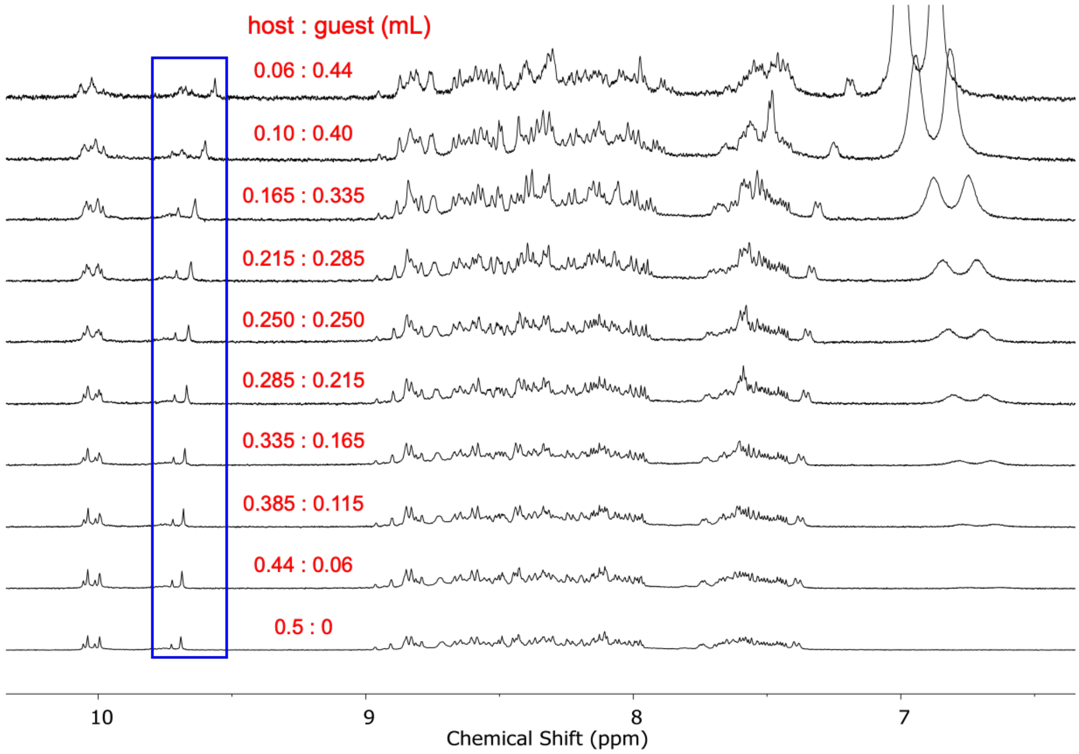

Figure S44. ^{1}H NMR titration of **1** with KB(*p*-$C_6H_4Cl)_4$ (400 MHz, 298 K, CD_3CN, 1.0 mM in both **1** and KB(*p*-$C_6H_4Cl)_4$).

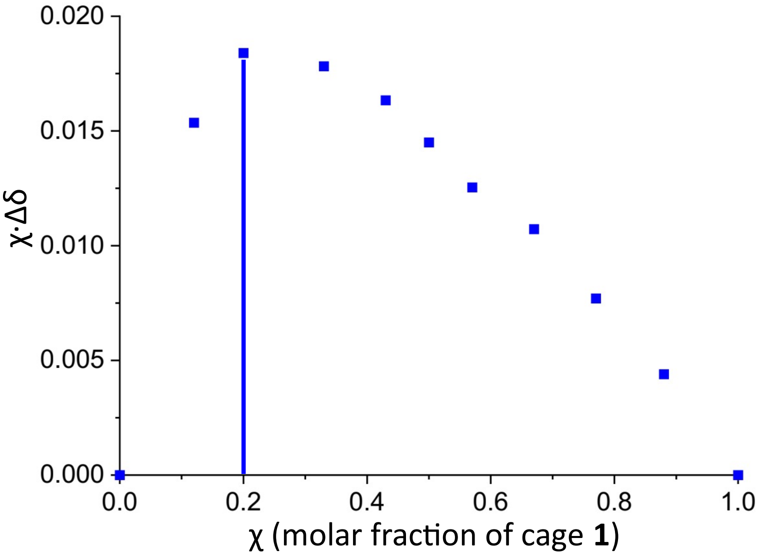

Figure S45. Job plot revealed a 1:4 H:G ratio for **1**·(B(*p*-$C_6H_4Cl)_4^-)_4$.

5.1.2 Job Plot of Periodate as a Guest for 1

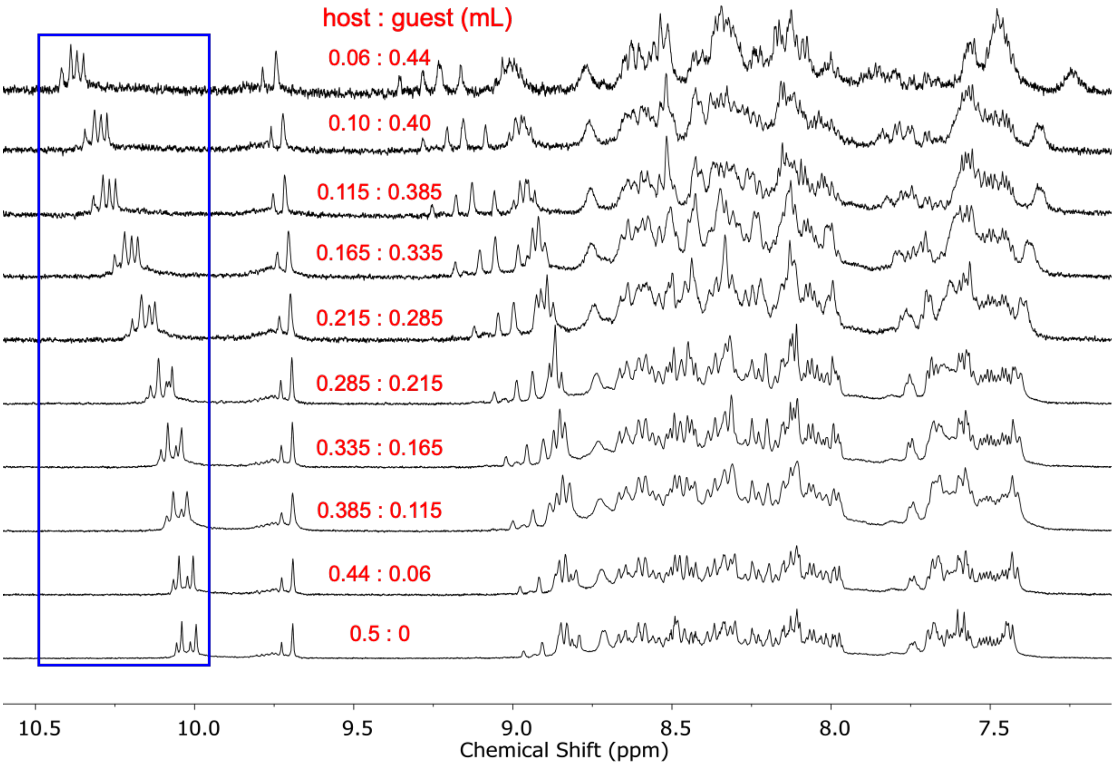

Figure S46. ^{1}H NMR spectra of titration of **1** with $TBAIO_4$ (400 MHz, 298 K, CD_3CN, 1.0 mM in both **1** and $TBAIO_4$).

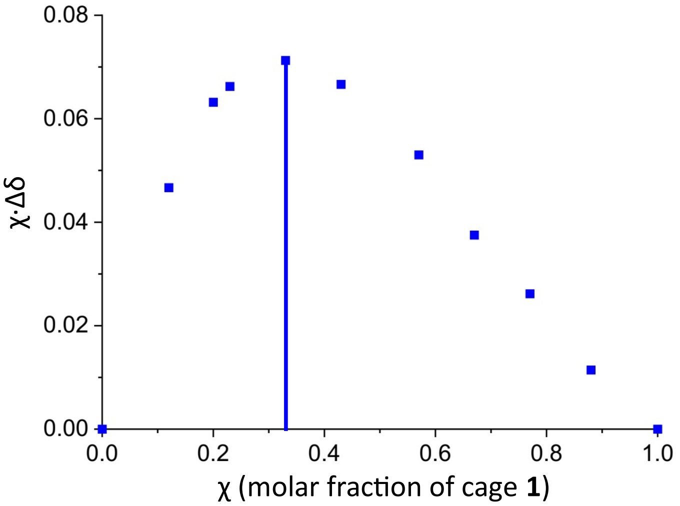

Figure S47. Job plot revealed a 1:2 H:G ratio for **1**$\cdot(IO_4^-)_2$

5.1.3 Job Plot of Perrhenate as a Guest for 1

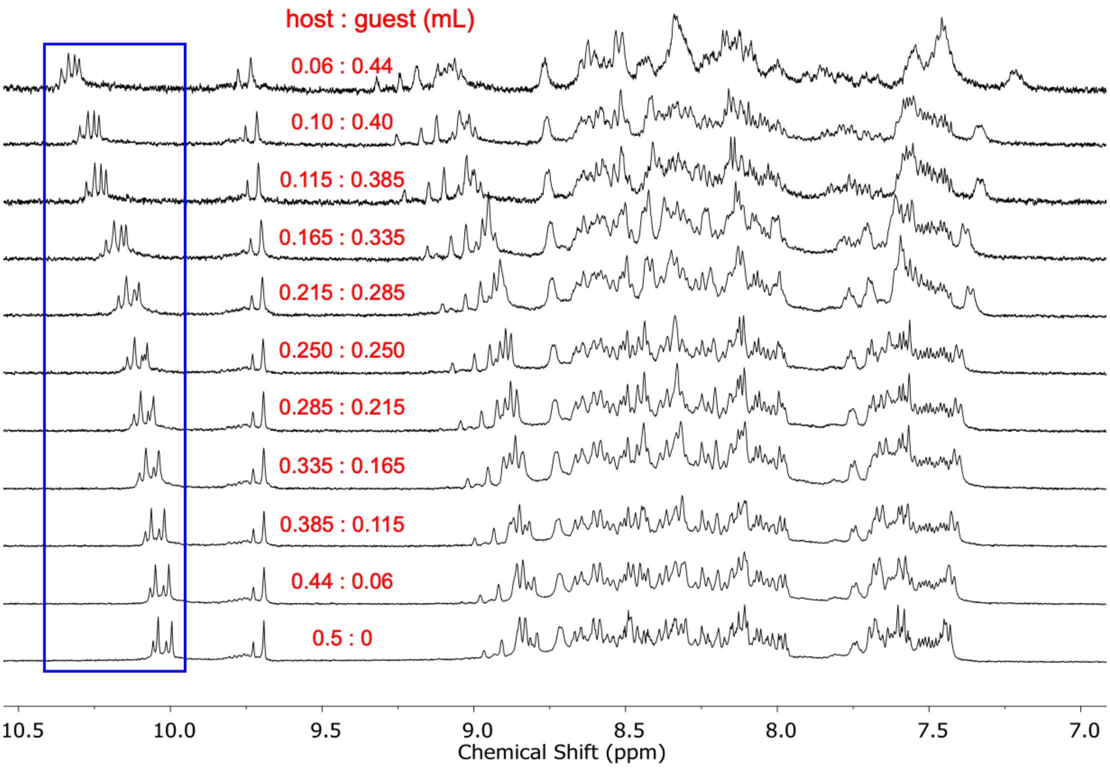

Figure S48. ^{1}H NMR spectra of titration of **1** with TBAReO$_4$ (400 MHz, 298 K, CD$_3$CN, 1.0 mM in both **1** and TBAReO$_4$).

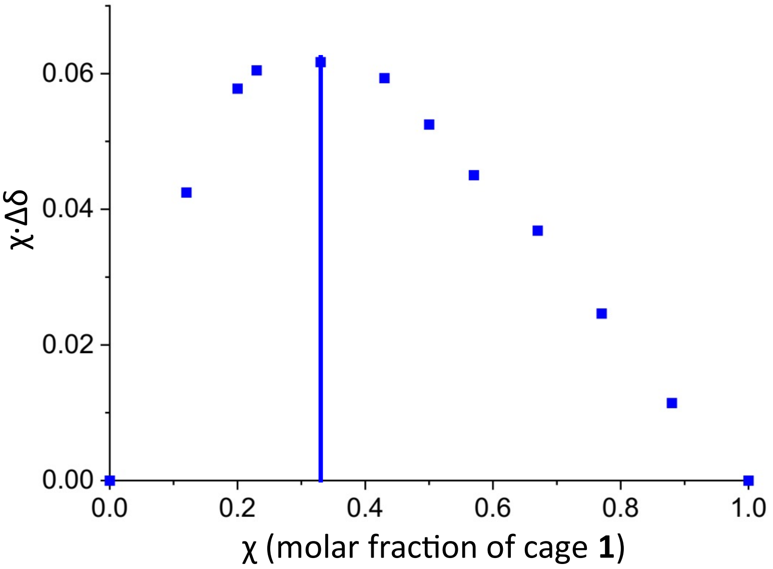

Figure S49. Job plot revealed a 1:2 H:G ratio for **1**·$(ReO_4^-)_2$.

5.2 Hill Equation Analyses of NMR Titrations

The binding behavior of cage **1** was studied by ^{1}H NMR titration of $B(p\text{-}C_6H_4Cl)_4^-$, $B(p\text{-}C_6H_4F)_4^-$, ReO_4^-, and IO_4^- into solutions of **1**·$(NTf_2)_{12}$, respectively. Titration experiments were carried out in CD_3CN. The CD_3CN solution cage **1** (1.0 mM) was prepared. The CD_3CN solution of both corresponding guest (25.0 mM) and cage **1** (1.0 mM) were also prepared and used for the titrations. The ^{1}H NMR titration were performed by adding aliquots of guest mixed with cage **1** stock solution to 500 μL of cage **1** stock solution. It was found that guests $B(p\text{-}C_6H_4Cl)_4^-$, $B(p\text{-}C_6H_4F)_4^-$, ReO_4^-, and IO_4^- were bound by **1** in fast-exchange on the NMR time scale at 25 °C.

The binding constant (K_a) and coefficient value (n) were calculated using the Hill equation.[3]

$$\theta = \frac{[HG_n]}{[HG_n] + [H]} \tag{S1}$$

$$\log\frac{\theta}{1 - \theta} = n\log[G] + n\log K_a \tag{S2}$$

Where θ is the fraction of binding sites occupied by the guest, [G] is the guest concentration, n is the Hill coefficient describing cooperativity, and K_a is the apparent association constant.

In the Hill equation, the value of θ was obtained using Equation S1. For the fast-exchange bound guests: $B(p\text{-}C_6H_4Cl)_4^-$, $B(p\text{-}C_6H_4F)_4^-$, ReO_4^-, and IO_4^-, the value of θ was obtained by using $\Delta\delta_{observed}$ as compared against the maximum change of chemical shift $\Delta\delta$ of corresponding protons in ^{1}H NMR spectra.

Cooperativity is quantified by the Hill coefficient n, where n>1 indicates positively cooperative binding, n<1 indicates negatively cooperative binding, and n=1 indicates noncooperative binding.

5.2.1 Hill Equation Analysis of Tetrakis(4-chlorophenyl)borate as a Guest for 1

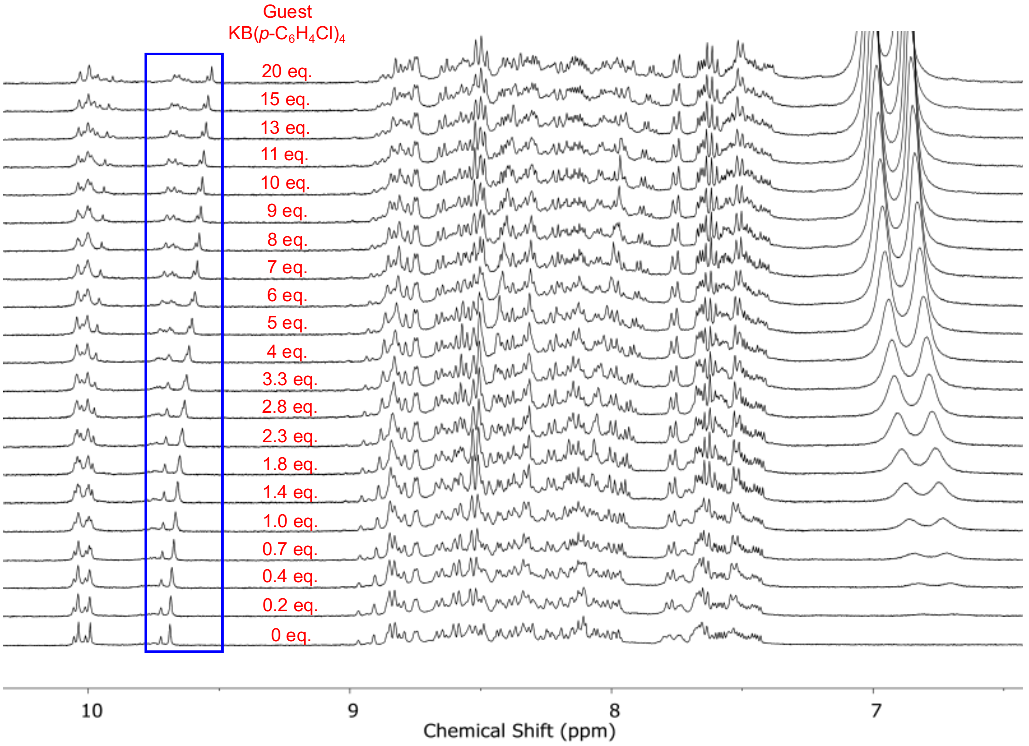

Figure S50. ^{1}H NMR spectra (400 MHz, 298 K, CD_3CN) **1** (c = 1.0 mM) upon addition of 0 – 20 equiv of KB(*p*-C_6H_4Cl)$_4$. Guest equivalents with respect to the complete host mixture are given in red.

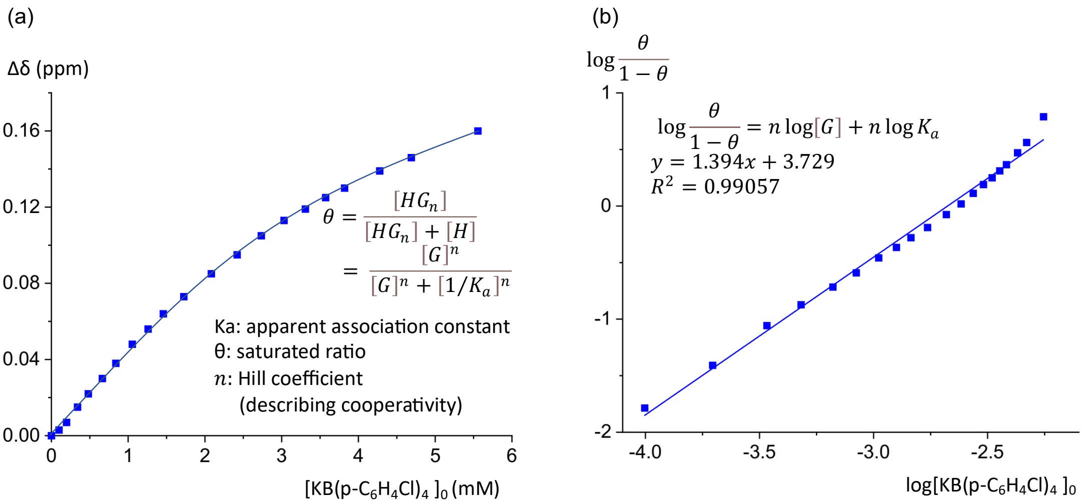

Figure S51. Titration curves of **1** *vs* KB(*p*-C_6H_4Cl)$_4$ and Hill function analysis. (a) Δδ *vs* [KB(*p*-C_6H_4Cl)$_4$]$_0$ and (b) θ *vs* log[KB(*p*-C_6H_4Cl)$_4$]$_0$.

5.2.2 Hill Equation Analysis of Tetrakis(4-fluorophenyl)borate as a Guest for 1

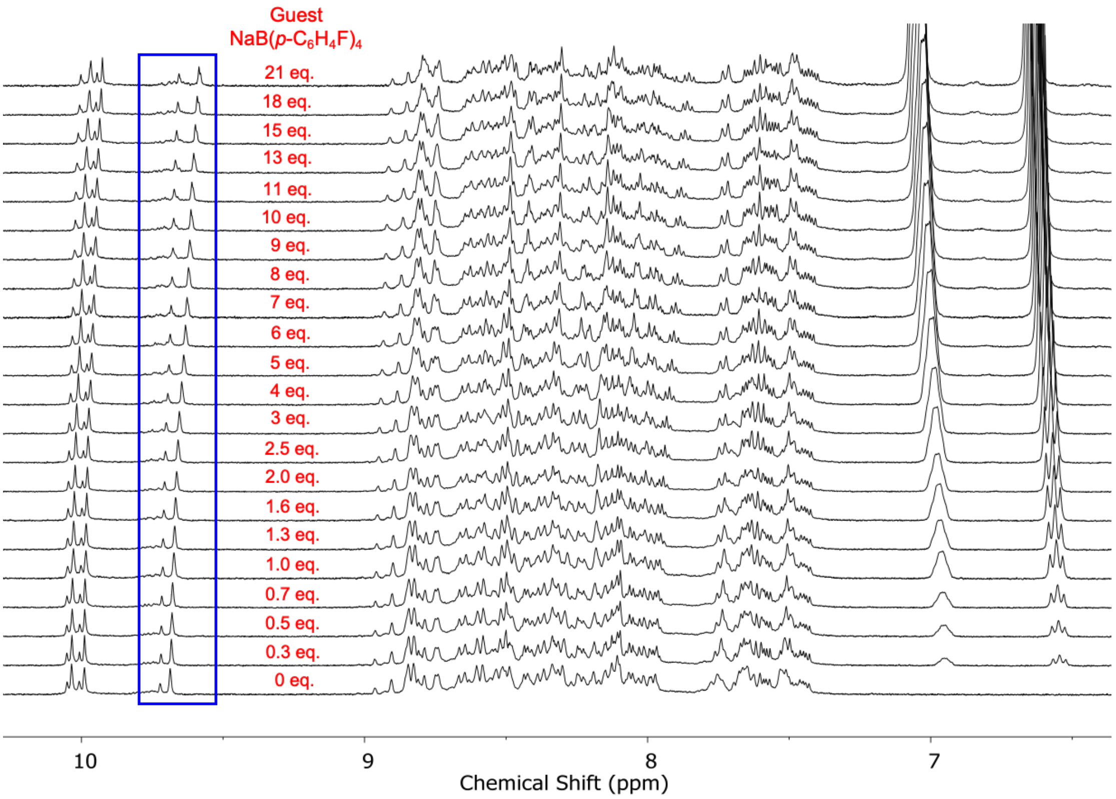

Figure S52. ^{1}H NMR spectra (400 MHz, 298 K, CD_3CN) of **1** (c = 1.0 mM) upon addition of 0 – 21 equiv of NaB(*p*-C_6H_4F)$_4$. Guest equivalents with respect to the complete host mixture are given in red.

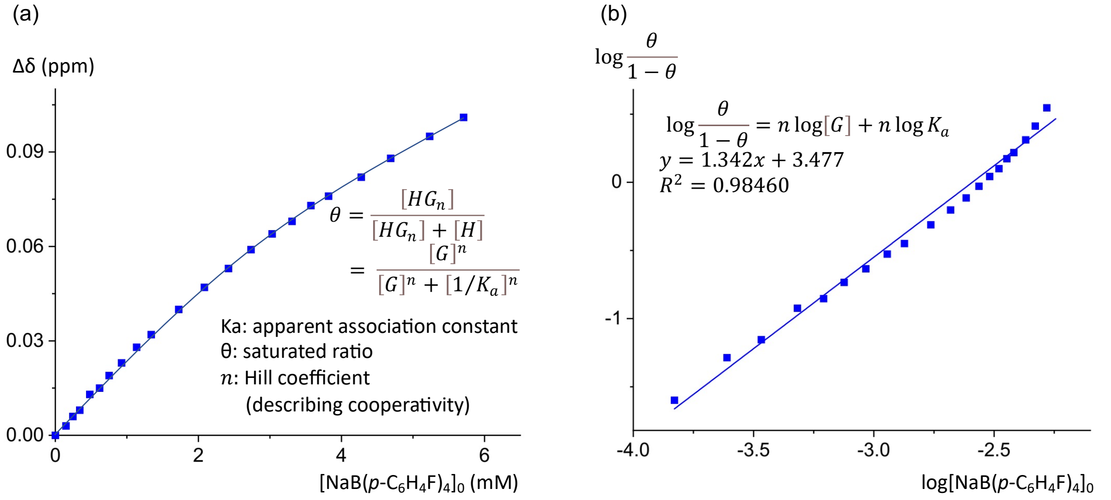

Figure S53. Titration curves of **1** *vs* NaB(*p*-C_6H_4F)$_4$ and Hill function analysis. (a) Δδ *vs* [NaB(*p*-C_6H_4F)$_4$]$_0$ and (b) θ *vs* log[NaB(*p*-C_6H_4F)$_4$]$_0$.

5.2.3 Hill Equation Analysis of Perrhenate as a Guest for 1

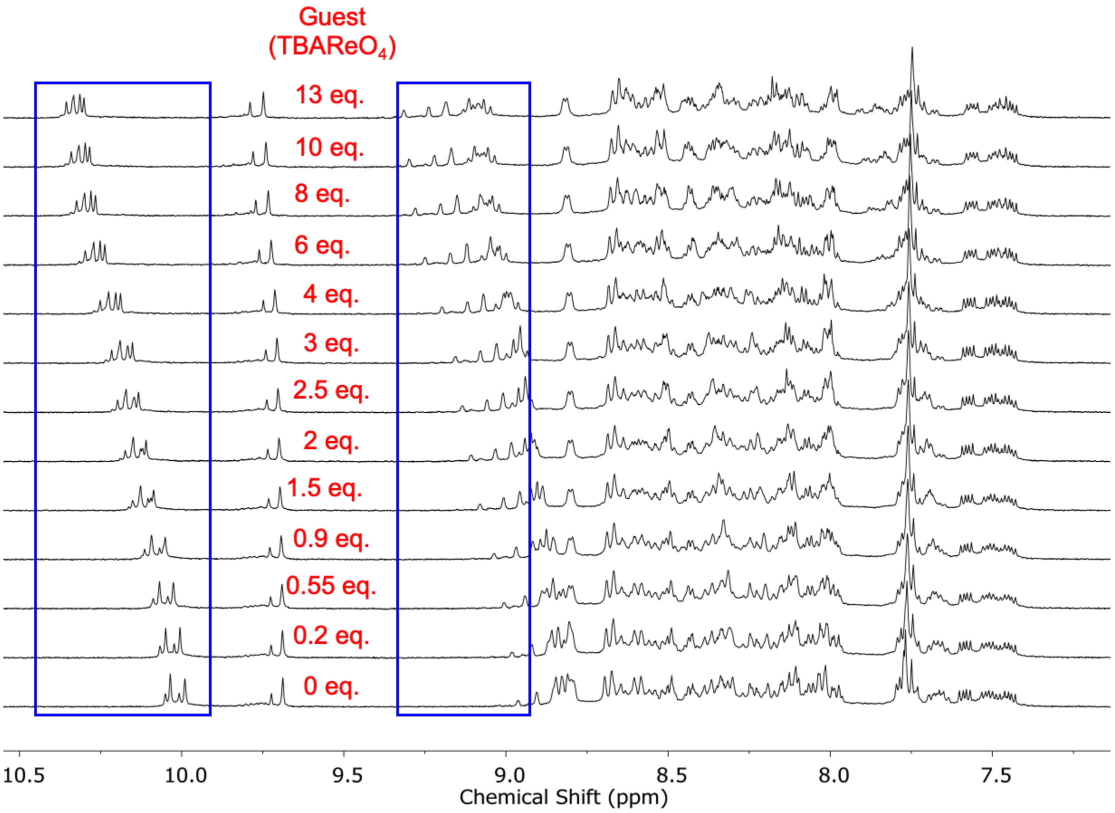

Figure S54. ^{1}H NMR spectra (400 MHz, 298 K, CD_3CN) of **1** (c = 1.0 mM) upon addition of 0 – 13 equiv of $TBAReO_4$. Guest equivalents with respect to the complete host mixture are given in red.

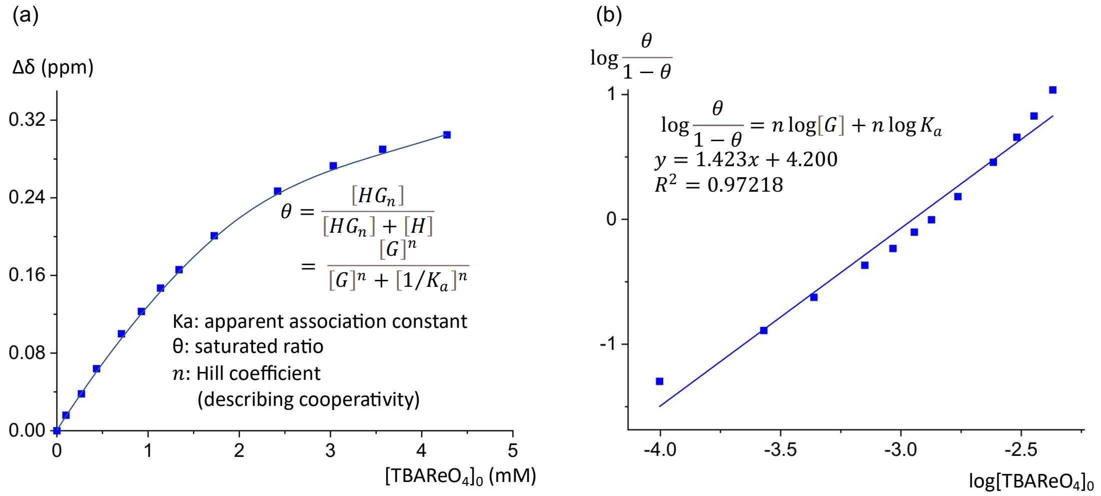

Figure S55. Titration curves of **1** *vs* $TBAReO_4$ and Hill function analysis. (a) Δδ *vs* $[TBAReO_4]_0$ and (b) θ *vs* $\log[TBAReO_4]_0$.

5.2.4 Hill Equation Analysis of Periodate as a Guest for 1

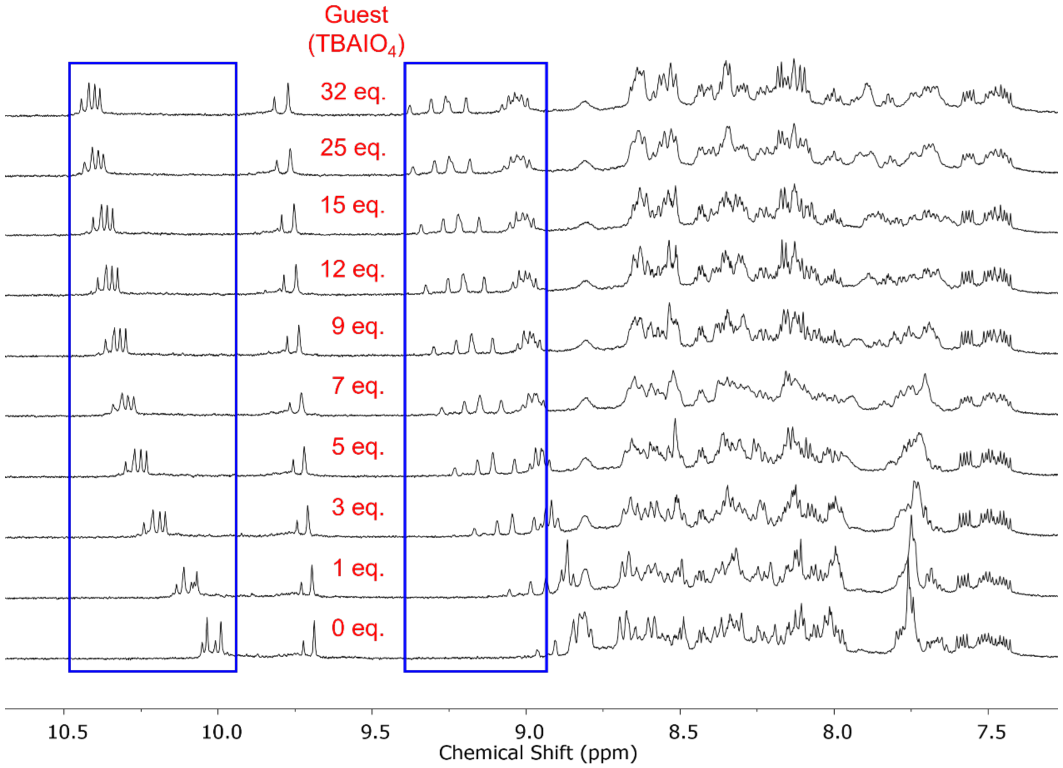

Figure S56. ^{1}H NMR spectra (400 MHz, 298 K, CD_3CN) of **1** (c = 1.0 mM) upon addition of 0 – 32 equiv of $TBAIO_4$. Guest equivalents with respect to the complete host mixture are given in red.

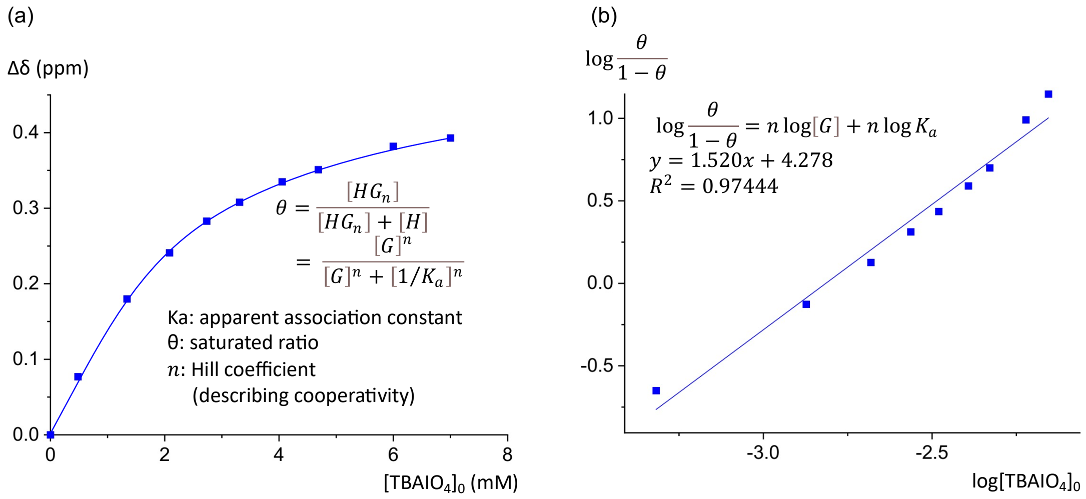

Figure S57. Titration curves of **1** *vs* $TBAIO_4$ and Hill function analysis. (a) Δδ *vs* $[TBAIO_4]_0$ and (b) θ *vs* $\log[TBAIO_4]_0$.

5.2.5 Titration Experiment of Pyrene as a Guest for 1

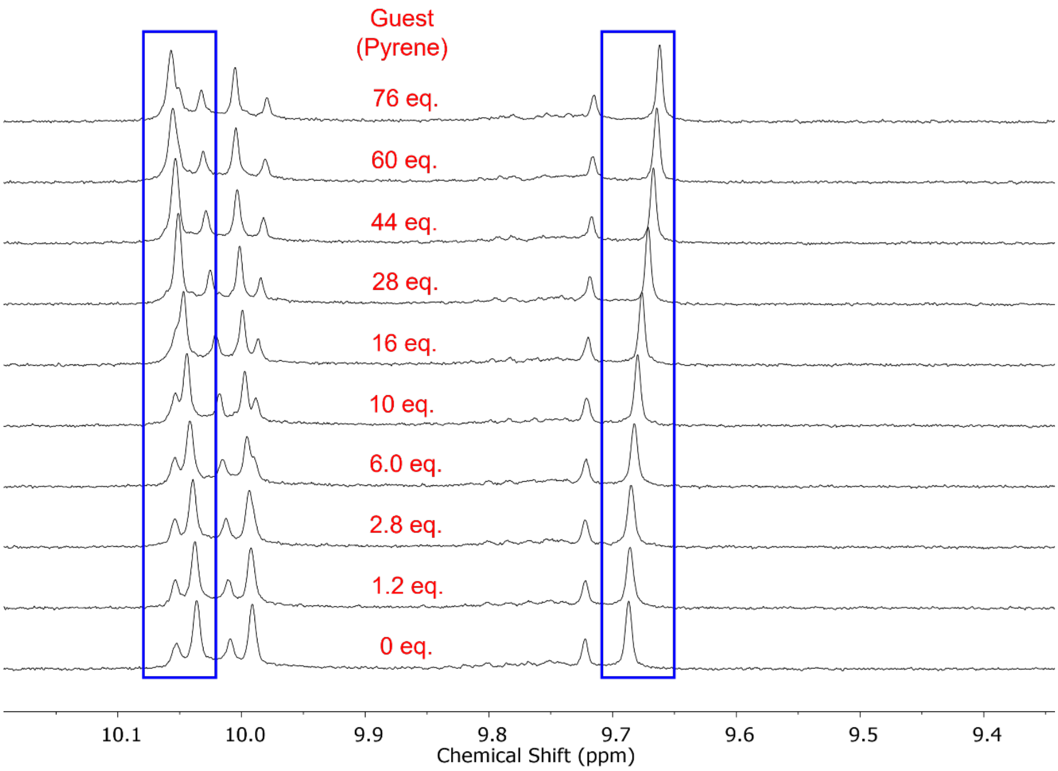

Figure S58. ^{1}H NMR spectra (400 MHz, 298 K, CD_3CN) of **1** (c = 1.0 mM) upon addition of 0 – 76 equiv of pyrene. Guest equivalents with respect to the complete host mixture are given in red. The precipitate was observed in the process of titration.

6 Host–Guest Studies Using Cage 1

6.1 Binding Guests for Cage 1

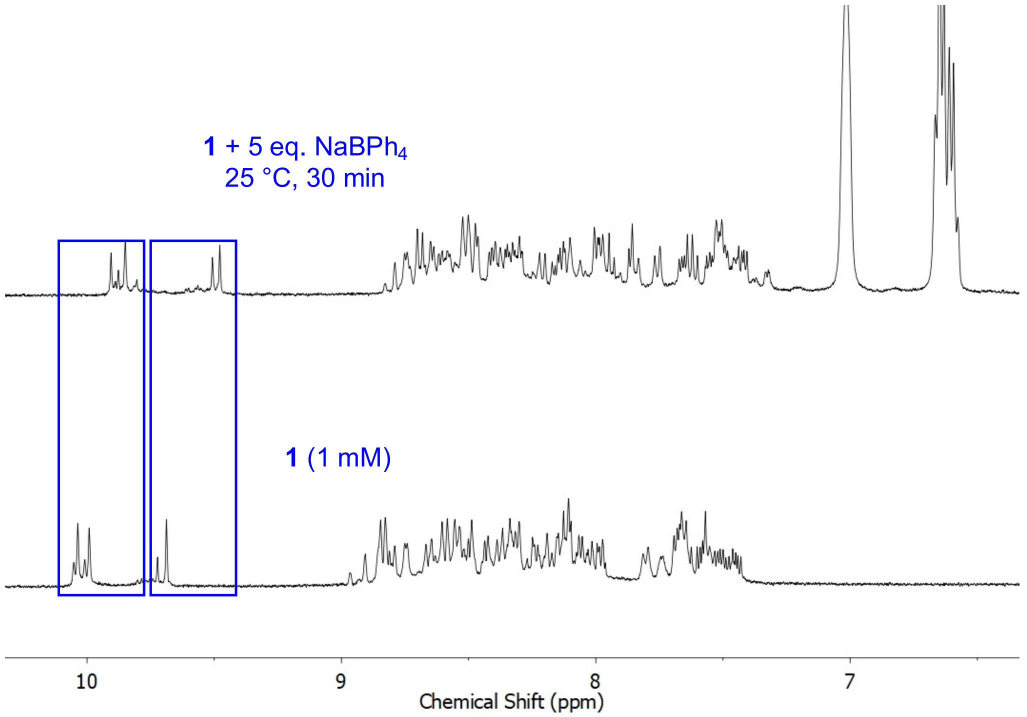

Figure S59. ^{1}H NMR spectra (400 MHz, 298 K, CD_3CN) of host–guest complex of **1** (top) compared to the free cage **1** (bottom).

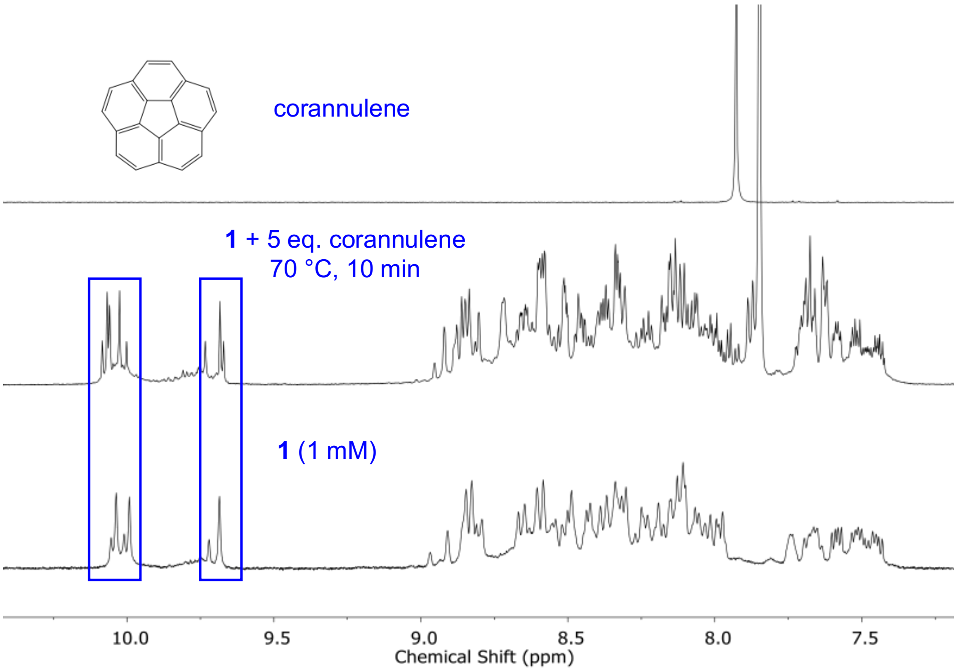

Figure S60. ^{1}H NMR spectra (400 MHz, 298 K, CD_3CN) of host–guest complex of **1** (middle) compared to the free cage **1** (bottom) and the guest corannulene (top).

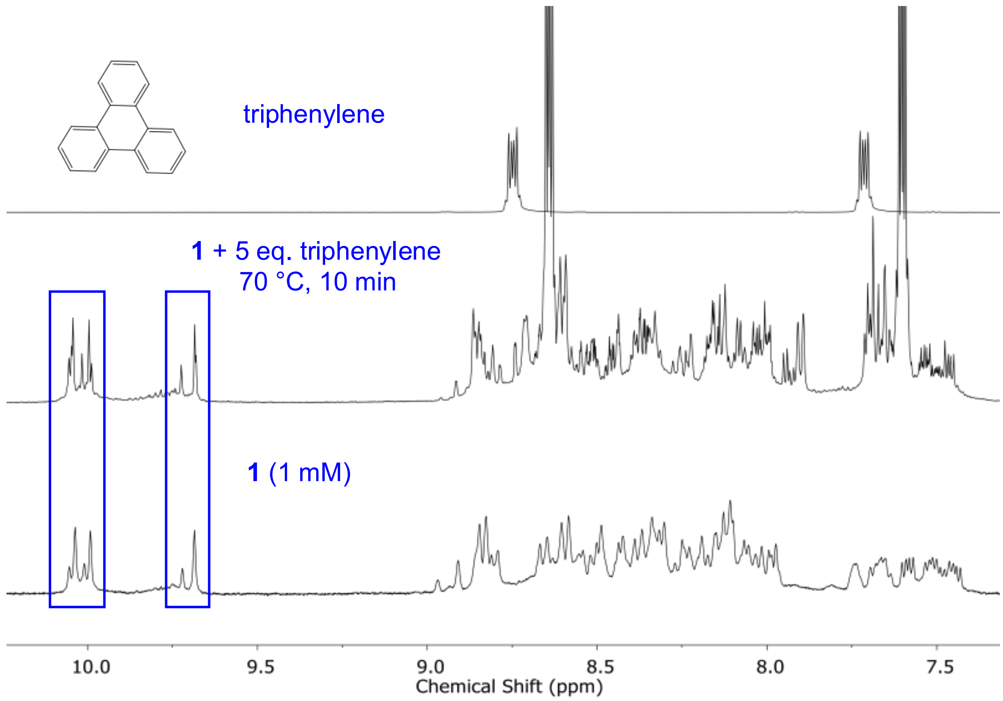

Figure S61. ^{1}H NMR spectra (400 MHz, 298 K, CD_3CN) of host–guest complex of **1** (middle) compared to the free cage **1** (bottom) and the guest triphenylene (top).

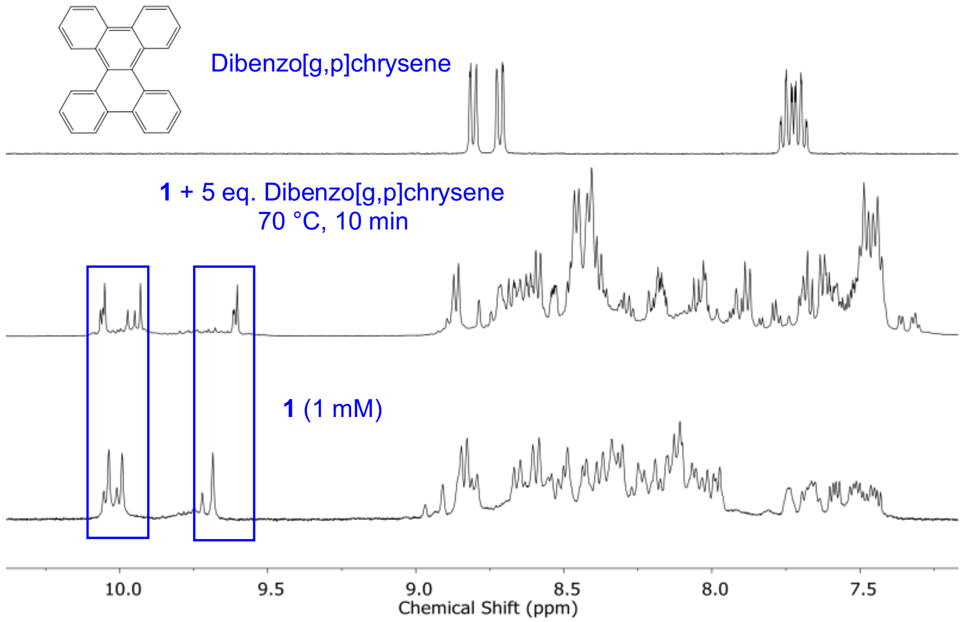

Figure S62. ^{1}H NMR spectra (400 MHz, 298 K, CD_3CN) of host–guest complex of **1** (middle) compared to the free cage **1** (bottom) and the guest dibenzo[g,p]chrysene (top).

6.2 Non–Binding Guests for Cage 1

Prospective guests (2.5 µmol, 5 equiv) and cage **1** (0.5 µmol, 1.0 equiv) were combined in CD_3CN (0.5 mL) in NMR tubes. The reaction mixtures were heated at 70 °C for 1 hour. After cooling to room temperature, ^1H NMR spectra were measured. No changes in chemical shift were observed for the following species:

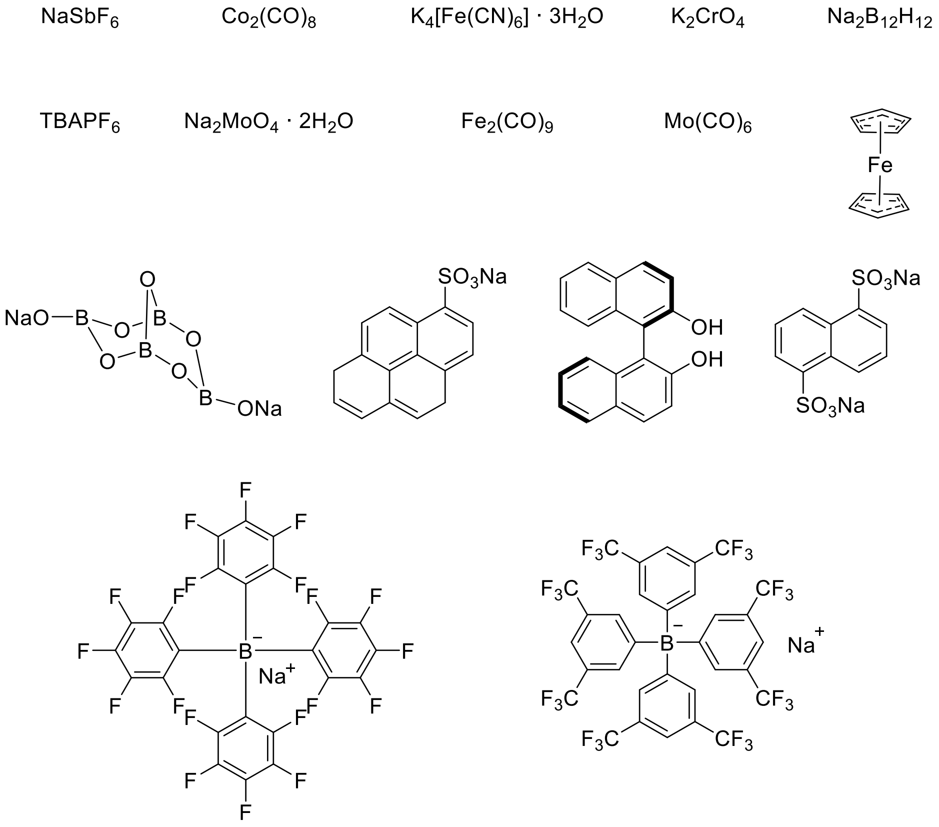

Figure S63. Non–binding prospective guests.

7 Contrast of Octahedra with Low or High symmetry

Table S1. Comparison of different C_2-symmetric vertices that produce octahedral cages.

Entry	Cage vertex/metal	Symmetry	Entry	Cage vertex/metal	Symmetry
1	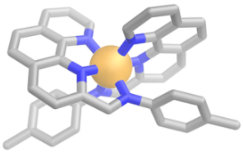 Zn^{II}	T	5	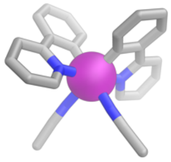 Ir^{III}	T
2	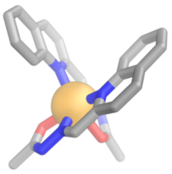 Zn^{II}	T	6	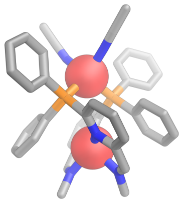 Cu^{I}	T
3	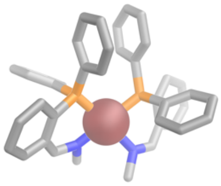 Pt^{II}	T	7	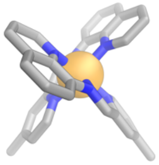 Zn^{II}	S_4, D_2
4	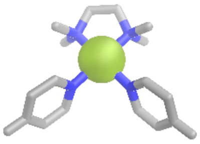 Pd^{II}	T_d			

Literature references: entry 1 (*Angew. Chem., Int. Ed.* **2016**, *55*, 7958–7962); entry 2 (*Angew., Chem. Int. Ed*. **2008**, *47*, 877–881); entry 3 (*Angew. Chem., Int. Ed*. **2022**, doi/10.1002/anie.202214577.); entry 4 (*Nat. Chem*. **2020**, *12*, 574–578); entry 5 (*J. Am. Chem. Soc*. **2012**, *134*, 19334–19337); entry 6 (*J. Am. Chem. Soc*. **2022**, *144*, 8467−8473); entry 7 is the vertex of the octahedral cage in this work.

In this work, we introduce the use of the 8-aminoquinoline subcomponent condensing with a tritopic 2-formylpyridine to form ligands where the pyridyl moiety cannot rotate freely due to steric hindrance. The arms in the ligands of other, analogous *pseudo*-octahedra (Table S2, entries 1-6) can rotate freely without any steric hindrance. The torsional steric hindrance of our new ligand, combined with the coordinate geometry of its vertices (Table S2, entry 7) led to the formation of M_6L_4 cages with D_2 and S_4 point symmetries rather than the higher symmetry octahedra (Table S2, entries 1-6).

Table S2. Comparison of different ligands that produce octahedral cages.

Entry	Ligand	Symmetry	Entry	Ligand	Symmetry
1	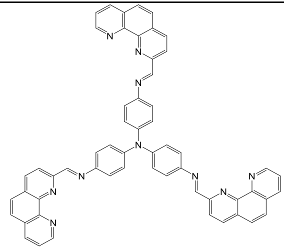	*T*	4	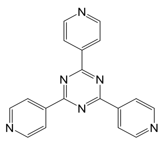	T_d
	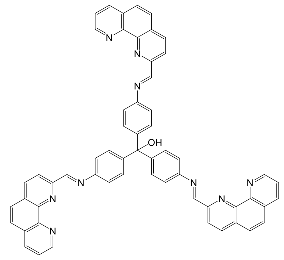	*T*	5	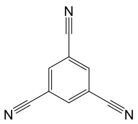	*T*
2	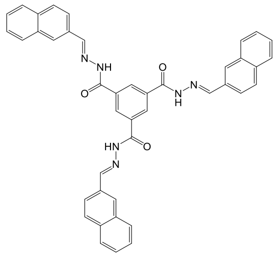	*T*	6	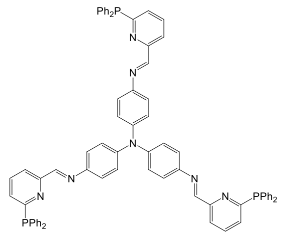	*T*
3	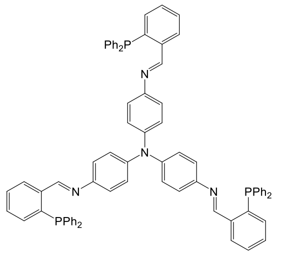	*T*	7	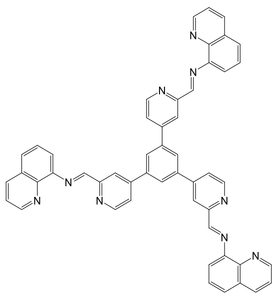	S_4, D_2

Literature references: entry 1 (*Angew. Chem., Int. Ed.* **2016**, *55*, 7958–7962); entry 2 (*Angew., Chem. Int. Ed*. **2008**, *47*, 877–881); entry 3 (*Angew. Chem., Int. Ed*. **2022**, doi/10.1002/anie.202214577.); entry 4 (*Nat. Chem*. **2020**, *12*, 574–578); entry 5 (*J. Am. Chem. Soc*. **2012**, *134*, 19334–19337); entry 6 (*J. Am. Chem. Soc*. **2022**, *144*, 8467−8473); entry 7 is the ligand for the cages in this work.

8 X-ray Crystallography

Crystallographic data were deposited with the CCDC (2180301).

Table S3. Crystal data and structure refinement for hl426_sq.

Identification code	hl426_sq	
Empirical formula	$C_{280}H_{188}F_{24}N_{48}O_{56}S_{16}Zn_6$	
Formula weight	6481.95	
Temperature	100(2) K	
Wavelength	0.6889 Å	
Crystal system	Monoclinic	
Space group	C 2/c	
Unit cell dimensions	a = 48.950(4) Å	α = 90°.
	b = 43.9701(16) Å	β = 128.379(3)°.
	c = 30.5721(16) Å	γ = 90°.
Volume	51583(5) $Å^3$	
Z	4	
Density (calculated)	0.835 Mg/m^3	
Absorption coefficient	0.363 mm^{-1}	
F(000)	13216	
Crystal size	0.030 x 0.030 x 0.030 mm^3	
Theta range for data collection	1.029 to 17.429°.	
Index ranges	-42<=h<=42, -38<=k<=38, -26<=l<=26	
Reflections collected	59056	
Independent reflections	17700 [R(int) = 0.1059]	
Completeness to theta = 17.429°	99.5%	
Absorption correction	Empirical	
Max. and min. transmission	1.0 and 0.8834182467612366	
Refinement method	Full-matrix least-squares on F^2	
Data / restraints / parameters	17700 / 2962 / 1661	
Goodness-of-fit on F^2	1.005	
Final R indices [I>2sigma(I)]	R1 = 0.0664, wR2 = 0.1567	
R indices (all data)	R1 = 0.1242, wR2 = 0.1660	
Extinction coefficient	n/a	
Largest diff. peak and hole	0.220 and -0.341 e.$Å^{-3}$	

Specific refinement details:

The crystals of $[(C_{14}H_8N_2O_{10}S_2)_4 \subset Zn^{II}_6L_4] \cdot 4NTf_2$ [+ solvent] were grown by diffusion of diethyl ether into an acetonitrile solution of the $Zn^{II}_9L_6$ assembly containing 6 equiv. of the disodium salt of 4,4'-dinitrostilbene-2,2'-disulfonic acid. Data were collected at Beamline I19 of Diamond Light Source employing silicon double crystal monochromated synchrotron radiation (0.6889 Å) with ω and ψ scans at 100(2) K.[4] Data integration and reduction were undertaken with Xia2.[5] Subsequent computations were carried out using the WinGX-32 graphical user interface.[6] A multi-scan empirical absorption correction using spherical harmonics was applied to the data using DIALS.[5b] The structure was solved by intrinsic phasing using SHELXT[7] then refined and extended with SHELXL.[8] Carbon-bound hydrogen atoms were included in idealized positions and refined using a riding model. Disorder was modelled using standard crystallographic methods including constraints and restraints where necessary.

The crystals employed immediately lost solvent after removal from the mother liquor and rapid handling prior to flash cooling in liquid nitrogen was required to collect data. Despite these measures and the use of synchrotron radiation few reflections at greater than 1.15 Å resolution were observed and the data were trimmed accordingly. Furthermore, there was a significant drop-off in diffraction intensity after around 1.5 Å resolution resulting in a low ratio of observed/unique reflections. Nevertheless, the quality of the data is far more than sufficient to establish the connectivity of the structure. The asymmetric unit was found to contain one half of a $Zn^{II}_6L_4$ assembly and two 4,4'-dinitrostilbene-2,2'-disulfonate guests as well as associated counterions and solvent molecules.

Due to the limited resolution, the bond lengths, and angles of the two chemically identical organic ligands were restrained to be similar to each other (SAME). Likewise, the two 4,4'-dinitrostilbene-2,2'-disulfonate guests were restrained to have similar bond lengths and angles to each other. Additional DFIX restraints were applied to some parts of the structure displaying higher degrees of thermal motion. Thermal parameter restraints (SIMU, RIGU) were applied to all atoms except for zinc to facilitate a stable

anisotropic refinement.

Four additional anions per $Zn^{II}_6L_4$ assembly (i.e., two per asymmetric unit) are required for charge balance. These anions (included as triflimide in the formula below) were significantly disordered and despite numerous attempts at modelling, including with rigid bodies no satisfactory model for the electron-density associated with them could be found. Therefore, the SQUEEZE[9] function of PLATON[10] was employed to remove the contribution of the electron density associated with the remaining anions and further highly disordered solvent, which gave a potential solvent accessible void of 31083 Å^3 per unit cell (a total of approximately 9478 electrons). Diffuse solvent molecules could not be assigned to acetontrile or diethyl ether and were therefore not included in the formula. Consequently, the molecular weight and density given above are underestimated.

CheckCIF gives one A and two B level alerts. These alerts mostly result from the limited resolution of the data with one alert also resulting from a short contact between two ligand protons which appears to be a genuine feature of the structure, arising from the preferred conformation of the ligand.

9 Volume Calculations

To determine the available void space within the PM7-optimized molecular models[11] of **1**-S_4, **1**-D_2 and the crystal structure of **3**-S_4, and the volumes of anions $B(p\text{-}C_6H_4Cl)_4^-$ (CCDC, 748991), $B(p\text{-}C_6H_4F)_4^-$ (708376), IO_4^- (2168128), ReO_4^- (821877), $PO_4(WO_3)_{12}^{3-}$ (789283) based on the crystal structures, MoloVol calculations[12] were performed. In each case a virtual probe with the smallest radius such that it would not exit the host cavity during calculations was selected. The standard parameters given below were used and the results are given in Tables S4 and S5.

Optimization depth: 4

Table S4. Calculated cavity volumes of the complexes.

Complex	Probe radius (Å)	Plot grid spacing	Cavity volume (Å^3)
**1**-S_4	2.8	0.2	411.0
**1**-D_2	2.8	0.2	452.7
**3**-S_4	2.8	0.2	558.9

Table S5. Calculated Van der Waals volumes of guests.

Guest	Probe radius (Å)	Plot grid spacing	Volume (Å^3)
$B(p\text{-}C_6H_4Cl)_4^-$	0.5	0.2	386.8
$B(p\text{-}C_6H_4F)_4^-$	0.5	0.2	339.6
IO_4^-	0.2	0.2	64.2
ReO_4^-	0.2	0.2	77.3
$[PO_4(WO_3)_{12}]^{3-}$	0.5	0.2	692.4

10 Optimized Structures of $Zn^{II}_6L_4$ and $Zn^{II}_9L_6$ Cages

Geometry optimized structures were modelled at the PM7 level of theory[11] using the program MOPAC2016 (version 21.041W)[13] with the following parameters: RHF (restricted Hartree- Fock Hamiltonian), LBFGS (low memory Broyden-Fletcher-Goldfarb-Shanno procedure), a maximum of 2000 SCF (self-consistent field) iterations and a SCF criterion at 10^{-4} kcal/mol. The cartesian coordinates of these models are given in Tables S8-S13 below.

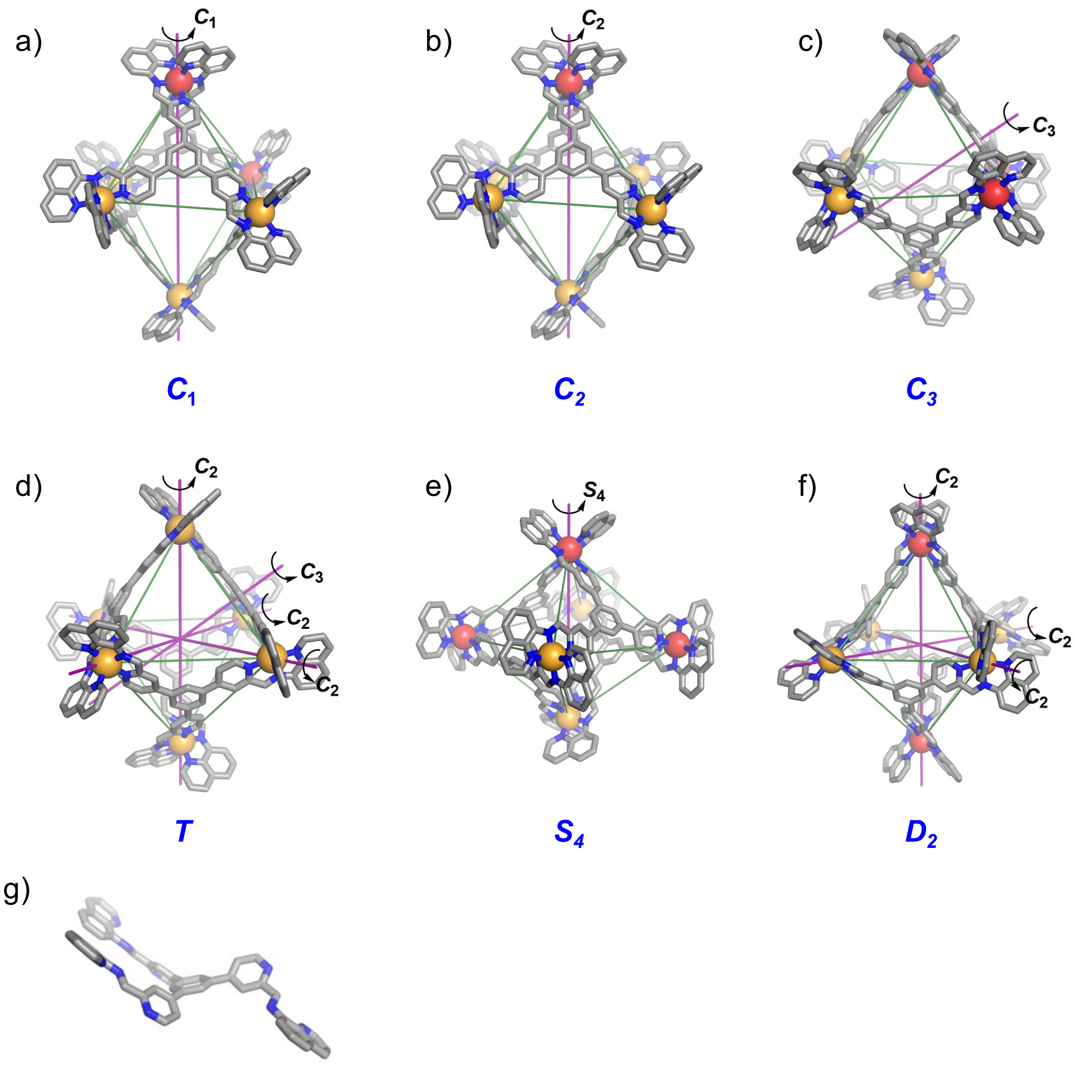

Figure S64. PM7-optimized molecular model of all diastereomer of **1** and of the ligand formed when **A** and **B** condense. Zn^{II} centers with Δ or Λ configuration are colored yellow and red, respectively. a) **1** with C_1 configuration, b) **1** with C_2 configuration, c) **1** with C_3 configuration, d) **1** with T configuration, e) **1** with S_4 configuration, f) **1** with D_2 configuration, g) Ligand.

Table S6. Antipodal Zn···Zn distances and dihedral angles between phenylene and pyridyl rings on the same ligand for crystal structure and models with different configurations optimized using the PM7[11] force field of Scigress[14].

Entry[a]	Configuration	Antipodal Zn-Zn (Å)	Antipodal Zn-Zn (Å)	Antipodal Zn-Zn (Å)	Dihedral angles between phenylene and pyridyl rings (°)
1	$Zn^{II}_6L_4$, *T*	18.592	18.645	18.656	14.00, 15.14, 17.14
2	$Zn^{II}_6L_4$, C_1	17.665	18.618	19.399	14.75, 16.01, 28.91,<br>19.67, 22.57, 23.96<br>14.09, 15.85, 24.63,<br>9.70, 22.82, 24.75
3	$Zn^{II}_6L_4$, C_2	17.666	18.673	19.404	16.05, 21.52, 25.23<br>9.17, 20.74, 23.55
4	$Zn^{II}_6L_4$, C_3	18.428	18.514	18.698	10.82, 16.74, 18.93<br>21.86, 22.86, 22.85
5	$Zn^{II}_6L_4$, D_2	18.184	19.497	20.346	**37.35, 40.40, 40.87**
6	$Zn^{II}_6L_4$, S_4	16.067	19.727	20.154	**19.66, 33.03, 37.00**
7[b]	**3**-S_4 (crystal)	16.069	20.551	20.641	**16.43, 33.23, 35.38**
8[c]	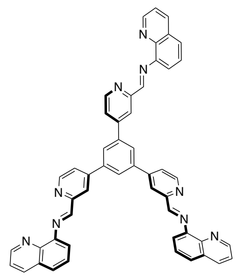	-	-	-	**41.06, 52.76, 54.43**

[a]8-aminoquinoline **B** as subcomponent was incorporated. [b]2-methyl-8-aminoquinoline **C** was incorporated as subcomponent; the structure of **3**-S_4 is the structure of the crystal $(\mathbf{G})_4\subset\mathbf{3}$ with guests removed. [c]Free ligand for the construction of cage by the condensation of **A** and **B**.

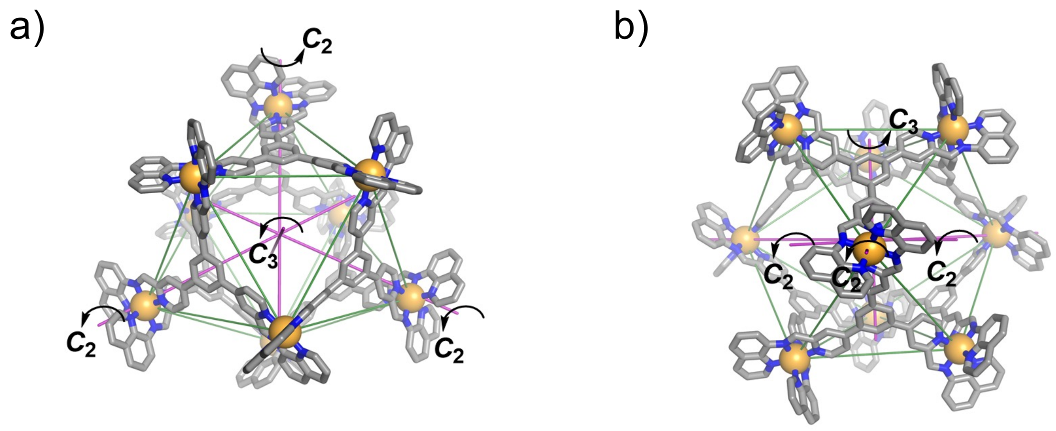

Figure S65. PM7-optimized molecular model of $Zn^{II}_9L_6$ with D_3 symmetry (**A** and **B** as subcomponents). Zinc atoms, all having the Δ configuration, are colored yellow. a) View down the C_3 axis, b) View down the C_2 axis.

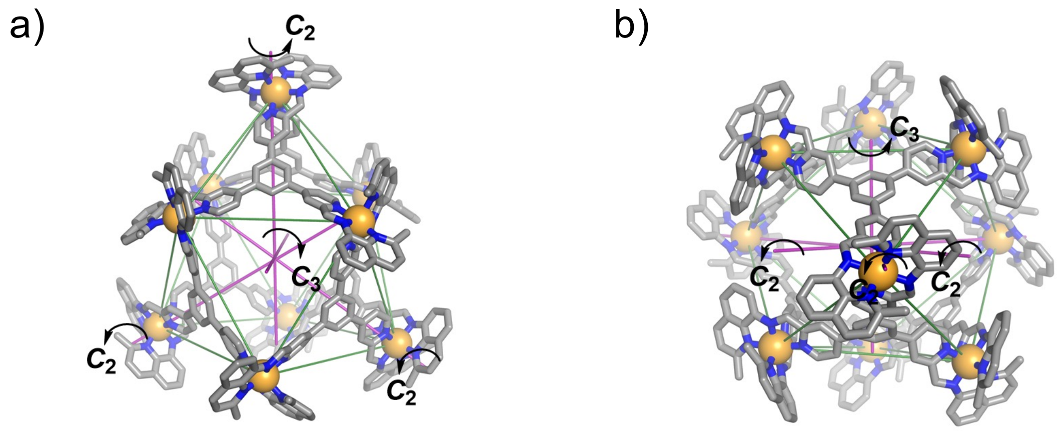

Figure S66. PM7-optimized molecular model of $Zn^{II}_9L_6$ **2** with D_3 symmetry (**A** and **C** as subcomponents). Zinc atoms, all having the Δ configuration, are colored yellow. a) View down the C_3 axis, b) View down the C_2 axis.

Table S7. Dihedral angles between phenylene and pyridyl rings on the same ligand between D_3-symmetric $Zn^{II}_9L_6$ cages and S_4-symmetric $Zn^{II}_6L_4$ cages, incorporating 8-aminoquinoline **B** or 2-methyl-8-aminoquinoline **C**, optimized using PM7 force field of Scigress [14].

Entry	Cage	Dihedral angles phenylene and pyridyl rings (°)	Dihedral angles between quinoline and pyridyl rings
1[a]	$Zn^{II}_6L_4$, S_4	19.66, 33.03, 37.00	14.1
2[b]	**3**-S_4 (methyl, crystal)	16.43, 33.23, 35.38	11.3
3[a]	$Zn^{II}_9L_6$	4.97, 11.10, 28.00; 2.19, 19.50, 28.37; 4.27, 22.93, 35.14; 5.55, 26.00, 40.41; 17.28, 29.05, 41.22; 12.95, 31.58, 42.26	0.6
4[b]	$Zn^{II}_9L_6$ (methyl)	14.10, 18.34, 32.88; 2.13, 19.10, 38.13; 19.36, 30.14, 36.88; 20.20, 32.66, 40.20; 19.94, 28.53, 43.22; 14.96, 34.22, 46.46	25.5

[a]8-aminoquinoline **B** was used as subcomponent. [b]2-methyl-8-aminoquinoline **C** was incorporated as subcomponent. [c]The quinoline and pyridyl ring is on the same arm of the ligand.

Note: The introduction of methyl group, which led to the formation of a $Zn^{II}_9L_6$ framework, appears to lead to larger dihedral angles between quinolines and pyridyl rings (Table S7, entries 3-4), rendering the cage vertices more twisted. In comparing the dihedral angles between phenylene and pyridyl rings of the models (Table S7, entries 3-4), the introduction of methyl group during the construction of $Zn^{II}_9L_6$ also appears to increase the dihedral angles, leading to a reduction the steric hindrance between phenylene and pyridyl rings. Therefore, the presence of methyl groups introduces a twist into the vertices of cages, as the dihedral angles between quinolines and pyridyl rings become larger in $Zn^{II}_9L_6$ structure, and further affects the dihedral angles between phenylene and pyridyl rings, resulting in the reduction of steric hindrance. The analysis of previously-published structures (Table S7, entries 1-2) also suggests that the introduction of methyl groups would be unfavorable to the formation of M_6L_4 architectures.

Table S8. Cartesian coordinates (in Å) for the PM7 model of **1** with C_1 symmetry

C	6.0186	-6.4085	-5.3179	C	1.3544	-4.362	10.8422	H	7.4084	-8.4354	-4.1261
C	6.8566	-5.8495	-4.4375	C	1.5476	-3.9919	9.5119	H	4.5664	-11.5726	-4.8498
C	7.0692	-4.3937	-4.4782	N	4.7906	-5.4663	8.8768	H	3.7872	-7.6131	-6.3175
N	6.4736	-3.6475	-5.326	C	5.7155	-6.1492	9.4405	C	-5.1332	-6.9376	-1.8316
C	5.6704	-4.146	-6.2062	C	5.6209	-6.596	10.8424	C	-4.6901	-7.4542	-3.0548
C	5.4093	-5.4627	-6.2761	C	4.5358	-6.2771	11.544	C	-4.2801	-8.7845	-3.1984
C	5.0043	-3.1861	-7.1427	H	4.9964	-3.3571	2.0713	C	-4.3769	-9.6242	-2.0829
N	5.2676	-1.946	-6.9773	H	5.8773	-4.4153	4.178	C	-4.8764	-9.1683	-0.8577
C	4.9336	-0.8266	-7.504	H	1.5168	-2.2748	4.4483	C	-5.2416	-7.8214	-0.7516
C	5.5031	0.3525	-6.9395	H	1.3662	-2.9614	6.8184	H	-3.991	-10.6502	-2.1468
C	5.1593	1.5988	-7.4861	H	2.1628	-5.3943	12.5617	H	-5.5776	-7.4429	0.2215
C	4.2748	1.6756	-8.5649	H	0.4312	-4.065	11.3679	H	-4.6159	-6.7847	-3.9193
C	3.7221	0.5177	-9.1103	H	0.7723	-3.4042	8.997	C	4.8417	-12.8633	2.4137
C	4.0481	-0.7307	-8.5813	H	6.6135	-6.4124	8.86	C	4.5309	-13.7156	1.4302
N	6.3191	0.2421	-5.9381	H	6.4375	-7.1826	11.2931	C	5.6017	-14.2173	0.5537
C	6.8554	1.2714	-5.3972	H	4.4207	-6.5895	12.5948	N	6.8187	-13.8601	0.7014
C	6.5833	2.641	-5.8704	C	3.3543	-8.9898	5.2954	C	7.1683	-13.0583	1.6516
C	5.7516	2.8104	-6.8955	C	2.3897	-8.2125	5.8049	C	6.2816	-12.5479	2.5235
H	7.3645	-6.4269	-3.6518	C	2.7449	-6.9017	6.3728	C	8.6153	-12.6809	1.7275
H	7.7377	-3.9337	-3.7339	N	3.9482	-6.4771	6.394	N	9.3825	-13.1606	0.824
H	4.7091	-5.8249	-7.0422	C	4.9139	-7.1946	5.9249	C	10.6195	-13.096	0.4972
H	4.3086	-3.5354	-7.9206	C	4.7123	-8.4116	5.3906	C	11.024	-13.8213	-0.662
H	4.0094	2.6577	-8.9899	C	6.2897	-6.6065	5.9821	C	12.3698	-13.7697	-1.0571
H	3.0243	0.59	-9.9617	N	6.3928	-5.4333	6.4799	C	13.2916	-13.0193	-0.3228
H	3.6033	-1.6363	-9.0212	C	7.3198	-4.5794	6.7108	C	12.8872	-12.3124	0.8088
H	7.5446	1.131	-4.5499	C	6.9301	-3.3418	7.3021	C	11.5547	-12.3483	1.2181
H	7.0639	3.5024	-5.3796	C	7.9161	-2.3791	7.569	N	10.1395	-14.5034	-1.3203
H	5.5177	3.8153	-7.2837	C	9.2543	-2.636	7.2603	C	10.4534	-15.1686	-2.3684
C	3.4951	-1.4153	-1.8405	C	9.6266	-3.8491	6.6828	C	11.8299	-15.2032	-2.8953
C	2.9538	-1.5835	-3.0514	C	8.6631	-4.8186	6.4068	C	12.7767	-14.5192	-2.2572
C	3.842	-1.6346	-4.2248	N	5.6789	-3.1395	7.5758	H	3.4975	-14.0231	1.2152
N	5.1108	-1.5276	-4.1184	C	5.2804	-2.0447	8.1072	H	5.3366	-14.8909	-0.2761
C	5.6709	-1.342	-2.9693	C	6.2079	-0.9481	8.4401	H	6.6403	-11.86	3.3021
C	4.9638	-1.2622	-1.8308	C	7.5036	-1.1042	8.1789	H	8.9899	-12.0042	2.5104
C	7.1645	-1.2516	-2.9406	H	1.3284	-8.4949	5.803	H	14.347	-12.9822	-0.6391
N	7.768	-1.3922	-4.0582	H	1.9438	-6.2623	6.7755	H	13.623	-11.7215	1.38
C	8.9748	-1.4063	-4.4876	H	5.5813	-8.9649	5.0093	H	11.2489	-11.7834	2.1119
C	9.1639	-1.6235	-5.8839	H	7.1639	-7.1575	5.6033	H	9.6697	-15.7344	-2.8961
C	10.4718	-1.6495	-6.3929	H	10.0239	-1.8762	7.474	H	12.0618	-15.7864	-3.8009
C	11.5639	-1.4659	-5.541	H	10.6862	-4.0418	6.444	H	13.8195	-14.5145	-2.6145
C	11.3696	-1.2562	-4.1765	H	8.9716	-5.7716	5.9505	C	6.8343	-11.0346	-3.5036
C	10.0791	-1.227	-3.6494	H	4.2086	-1.9164	8.3248	C	7.6121	-10.5922	-2.51
N	8.126	-1.7927	-6.6429	H	5.8275	-0.0204	8.8972	C	8.162	-11.5663	-1.5527
C	8.2456	-1.9911	-7.9021	H	8.2342	-0.312	8.4108	N	7.9149	-12.8164	-1.644
C	9.5593	-2.0414	-8.5696	Zn	4.6647	-4.7479	7.0409	C	7.2002	-13.2881	-2.6104
C	10.6571	-1.8744	-7.8359	C	-5.3677	-5.4667	-1.6523	C	6.6613	-12.5003	-3.5537
H	1.8742	-1.7344	-3.1985	C	-4.9885	-4.5625	-2.564	C	6.9468	-14.763	-2.6211
H	3.3999	-1.8062	-5.2186	C	-5.0915	-3.131	-2.2401	N	7.4321	-15.4332	-1.6476
H	5.4982	-1.1132	-0.8813	N	-5.5308	-2.7246	-1.113	C	7.4476	-16.6511	-1.251

H	7.7109	-1.0929	-1.9984	C	-5.9423	-3.5579	-0.2172	C	8.1309	-16.9267	-0.0305
H	12.5886	-1.488	-5.9473	C	-5.9121	-4.8875	-0.4058	C	8.1655	-18.2483	0.441
H	12.239	-1.1143	-3.5125	C	-6.4238	-2.9846	1.0792	C	7.5399	-19.2701	-0.2782
H	9.9405	-1.0613	-2.57	N	-6.3541	-1.7145	1.2051	C	6.8725	-18.9908	-1.47
H	7.3372	-2.1285	-8.5091	C	-6.6221	-0.8341	2.0962	C	6.8239	-17.6847	-1.9556
H	9.6222	-2.2138	-9.656	C	-6.335	0.5246	1.7729	N	8.697	-15.9516	0.6104
H	11.6606	-1.9037	-8.2911	C	-6.6087	1.5187	2.7251	C	9.3207	-16.1496	1.7108
Zn	6.5113	-1.6766	-5.5107	C	-7.157	1.1761	3.9638	C	9.436	-17.4871	2.3202
C	-4.8984	-10.0712	0.3349	C	-7.4357	-0.155	4.2709	C	8.8717	-18.5228	1.7033
C	-4.8211	-9.6107	1.5881	C	-7.166	-1.1592	3.3421	H	7.8056	-9.5228	-2.3414
C	-4.7678	-10.5751	2.6993	N	-5.8267	0.8037	0.6131	H	8.7641	-11.1986	-0.7072
N	-4.7875	-11.8371	2.5019	C	-5.5484	2.0077	0.2782	H	6.044	-12.9612	-4.338
C	-4.8756	-12.3241	1.3086	C	-5.7841	3.1494	1.1806	H	6.3495	-15.236	-3.4154
C	-4.9445	-11.5428	0.2173	C	-6.3053	2.9181	2.3831	H	7.5707	-20.3061	0.0976
C	-4.8713	-13.8153	1.177	H	-4.5475	-4.836	-3.5322	H	6.3805	-19.8047	-2.0291
N	-4.7579	-14.4866	2.259	H	-4.737	-2.3932	-2.9768	H	6.2919	-17.478	-2.8969
C	-4.695	-15.7175	2.6093	H	-6.2545	-5.537	0.4113	H	9.7881	-15.2927	2.2205
C	-4.5475	-15.9901	4.0012	H	-6.7962	-3.631	1.8883	H	9.9809	-17.6181	3.2689
C	-4.4706	-17.326	4.425	H	-7.3719	1.9619	4.7064	H	8.9313	-19.5409	2.1217
C	-4.5391	-18.3656	3.4942	H	-7.8697	-0.4141	5.2515	Zn	8.3985	-14.2885	-0.4127
C	-4.6828	-18.0902	2.1351	H	-7.3898	-2.2061	3.5981	C	-3.6593	-9.2634	-4.4756
C	-4.7589	-16.7702	1.692	H	-5.1199	2.1991	-0.718	C	-3.1944	-8.4253	-5.4101
N	-4.4867	-14.9993	4.8352	H	-5.5326	4.1722	0.8569	C	-2.5255	-8.9769	-6.5996
C	-4.3538	-15.1931	6.0938	H	-6.5003	3.74	3.0912	N	-2.3669	-10.2328	-6.7636
C	-4.2607	-16.5443	6.6764	C	-0.9537	-1.1931	0.5081	C	-2.8052	-11.0785	-5.8912
C	-4.317	-17.5968	5.8639	C	-1.7844	-1.6571	1.4497	C	-3.4451	-10.6943	-4.7742
H	-4.7573	-8.5376	1.82	C	-3.2385	-1.5482	1.2397	C	-2.5512	-12.5292	-6.1576
H	-4.6803	-10.1945	3.7289	N	-3.734	-1.0269	0.1841	N	-1.9064	-12.8109	-7.2243
H	-5.0152	-12.0145	-0.7736	C	-2.9706	-0.5415	-0.737	C	-1.4744	-13.8594	-7.8197
H	-4.9442	-14.3034	0.1934	C	-1.6308	-0.568	-0.6481	C	-0.7684	-13.6665	-9.043
H	-4.4789	-19.4127	3.8339	C	-3.6376	0.0298	-1.9493	C	-0.2756	-14.7907	-9.7237
H	-4.7358	-18.9191	1.4089	N	-4.9133	-0.038	-1.9872	C	-0.4781	-16.0731	-9.2069
H	-4.8705	-16.5677	0.616	C	-5.8556	0.287	-2.7917	C	-1.1718	-16.2525	-8.011
H	-4.3079	-14.3223	6.7662	C	-7.1876	-0.0253	-2.3905	C	-1.6676	-15.1494	-7.3171
H	-4.1468	-16.6716	7.765	C	-8.2478	0.3114	-3.2467	N	-0.6004	-12.4614	-9.4914
H	-4.2507	-18.6259	6.2536	C	-7.9954	0.9412	-4.4681	C	0.0265	-12.2378	-10.5853
C	0.0577	-12.5389	3.9499	C	-6.6893	1.24	-4.8535	C	0.5967	-13.3249	-11.402
C	-0.3886	-13.3464	2.9815	C	-5.6209	0.9127	-4.0195	C	0.4533	-14.581	-10.9856
C	-1.8283	-13.649	2.9143	N	-7.3823	-0.6131	-1.2511	H	-3.2597	-7.3322	-5.3164
N	-2.6625	-13.1588	3.7487	C	-8.56	-0.9159	-0.8497	H	-2.1296	-8.2828	-7.3572
C	-2.27	-12.3934	4.7118	C	-9.7642	-0.6195	-1.6473	H	-3.7894	-11.466	-4.0714
C	-0.981	-12.0618	4.8854	C	-9.6196	-0.0164	-2.8249	H	-2.8924	-13.3083	-5.4591
C	-3.3209	-11.848	5.6271	H	-1.4306	-2.1614	2.3595	H	-0.0885	-16.9519	-9.7468
N	-4.5299	-12.1676	5.3652	H	-3.9176	-1.9644	2.0001	H	-1.328	-17.2694	-7.6128
C	-5.6957	-11.934	5.8419	H	-1.0458	-0.1478	-1.4777	H	-2.2121	-15.3058	-6.3733
C	-6.8026	-12.5293	5.169	H	-3.0522	0.4643	-2.774	H	0.1454	-11.1984	-10.929
C	-8.0968	-12.2951	5.6593	H	-8.8331	1.2036	-5.1352	H	1.1298	-13.0929	-12.3379
C	-8.2898	-11.4916	6.7861	H	-6.5011	1.7346	-5.8214	H	0.863	-15.4261	-11.5631
C	-7.2013	-10.9126	7.4369	H	-4.5948	1.1522	-4.3376	C	2.7628	-10.1376	-6.2725
C	-5.907	-11.1316	6.9669	H	-8.6797	-1.4135	0.1253	C	2.1273	-11.2119	-5.7906
N	-6.5843	-13.2673	4.1251	H	-10.7614	-0.8946	-1.268	C	0.8299	-11.5948	-6.3705

C	-7.5463	-13.8226	3.4881	H	-10.4907	0.2254	-3.4557	N	0.2885	-10.9258	-7.3136
C	-8.9549	-13.6618	3.893	Zn	-5.6245	-0.8847	-0.3913	C	0.8644	-9.8787	-7.8051
C	-9.2336	-12.9136	4.9577	C	0.5353	-1.3748	0.5707	C	2.0563	-9.44	-7.3657
H	0.2703	-13.7556	2.2022	C	1.3181	-1.207	-0.5785	C	0.1393	-9.1479	-8.8915
H	-2.1994	-14.2882	2.098	C	2.6897	-1.4767	-0.5833	N	-0.9975	-9.6175	-9.2389
H	-0.7159	-11.3964	5.7192	C	3.2823	-1.9202	0.6021	C	-1.9257	-9.3304	-10.074
H	-3.0609	-11.1902	6.4702	C	2.5442	-2.1083	1.7757	C	-3.0632	-10.1894	-10.1007
H	-9.3091	-11.3117	7.1656	C	1.173	-1.8216	1.7372	C	-4.1015	-9.9092	-11.0027
H	-7.365	-10.2792	8.3252	H	4.348	-2.1804	0.5857	C	-4.0161	-8.8053	-11.855
H	-5.057	-10.6681	7.4907	H	0.5719	-2.0022	2.6363	C	-2.9007	-7.9698	-11.8211
H	-7.3153	-14.4425	2.6077	H	0.8424	-0.9002	-1.5185	C	-1.8572	-8.2305	-10.9338
H	-9.7557	-14.1558	3.3195	C	3.0739	-10.3177	4.6489	N	-3.1072	-11.2037	-9.2942
H	-10.2717	-12.7655	5.2981	C	4.0589	-10.9999	3.9221	C	-4.1065	-12.0044	-9.2828
Zn	-4.6353	-13.32	3.8062	C	3.7972	-12.2119	3.2682	C	-5.2658	-11.8293	-10.1768
C	3.1805	-2.6961	3.0014	C	2.5034	-12.7406	3.3496	C	-5.2711	-10.8033	-11.0246
C	4.3819	-3.2871	2.9794	C	1.4756	-12.0653	4.0141	H	2.5242	-11.8129	-4.9597
C	4.8972	-3.9138	4.2078	C	1.7864	-10.8677	4.6653	H	0.3063	-12.4756	-5.9669
N	4.2303	-3.9172	5.2964	H	2.2766	-13.6903	2.8473	H	2.5016	-8.5474	-7.8288
C	3.0726	-3.3493	5.3681	H	0.9747	-10.3202	5.1575	H	0.5648	-8.2462	-9.3569
C	2.5065	-2.7334	4.3165	H	5.0508	-10.5452	3.8099	H	-4.8352	-8.5906	-12.5612
C	2.3566	-3.4213	6.6806	C	4.0721	-9.6657	-5.7239	H	-2.8437	-7.1006	-12.498
N	2.946	-4.0552	7.621	C	4.4678	-8.3276	-5.835	H	-0.9821	-7.5629	-10.9184
C	2.7165	-4.3656	8.8425	C	5.6656	-7.8631	-5.2742	H	-4.1015	-12.8546	-8.5828
C	3.7147	-5.1282	9.5169	C	6.4688	-8.7774	-4.5802	H	-6.1073	-12.5396	-10.1387
C	3.4994	-5.4899	10.8559	C	6.0819	-10.1092	-4.4036	H	-6.1152	-10.6332	-11.713
C	2.3256	-5.1068	11.5099	C	4.8922	-10.5356	-4.9997	Zn	-1.4486	-11.2087	-8.2211

Table S9. Cartesian coordinates (in Å) for the PM7 model of **1** with C_2 symmetry

C	5.8393	-5.3835	-5.5077	C	0.3377	-2.8378	10.4502	H	-8.148	-3.397	3.0749
C	6.5566	-4.6539	-4.6455	C	0.526	-2.5974	9.0897	H	-6.8001	0.6231	-1.9462
C	6.5501	-3.1876	-4.7734	N	3.9777	-3.6142	8.7064	H	-7.6631	2.6912	-0.7122
N	5.8762	-2.5919	-5.6799	C	4.972	-4.0758	9.3681	H	-8.5568	2.4338	1.5789
C	5.1949	-3.2575	-6.5517	C	4.8901	-4.3677	10.8109	C	-2.0348	-1.5025	-0.189
C	5.1408	-4.5994	-6.5476	C	3.7422	-4.1423	11.4456	C	-2.778	-1.9753	0.8198
C	4.4179	-2.4693	-7.5597	H	4.1173	-2.2928	1.6908	C	-4.2087	-2.2405	0.5969
N	4.4676	-1.1965	-7.4538	H	5.0752	-2.9618	3.9327	N	-4.7676	-2.0319	-0.5314
C	3.9732	-0.1755	-8.0488	H	0.4427	-1.4767	3.8669	C	-4.0992	-1.5504	-1.5252
C	4.3167	1.1075	-7.5302	H	0.3018	-1.9153	6.2923	C	-2.79	-1.2607	-1.4377
C	3.7916	2.2512	-8.1517	H	1.2238	-3.5273	12.2981	C	-4.8389	-1.3482	-2.811
C	2.9476	2.1272	-9.2583	H	-0.6401	-2.6286	10.9162	N	-6.0694	-1.6944	-2.8257
C	2.616	0.8685	-9.758	H	-0.3076	-2.1988	8.4916	C	-7.0386	-1.7157	-3.6632
C	3.1253	-0.2809	-9.155	H	5.9222	-4.2611	8.8432	C	-8.2837	-2.2365	-3.2026
N	5.1006	1.1827	-6.5	H	5.7676	-4.764	11.3465	C	-9.3687	-2.2808	-4.092
C	5.4369	2.3115	-5.9979	H	3.6341	-4.3449	12.5237	C	-9.2247	-1.8203	-5.4033
C	4.9596	3.5921	-6.5508	C	3.2512	-7.7075	5.549	C	-8.0032	-1.3141	-5.8454
C	4.1517	3.5713	-7.6083	C	2.1531	-7.041	5.9256	C	-6.9116	-1.2627	-4.9794
H	7.1095	-5.0989	-3.806	C	2.2766	-5.6325	6.3393	N	-8.3788	-2.6563	-1.9793
H	7.1082	-2.5881	-4.0374	N	3.3991	-5.0229	6.3444	C	-9.4749	-3.1333	-1.5207
H	4.5266	-5.103	-7.3071	C	4.4845	-5.6291	5.9944	C	-10.6932	-3.238	-2.3444
H	3.8164	-2.9668	-8.3356	C	4.498	-6.9165	5.6116	C	-10.6495	-2.8214	-3.6077
H	2.5384	3.0295	-9.7421	C	5.7506	-4.831	6.0107	H	-2.3646	-2.2136	1.8089
H	1.9482	0.7823	-10.6317	N	5.6518	-3.606	6.3602	H	-4.8062	-2.6587	1.422
H	2.8541	-1.268	-9.5595	C	6.4271	-2.5962	6.5028	H	-2.2845	-0.8699	-2.331
H	6.1067	2.3284	-5.1241	C	5.8297	-1.3748	6.9315	H	-4.338	-0.9376	-3.7008
H	5.2717	4.5439	-6.0916	C	6.6454	-0.2448	7.0989	H	-10.0813	-1.8572	-6.0963
H	3.7705	4.5037	-8.0561	C	8.0178	-0.3225	6.8479	H	-7.9005	-0.9537	-6.883
C	2.4473	-0.771	-2.3978	C	8.5934	-1.521	6.4283	H	-5.953	-0.8613	-5.3422
C	1.9865	-1.0704	-3.6173	C	7.8016	-2.6556	6.2556	H	-9.5111	-3.4765	-0.4749
C	2.9097	-1.0073	-4.7629	N	4.5529	-1.3412	7.1546	H	-11.6185	-3.6538	-1.9144
N	4.1368	-0.6795	-4.6257	C	3.9713	-0.2682	7.5423	H	-11.5354	-2.8796	-4.2611
C	4.6154	-0.3554	-3.4707	C	4.7061	0.9915	7.7583	Zn	-6.6265	-2.3998	-1.1047
C	3.8653	-0.358	-2.3566	C	6.0192	1.0117	7.5424	C	-0.5475	-1.3116	-0.0844
C	6.0704	-0.0096	-3.4049	H	1.1514	-7.4931	5.9165	C	0.2258	-1.0097	-1.2132
N	6.7301	-0.0949	-4.4963	H	1.3687	-5.0838	6.6351	C	1.6241	-0.9477	-1.1603
C	7.9373	0.0747	-4.8902	H	5.4587	-7.3703	5.3312	C	2.2507	-1.171	0.0707
C	8.2118	-0.1735	-6.2671	H	6.7139	-5.2792	5.7235	C	1.5209	-1.4664	1.226
C	9.5235	-0.0035	-6.7367	H	8.6529	0.5687	6.9818	C	0.1274	-1.534	1.123
C	10.5375	0.4011	-5.8647	H	9.678	-1.5713	6.2331	H	3.3473	-1.1669	0.1189
C	10.2604	0.6399	-4.5194	H	8.2696	-3.595	5.924	H	-0.4429	-1.8279	2.0114
C	8.9645	0.4761	-4.0315	H	2.885	-0.285	7.7218	H	-0.2695	-0.8627	-2.1806
N	7.2457	-0.5513	-7.0451	H	4.1706	1.8946	8.0924	C	3.2224	-9.1261	5.0622
C	7.4432	-0.7871	-8.288	H	6.6103	1.9303	7.6909	C	4.2905	-9.6477	4.3221
C	8.7706	-0.6479	-8.9143	Zn	3.8181	-3.1393	6.7954	C	4.2717	-10.9505	3.8131
C	9.7972	-0.263	-8.1598	C	-3.0545	-10.8422	-3.821	C	3.1404	-11.7377	4.0479
H	0.9572	-1.4153	-3.7921	C	-2.1594	-11.8322	-3.7243	C	2.0226	-11.2369	4.7238
H	2.539	-1.2921	-5.7599	C	-1.4202	-12.2472	-4.9297	C	2.0938	-9.937	5.2399
H	4.3364	-0.0858	-1.4013	N	-1.6018	-11.6865	-6.0636	H	3.118	-12.7622	3.6538

H	6.5478	0.2787	-2.4562	C	-2.4627	-10.7337	-6.2014	H	1.2182	-9.5255	5.7563
H	11.5659	0.5319	-6.2399	C	-3.2062	-10.2818	-5.1792	H	5.1422	-8.9996	4.0773
H	11.0688	0.9574	-3.8392	C	-2.5943	-10.1176	-7.5585	C	4.2855	-8.8607	-5.702
H	8.7602	0.6663	-2.9667	N	-1.8316	-10.5797	-8.4729	C	4.5719	-7.5119	-5.9454
H	6.5937	-1.1044	-8.9125	C	-1.6033	-10.361	-9.714	C	5.6584	-6.8616	-5.349
H	8.9017	-0.8594	-9.9877	C	-0.6095	-11.1669	-10.3424	C	6.4981	-7.611	-4.518
H	10.8079	-0.1449	-8.584	C	-0.3305	-10.9574	-11.7021	C	6.231	-8.9512	-4.2194
Zn	5.5933	-0.6543	-5.9675	C	-1.017	-9.9744	-12.4198	C	5.1178	-9.5547	-4.8158
C	-4.4641	-11.1873	0.9983	C	-1.9865	-9.1903	-11.7963	H	7.367	-7.1269	-4.0527
C	-4.5296	-10.5553	2.1754	C	-2.2801	-9.382	-10.447	H	4.856	-10.581	-4.5269
C	-4.334	-11.3299	3.4121	N	0.0067	-12.0688	-9.6431	H	3.8948	-6.9313	-6.583
N	-4.0994	-12.5853	3.3933	C	0.9046	-12.8178	-10.1648	C	-4.962	-8.4134	-1.5791
C	-4.0559	-13.2394	2.2803	C	1.2971	-12.7066	-11.5817	C	-4.3328	-9.0017	-2.6837
C	-4.2377	-12.6443	1.0898	C	0.6962	-11.7953	-12.3431	C	-3.7989	-10.2946	-2.6419
C	-3.7485	-14.7023	2.3581	H	-1.9281	-12.3292	-2.7712	C	-3.8941	-11.0051	-1.441
N	-3.5196	-15.1786	3.5219	H	-0.6717	-13.0504	-4.8439	C	-4.5043	-10.4567	-0.3073
C	-3.2097	-16.3091	4.0385	H	-3.922	-9.4695	-5.3687	C	-5.0457	-9.1691	-0.403
C	-3.0253	-16.3479	5.4518	H	-3.305	-9.2993	-7.7499	H	-3.4314	-11.9979	-1.3689
C	-2.6784	-17.5667	6.0553	H	-0.7928	-9.8148	-13.4874	H	-5.5058	-8.7272	0.4889
C	-2.5188	-18.7175	5.279	H	-2.5225	-8.4168	-12.372	H	-4.1989	-8.4154	-3.5998
C	-2.7007	-18.6702	3.8975	H	-3.0481	-8.7559	-9.9674	C	5.3857	-11.4459	2.9486
C	-3.0434	-17.4695	3.2771	H	1.4017	-13.5722	-9.5353	C	5.2013	-12.3903	2.02
N	-3.1788	-15.2587	6.1385	H	2.0743	-13.3693	-11.995	C	6.3235	-12.7508	1.1389
C	-3.0212	-15.2432	7.4091	H	0.9565	-11.6756	-13.4076	N	7.4641	-12.1838	1.2328
C	-2.6568	-16.4511	8.1717	C	3.0659	-9.5132	-6.281	C	7.6903	-11.2784	2.126
C	-2.4869	-17.596	7.5147	C	2.0525	-8.8193	-6.8133	C	6.7477	-10.8766	2.9952
H	-4.6686	-9.4676	2.2565	C	0.8725	-9.5443	-7.3138	C	9.0539	-10.6606	2.1419
H	-4.3477	-10.8011	4.3779	N	0.7817	-10.8165	-7.2501	N	9.8779	-11.0667	1.2529
H	-4.1727	-13.2532	0.1767	C	1.7371	-11.5263	-6.7484	C	11.0844	-10.8235	0.8972
H	-3.6979	-15.3256	1.4525	C	2.8674	-10.9776	-6.2727	C	11.5925	-11.554	-0.2167
H	-2.2453	-19.6715	5.7592	C	1.5335	-13.0084	-6.6969	C	12.9099	-11.3152	-0.6381
H	-2.5708	-19.5844	3.2939	N	0.4223	-13.4504	-7.1474	C	13.7039	-10.3772	0.0267
H	-3.1818	-17.446	2.1854	C	-0.1589	-14.5833	-7.289	C	13.1979	-9.6657	1.1136
H	-3.1626	-14.296	7.9528	C	-1.4615	-14.5764	-7.8685	C	11.8916	-9.8865	1.5485
H	-2.5296	-16.3985	9.2649	C	-2.1323	-15.7973	-8.0402	N	10.8259	-12.416	-0.8091
H	-2.2134	-18.5244	8.0424	C	-1.5282	-16.9947	-7.6487	C	11.2376	-13.0965	-1.8124
C	0.7483	-12.0212	4.7969	C	-0.2541	-16.9923	-7.0828	C	12.5986	-12.9474	-2.3594
C	0.4924	-13.0463	3.9755	C	0.4299	-15.7906	-6.902	C	13.4258	-12.0749	-1.7887
C	-0.8434	-13.6637	3.9975	N	-1.9896	-13.4458	-8.2205	H	4.2298	-12.8802	1.8598
N	-1.7665	-13.247	4.7747	C	-3.1557	-13.3883	-8.746	H	6.163	-13.5104	0.3579
C	-1.5549	-12.2812	5.6052	C	-3.9721	-14.5927	-8.9832	H	6.9948	-10.0964	3.7295
C	-0.3708	-11.6526	5.689	C	-3.477	-15.7793	-8.6393	H	9.3217	-9.8841	2.8745
C	-2.7062	-11.8485	6.4585	H	2.0488	-7.722	-6.8742	H	14.7383	-10.1958	-0.3085
N	-3.8174	-12.4517	6.2704	H	0.036	-8.9685	-7.7399	H	13.8332	-8.9272	1.631
C	-5.0109	-12.4052	6.7333	H	3.6426	-11.6408	-5.8641	H	11.5056	-9.3181	2.4085
C	-5.9612	-13.3056	6.1686	H	2.3022	-13.6777	-6.2815	H	10.5534	-13.8195	-2.2831
C	-7.2809	-13.283	6.6463	H	-2.0604	-17.9502	-7.7877	H	12.9171	-13.5517	-3.2241
C	-7.6493	-12.3927	7.6583	H	0.2136	-17.9438	-6.7779	H	14.4527	-11.9298	-2.1622
C	-6.7136	-11.5148	8.2037	H	1.4351	-15.8041	-6.4537	C	7.0408	-9.6891	-3.2018
C	-5.3979	-11.5182	7.7419	H	-3.5645	-12.4064	-9.0314	C	7.7183	-9.061	-2.2348
N	-5.5823	-14.1165	5.2303	H	-4.9719	-14.503	-9.4378	C	8.3964	-9.8637	-1.2039

C	-6.3995	-14.9421	4.6918	H	-4.051	-16.707	-8.7979	N	8.3518	-11.14	-1.2038
C	-7.8146	-15.0262	5.0969	Zn	-0.702	-12.0081	-7.7997	C	7.7252	-11.7868	-2.1302
C	-8.2551	-14.2156	6.0562	C	-5.4256	-6.9845	-1.6163	C	7.0819	-11.1638	-3.1318
H	1.221	-13.4158	3.2401	C	-5.2129	-6.1777	-2.6638	C	7.712	-13.2803	-2.0311
H	-1.0631	-14.4799	3.2915	C	-5.5939	-4.7589	-2.5676	N	8.3028	-13.7931	-1.0201
H	-0.2636	-10.8251	6.4042	N	-6.1255	-4.2708	-1.5147	C	8.5178	-14.9612	-0.54
H	-2.6012	-11.0355	7.1928	C	-6.3777	-5.0158	-0.4913	C	9.2479	-15.0367	0.6823
H	-8.6871	-12.3813	8.03	C	-6.0823	-6.3259	-0.4663	C	9.4999	-16.2987	1.2429
H	-7.0152	-10.8158	9.0022	C	-6.9958	-4.3536	0.7004	C	9.0389	-17.4559	0.6099
H	-4.6698	-10.8191	8.181	N	-7.1995	-3.0948	0.6153	C	8.3244	-17.3726	-0.5843
H	-6.0341	-15.6145	3.8999	C	-7.6675	-2.1584	1.3536	C	8.0634	-16.1292	-1.1587
H	-8.4875	-15.7507	4.6108	C	-7.6729	-0.8417	0.8065	N	9.6564	-13.9408	1.2422
H	-9.3045	-14.2435	6.393	C	-8.1741	0.2124	1.5862	C	10.3155	-13.9576	2.3398
Zn	-3.6614	-13.807	4.8889	C	-8.6575	-0.0315	2.8743	C	10.6511	-15.2124	3.0373
C	2.2011	-1.8112	2.5147	C	-8.6464	-1.3219	3.402	C	10.254	-16.3662	2.5057
C	3.4753	-2.2142	2.5798	C	-8.1517	-2.3835	2.6454	H	7.7514	-7.9649	-2.1521
C	4.0319	-2.6129	3.8842	N	-7.2138	-0.6538	-0.3918	H	8.929	-9.3448	-0.3918
N	3.3334	-2.5931	4.9538	C	-7.1971	0.51	-0.9253	H	6.557	-11.7671	-3.8865
C	2.1028	-2.2014	4.9362	C	-7.6932	1.7059	-0.2201	H	7.2002	-13.8995	-2.7833
C	1.4921	-1.7989	3.8105	C	-8.1747	1.5684	1.0131	H	9.2388	-18.4439	1.0566
C	1.3538	-2.2329	6.2313	H	-4.7296	-6.5137	-3.5911	H	7.9643	-18.2927	-1.0751
N	1.9929	-2.6598	7.2516	H	-5.3862	-4.0941	-3.4206	H	7.4971	-16.0781	-2.1012
C	1.7637	-2.8608	8.4957	H	-6.3098	-6.8898	0.4482	H	10.6438	-13.0029	2.7793
C	2.838	-3.3767	9.2779	H	-7.244	-4.9244	1.6082	H	11.2198	-15.1862	3.9807
C	2.6265	-3.6107	10.6457	H	-9.0502	0.8008	3.4813	H	10.481	-17.3291	2.9919
C	1.383	-3.3414	11.2232	H	-9.0295	-1.5032	4.4205	Zn	9.0805	-12.4238	0.1157

Table S10. Cartesian coordinates (in Å) for the PM7 model of **1** with C_3 symmetry

C	-5.4141	-7.9606	-1.5247	C	-4.9872	2.7555	0.5633	C	4.6172	-19.1016	4.7931
C	-5.5183	-7.3007	-0.365	C	-4.8788	1.5634	-0.1516	H	6.6437	-11.2155	-0.8987
C	-6.0694	-7.9993	0.8088	N	-2.2734	2.4346	-2.5071	H	6.79	-12.6222	1.1982
N	-6.4458	-9.2189	0.7567	C	-1.5032	3.3995	-2.8465	H	4.5172	-14.5908	-2.6261
C	-6.3746	-9.8891	-0.345	C	-1.5284	4.7052	-2.1622	H	4.036	-16.5957	-1.2676
C	-5.9059	-9.3534	-1.484	C	-2.3824	4.8852	-1.1573	H	3.252	-20.8934	3.2524
C	-6.8141	-11.3193	-0.3063	H	-2.1537	-4.0166	-5.5473	H	2.6599	-20.6457	0.8485
N	-7.2059	-11.768	0.8242	H	-1.5351	-1.5859	-5.2815	H	3.3185	-18.601	-0.4
C	-7.6353	-12.8744	1.3063	H	-4.9686	-3.3185	-2.3044	H	6.1901	-16.095	4.9537
C	-7.9525	-12.8967	2.696	H	-5.0995	-1.1112	-1.2177	H	5.5925	-18.1187	6.3998
C	-8.4235	-14.0913	3.2628	H	-4.2696	4.7802	0.8108	H	4.3402	-19.9918	5.3813
C	-8.5773	-15.2339	2.4733	H	-5.7161	2.8395	1.387	Zn	5.6282	-15.4371	1.9319
C	-8.2644	-15.2029	1.1151	H	-5.5253	0.7133	0.1154	C	-3.0978	-8.5717	-5.9524
C	-7.7934	-14.0273	0.5318	H	-0.7979	3.2515	-3.6791	C	-2.2248	-8.16	-6.8801
N	-7.7967	-11.8164	3.3964	H	-0.8505	5.5122	-2.4834	C	-1.6258	-9.1507	-7.791
C	-8.0678	-11.7879	4.6474	H	-2.4375	5.8442	-0.6166	N	-1.909	-10.3943	-7.7211
C	-8.5701	-12.9701	5.3712	C	1.3975	-0.9514	-0.904	C	-2.7594	-10.833	-6.8543
C	-8.7476	-14.1048	4.6988	C	0.2359	-0.9599	-0.2388	C	-3.3815	-10.0221	-5.9833
H	-5.1772	-6.2633	-0.2394	C	-0.9767	-0.4579	-0.9071	C	-3.014	-12.3078	-6.8295
H	-6.1322	-7.4602	1.767	N	-0.9569	-0.0187	-2.1063	N	-2.3498	-13.0183	-7.6582
H	-5.8733	-9.9801	-2.3862	C	0.144	0.0227	-2.7802	C	-2.2379	-14.2562	-7.9681
H	-6.7827	-11.9526	-1.2058	C	1.3156	-0.3918	-2.2698	C	-1.3123	-14.5852	-9.0012
H	-8.9483	-16.1688	2.9248	C	0.0663	0.5307	-4.1862	C	-1.1588	-15.9311	-9.3686
H	-8.3895	-16.1108	0.5011	N	-1.0918	0.8622	-4.6134	C	-1.9043	-16.9255	-8.7301
H	-7.5498	-14.0174	-0.5416	C	-1.6099	1.3104	-5.6959	C	-2.8065	-16.5948	-7.72
H	-7.9223	-10.8487	5.2036	C	-3.0193	1.5255	-5.7004	C	-2.973	-15.2642	-7.3377
H	-8.7927	-12.9067	6.4485	C	-3.6254	2.013	-6.8688	N	-0.6384	-13.635	-9.5708
H	-9.1215	-15.0141	5.1975	C	-2.8554	2.2808	-8.0036	C	0.2049	-13.8808	-10.5023
C	-2.633	-10.9058	3.6967	C	-1.4778	2.0673	-7.9904	C	0.466	-15.2485	-10.9868
C	-3.0472	-11.7364	2.7324	C	-0.8553	1.5823	-6.8408	C	-0.1999	-16.2607	-10.4359
C	-4.4388	-11.6468	2.2595	N	-3.7009	1.2643	-4.6286	H	-1.9027	-7.1134	-6.9717
N	-5.2618	-10.7954	2.7376	C	-4.9682	1.4413	-4.5843	H	-0.8882	-8.811	-8.5348
C	-4.9113	-9.9911	3.6855	C	-5.733	1.9483	-5.7383	H	-4.0877	-10.4683	-5.2699
C	-3.6751	-9.9928	4.2101	C	-5.0813	2.2313	-6.8637	H	-3.7263	-12.7542	-6.1192
C	-5.9354	-9.0111	4.1659	H	0.1366	-1.3603	0.7799	H	-1.7805	-17.9806	-9.025
N	-7.0696	-9.0336	3.578	H	-1.9344	-0.4903	-0.3645	H	-3.3899	-17.3879	-7.2225
C	-8.1813	-8.4006	3.6419	H	2.2138	-0.3301	-2.8999	H	-3.6884	-15.0176	-6.5384
C	-9.2026	-8.7899	2.7269	H	0.9623	0.5963	-4.8217	H	0.7544	-13.0431	-10.9595
C	-10.4344	-8.1178	2.7629	H	-3.3379	2.6636	-8.918	H	1.1991	-15.4206	-11.7912
C	-10.649	-7.0874	3.6819	H	-0.8795	2.2825	-8.8918	H	-0.0389	-17.2999	-10.7664
C	-9.6441	-6.7131	4.5728	H	0.233	1.4177	-6.845	C	2.6979	-11.7505	-5.9882
C	-8.4123	-7.3662	4.5532	H	-5.5073	1.2081	-3.6528	C	1.6641	-12.4646	-5.5293
N	-8.9658	-9.7501	1.8879	H	-6.8233	2.0879	-5.662	C	0.4535	-12.5789	-6.3608
C	-9.8519	-10.1343	1.0472	H	-5.6108	2.6129	-7.7521	N	0.36	-12.0129	-7.5027
C	-11.1887	-9.5166	0.9746	Zn	-2.4487	0.6111	-3.2475	C	1.3439	-11.3337	-7.991
C	-11.4827	-8.5259	1.8133	C	2.6655	-1.5192	-0.3371	C	2.5034	-11.175	-7.3343
H	-2.3776	-12.4548	2.2387	C	3.7309	-1.867	-1.1775	C	1.1371	-10.6857	-9.3238
H	-4.7666	-12.3087	1.4427	C	4.9029	-2.4617	-0.6926	N	-0.0112	-10.8385	-9.8619
H	-3.4413	-9.2714	5.0055	C	4.9817	-2.7155	0.6819	C	-0.6114	-10.4706	-10.9319

H	-5.7101	-8.2959	4.9715	C	3.9202	-2.4423	1.5515	C	-1.9614	-10.8974	-11.0973
H	-11.619	-6.5639	3.705	C	2.7802	-1.8259	1.0241	C	-2.6548	-10.5121	-12.2553
H	-9.824	-5.8982	5.2943	H	5.8834	-3.1879	1.0881	C	-2.0269	-9.725	-13.2241
H	-7.6277	-7.0599	5.2619	H	1.9321	-1.6256	1.6897	C	-0.7061	-9.3124	-13.0538
H	-9.6095	-10.9549	0.3541	H	3.6152	-1.7218	-2.2577	C	0.001	-9.6825	-11.9105
H	-11.9251	-9.8747	0.2373	C	0.6404	-9.5281	5.09	N	-2.5107	-11.626	-10.1757
H	-12.4691	-8.034	1.7961	C	1.52	-10.5426	4.6942	C	-3.7203	-12.0327	-10.2796
Zn	-7.1249	-10.3974	2.1972	C	1.0795	-11.7148	4.0682	C	-4.5643	-11.7031	-11.4427
C	3.9568	-2.8915	2.9807	C	-0.292	-11.8465	3.8208	C	-4.0488	-10.9571	-12.4169
C	4.8141	-3.8211	3.4192	C	-1.2019	-10.8303	4.1362	H	1.6635	-12.9313	-4.5337
C	4.7003	-4.3028	4.8053	C	-0.7169	-9.69	4.7882	H	-0.4091	-13.1401	-5.969
N	3.803	-3.8649	5.6007	H	-0.6612	-12.7524	3.3245	H	3.2981	-10.5776	-7.8034
C	2.9676	-2.9538	5.2268	H	-1.4145	-8.877	5.0219	H	1.9315	-10.0927	-9.802
C	2.9851	-2.4258	3.9919	H	2.5966	-10.3864	4.8266	H	-2.5774	-9.4258	-14.1312
C	1.9382	-2.5197	6.2229	C	3.9175	-11.4695	-5.169	H	-0.2198	-8.6916	-13.8251
N	1.9578	-3.1046	7.359	C	4.6876	-10.3328	-5.4302	H	1.043	-9.3492	-11.7887
C	1.2945	-3.069	8.4542	C	5.7608	-9.9578	-4.6152	H	-4.1462	-12.6509	-9.474
C	1.7012	-3.9622	9.4884	C	6.0735	-10.7731	-3.5206	H	-5.6011	-12.0719	-11.4968
C	0.9985	-3.9553	10.7033	C	5.331	-11.924	-3.224	H	-4.6416	-10.6857	-13.3058
C	-0.0794	-3.0858	10.8892	C	4.257	-12.252	-4.0613	Zn	-1.1767	-11.9213	-8.7489
C	-0.4706	-2.2162	9.8721	H	6.8872	-10.4708	-2.8479	C	6.4774	-8.6642	-4.8479
C	0.2128	-2.2073	8.6567	H	3.6293	-13.1174	-3.8127	C	5.9254	-7.6609	-5.5398
N	2.7057	-4.7548	9.2787	H	4.4036	-9.6852	-6.2691	C	6.6494	-6.3852	-5.6505
C	3.1088	-5.5737	10.1766	C	-3.9447	-5.4129	-3.9673	N	7.7861	-6.2052	-5.0987
C	2.4633	-5.6722	11.4984	C	-3.5282	-6.2666	-4.9965	C	8.3628	-7.1486	-4.4308
C	1.4265	-4.8814	11.7648	C	-3.681	-7.6568	-4.9148	C	7.8056	-8.3622	-4.2747
H	5.5775	-4.2793	2.7748	C	-4.3089	-8.1787	-3.777	C	9.683	-6.8273	-3.8017
H	5.3893	-5.0881	5.1533	C	-4.7989	-7.3605	-2.753	N	10.1198	-5.6359	-3.9605
H	2.2374	-1.6621	3.7362	C	-4.5968	-5.9811	-2.8666	C	11.1399	-4.9456	-3.6083
H	1.1936	-1.7473	5.9772	H	-4.3686	-9.2664	-3.6523	C	11.1831	-3.5858	-4.0359
H	-0.6267	-3.0848	11.8461	H	-4.9161	-5.3316	-2.0432	C	12.2835	-2.7958	-3.668
H	-1.3228	-1.5336	10.0295	H	-3.0289	-5.8355	-5.8728	C	13.3144	-3.3383	-2.8965
H	-0.1071	-1.5156	7.8624	C	2.0644	-12.7361	3.5796	C	13.263	-4.6683	-2.4815
H	3.9677	-6.2267	9.9564	C	1.7431	-13.6953	2.7029	C	12.179	-5.4709	-2.835
H	2.8392	-6.3891	12.2459	C	2.8021	-14.5804	2.1889	N	10.2071	-3.1169	-4.7495
H	0.9128	-4.9194	12.7394	N	4.0211	-14.4588	2.5501	C	10.2009	-1.903	-5.1565
C	1.1639	-8.2642	5.7074	C	4.3725	-13.5625	3.4103	C	11.2925	-0.9626	-4.8438
C	0.4474	-7.1364	5.7903	C	3.4925	-12.7105	3.9606	C	12.3187	-1.3931	-4.1138
C	1.0769	-5.9265	6.3481	C	5.8258	-13.4793	3.7585	H	4.9294	-7.7318	-5.9998
N	2.2891	-5.9161	6.7514	N	6.6072	-14.2905	3.1552	H	6.1806	-5.5545	-6.2004
C	3.0119	-6.985	6.7022	C	7.86	-14.5562	3.1284	H	8.3418	-9.1236	-3.6902
C	2.5419	-8.1506	6.2296	C	8.2854	-15.5885	2.2421	H	10.2363	-7.5787	-3.2182
C	4.428	-6.8779	7.1742	C	9.6507	-15.909	2.183	H	14.1759	-2.7124	-2.6111
N	4.8138	-5.7269	7.5721	C	10.5707	-15.2252	2.982	H	14.0816	-5.0861	-1.8714
C	5.8862	-5.1923	8.0244	C	10.1458	-14.2158	3.8448	H	12.1512	-6.519	-2.4996
C	5.8357	-3.8026	8.3382	C	8.7945	-13.8799	3.9179	H	9.3522	-1.5479	-5.7617
C	6.9918	-3.1792	8.8331	N	7.4022	-16.2008	1.5164	H	11.2521	0.0751	-5.2119
C	8.1674	-3.9131	9.0118	C	7.7359	-17.1344	0.7062	H	13.1576	-0.7264	-3.8552
C	8.2076	-5.2717	8.7014	C	9.134	-17.5731	0.5435	C	5.986	-2.9193	-1.6277
C	7.0708	-5.9111	8.2087	C	10.0801	-16.977	1.2652	C	7.0069	-3.6917	-1.2339
N	4.7271	-3.1544	8.1569	H	0.7243	-13.8296	2.3136	C	7.9546	-4.1993	-2.2397

C	4.6337	-1.9057	8.4239	H	2.538	-15.3485	1.4452	N	7.834	-3.9262	-3.4808
C	5.7693	-1.1261	8.9498	H	3.8661	-11.9679	4.6787	C	6.8827	-3.1626	-3.9035
C	6.9305	-1.7438	9.1535	H	6.1993	-12.7424	4.4856	C	5.9661	-2.6311	-3.0777
H	-0.5884	-7.0629	5.4304	H	11.6414	-15.4832	2.9322	C	6.8123	-2.91	-5.3772
H	0.4903	-4.9956	6.3945	H	10.8807	-13.6823	4.4712	N	7.6877	-3.4951	-6.1014
H	3.215	-9.0189	6.2195	H	8.4731	-13.0812	4.6037	C	7.9838	-3.5815	-7.3447
H	5.1034	-7.7468	7.1612	H	6.9527	-17.6298	0.1113	C	9.0891	-4.4123	-7.692
H	9.0722	-3.417	9.3999	H	9.3821	-18.3814	-0.163	C	9.443	-4.536	-9.0447
H	9.1414	-5.8413	8.845	H	11.1385	-17.2728	1.1787	C	8.7193	-3.8564	-10.028
H	7.117	-6.984	7.9671	C	5.5798	-12.7233	-1.9823	C	7.6381	-3.0482	-9.6792
H	3.673	-1.3946	8.2557	C	6.2174	-12.229	-0.9152	C	7.2695	-2.9108	-8.3416
H	5.6506	-0.0524	9.1672	C	6.2958	-13.0447	0.3094	N	9.7338	-5.0309	-6.752
H	7.8071	-1.2019	9.5447	N	5.7702	-14.207	0.3849	C	10.7333	-5.7867	-7.0147
Zn	3.3828	-4.42	7.4537	C	5.1688	-14.731	-0.631	C	11.2146	-6.0051	-8.3911
C	-3.6141	-3.95	-3.9808	C	5.0505	-14.091	-1.8052	C	10.5864	-5.3951	-9.3935
C	-2.6901	-3.4233	-4.7934	C	4.5619	-16.0862	-0.4458	H	7.1544	-4.0041	-0.1912
C	-2.3209	-2.0063	-4.6345	N	4.6642	-16.6051	0.7171	H	8.7721	-4.861	-1.9133
N	-2.8521	-1.2643	-3.7408	C	4.2999	-17.6812	1.3086	H	5.1755	-2.0053	-3.513
C	-3.7667	-1.7241	-2.9538	C	4.6448	-17.8129	2.6856	H	6.0309	-2.2683	-5.8117
C	-4.1979	-2.9938	-3.0169	C	4.259	-18.9757	3.3707	H	9.0018	-3.9589	-11.0887
C	-4.3168	-0.7903	-1.9218	C	3.5516	-19.9825	2.7085	H	7.0726	-2.5173	-10.4636
N	-3.8151	0.384	-1.887	C	3.2188	-19.8449	1.3617	H	6.4126	-2.271	-8.0808
C	-3.9582	1.4541	-1.1977	C	3.5895	-18.697	0.6626	H	11.2532	-6.2911	-6.1853
C	-3.1234	2.5591	-1.5354	N	5.3011	-16.8568	3.2662	H	12.079	-6.6623	-8.5778
C	-3.2484	3.7492	-0.8016	C	5.6316	-16.9292	4.5011	H	10.9104	-5.5283	-10.4387
C	-4.1761	3.8421	0.2392	C	5.2909	-18.0926	5.3403	Zn	8.8955	-4.5679	-5.0241

Table S11. Cartesian coordinates (in Å) for the PM7 model of **1** with S_4 symmetry

Zn	13.0405	31.5482	-0.3011	H	21.345	31.4416	10.7153	H	8.3467	21.411	9.4867
Zn	19.3702	21.0018	1.2505	C	27.2558	32.3177	-3.3551	C	10.2739	22.167	10.1265
Zn	27.1631	31.5481	-0.3982	C	27.1288	33.1903	-4.3622	H	10.8325	21.3775	9.5976
Zn	20.5862	34.4751	9.9188	H	27.2196	32.8594	-5.4106	C	10.9503	23.142	10.8602
N	11.0452	31.575	-0.2905	C	26.8673	34.607	-4.0512	C	10.2166	24.1267	11.5398
N	12.7838	29.7486	-1.0976	H	26.7597	35.344	-4.8634	C	13.4082	22.6578	10.3762
N	15.0007	31.1465	-0.436	C	26.7562	35.005	-2.6709	H	13.258	21.902	9.5893
N	18.5253	19.2154	1.5314	C	26.5096	36.3235	-2.2853	C	14.7362	23.2048	10.7216
N	17.5497	21.5136	0.6514	H	26.3919	37.1106	-3.0479	C	15.9725	22.8209	10.0208
N	19.8519	22.9056	0.8712	C	26.4134	36.651	-0.9318	H	15.9156	22.0455	9.2412
N	29.1555	31.5357	-0.3692	H	26.2225	37.6952	-0.6326	C	17.129	23.4409	10.3072
N	27.4416	29.8575	-1.3914	C	26.5603	35.6647	0.0445	C	17.1056	24.4743	11.3682
N	25.2065	31.176	-0.6069	H	26.4876	35.9389	1.1097	H	18.0288	25.018	11.6276
C	10.1824	32.5516	0.1496	C	26.8009	34.3426	-0.3313	C	15.9608	24.7768	11.9999
C	8.7234	32.3364	0.1559	C	26.9045	34.0196	-1.6925	H	15.9589	25.5453	12.7891
H	8.0452	33.1323	0.5074	C	26.7062	33.0803	1.8745	C	20.7821	22.9285	9.3236
C	8.2376	31.1656	-0.2711	H	26.4188	33.9281	2.5161	C	20.6149	22.6602	7.9604
H	7.1496	30.9888	-0.2738	C	26.7387	31.6851	2.356	H	21.5091	22.5359	7.3347
C	9.1616	30.107	-0.7374	C	26.3565	31.3031	3.7241	C	19.3569	22.5699	7.3643
C	8.6998	28.8692	-1.1887	H	26.0987	32.1034	4.4342	C	18.2432	22.8284	8.1643
H	7.6185	28.6564	-1.2065	C	26.298	30.0078	4.0743	H	17.2423	22.7931	7.7122
C	9.6028	27.8966	-1.6215	C	26.6687	29.0096	3.0437	C	18.3644	23.1478	9.5234
H	9.2294	26.9228	-1.9805	H	26.6278	27.9335	3.278	C	19.6442	23.1932	10.0957
C	10.9746	28.1504	-1.6044	C	27.0382	29.4033	1.814	H	19.7545	23.4123	11.169
H	11.6758	27.3751	-1.9539	H	27.3181	28.6491	1.0616	N	21.9313	35.19	11.2094
C	11.4487	29.3814	-1.1502	C	26.7414	20.5633	12.6701	N	19.8665	33.5586	11.5249
C	10.5364	30.3589	-0.7245	C	26.6305	19.8837	13.9742	N	19.1206	33.5383	8.9299
C	13.9788	29.082	-1.1774	H	26.5769	18.7831	14.0252	N	12.9069	23.7548	13.8282
H	13.9921	28.0198	-1.4689	C	26.5981	20.6208	15.0899	N	13.3734	26.3005	13.2723
C	15.197	29.8284	-0.7967	H	26.5167	20.1286	16.0728	N	13.0091	26.0788	10.5964
C	16.4053	29.2562	-0.756	C	26.6719	22.0969	15.004	N	13.0847	32.4954	-2.0563
H	16.4971	28.2034	-1.0646	C	26.6451	22.9055	16.1417	N	13.2283	33.3808	0.4387
C	17.601	29.9948	-0.2874	H	26.5673	22.449	17.1418	N	13.0758	30.9528	1.6112
C	17.4346	31.2789	0.0681	C	26.7217	24.294	16.0199	C	22.9881	36.0429	10.9708
H	18.2784	31.8852	0.4348	H	26.7067	24.9256	16.9239	C	23.9049	36.3264	11.9039
C	16.0873	31.8787	-0.013	C	26.8212	24.887	14.7609	H	24.7392	37.0141	11.6848
H	15.9303	32.9311	0.2701	H	26.889	25.9843	14.68	C	23.7832	35.7074	13.2359
C	19.0805	18.0415	1.9853	C	26.8401	24.09	13.616	H	24.5262	35.9252	14.0201
C	18.2716	16.8164	2.1236	C	26.774	22.6944	13.7445	C	22.6792	34.8166	13.489
H	18.7332	15.8836	2.4892	C	26.7696	25.7858	11.7208	C	22.488	34.1847	14.7186
C	16.9748	16.8547	1.7985	H	26.6199	26.6826	12.3429	H	23.1946	34.359	15.5463
H	16.3562	15.9475	1.8952	C	26.7003	25.8089	10.2442	C	21.4023	33.3269	14.904
C	16.3718	18.1141	1.3064	C	26.416	26.921	9.5576	H	21.2568	32.8293	15.8775
C	15.0243	18.199	0.9516	H	26.2772	27.8629	10.1112	C	20.5026	33.0923	13.8632
H	14.375	17.3123	1.0344	C	26.2478	26.8998	8.0867	H	19.6522	32.4094	14.0227
C	14.4928	19.4042	0.4894	C	26.4281	25.7282	7.4564	C	20.6855	33.7192	12.6302
H	13.4274	19.4623	0.2105	H	26.3047	25.6425	6.3647	C	21.7742	34.5859	12.4509
C	15.3023	20.5351	0.3765	C	26.7626	24.5291	8.2505	C	18.8115	32.7478	11.1933
H	14.8701	21.4798	0.0083	H	26.9143	23.5575	7.7553	H	18.3275	32.1373	11.9721

C	16.6504	20.4628	0.7277	C	24.4744	30.1775	7.4023	C	18.4038	32.7139	9.7724
C	17.1797	19.2492	1.1931	C	24.9105	30.4379	6.1014	C	17.4207	31.9266	9.3206
C	17.5063	22.8355	0.2886	H	24.5047	31.3075	5.5685	H	16.8596	31.3324	10.0564
H	16.5543	23.2784	-0.0446	C	25.8331	29.6188	5.4398	C	17.1013	31.8304	7.8752
C	18.7542	23.6163	0.4304	C	26.2707	28.4542	6.0824	C	17.8494	32.5676	7.035
C	18.8212	24.9284	0.1756	H	26.9959	27.7945	5.582	H	17.7104	32.5305	5.9438
H	17.922	25.4274	-0.2153	C	25.822	28.1363	7.371	C	18.9026	33.4458	7.5773
C	20.0567	25.7072	0.4322	C	24.9391	29.0118	8.0144	H	19.5241	34.0522	6.9004
C	21.1112	25.0398	0.9328	H	24.5956	28.7611	9.0272	C	12.6578	22.423	14.0658
H	22.0533	25.5489	1.1885	Zn	26.8562	23.111	10.973	C	12.8419	21.838	15.4069
C	21.006	23.588	1.1691	Zn	12.8872	24.767	12.1084	H	12.6326	20.769	15.5809
H	21.8579	23.0275	1.5843	N	28.8368	22.9017	10.8236	C	13.2629	22.6222	16.4052
C	29.9936	32.4834	0.1705	N	26.8379	21.6037	9.6809	H	13.4047	22.201	17.414
C	31.3163	32.3167	0.1306	N	24.8543	23.0461	10.8341	C	13.5385	24.0551	16.1548
H	32.0012	33.0685	0.5583	N	19.999	20.4361	-0.5573	C	13.9813	24.9101	17.1656
C	31.8399	31.094	-0.5051	N	21.1848	20.4274	1.8091	H	14.1351	24.5247	18.1867
H	32.9315	30.9443	-0.5455	N	19.1058	21.4769	3.1752	C	14.2291	26.256	16.8901
C	30.9997	30.1829	-1.0255	N	10.9219	25.0708	12.2722	H	14.5749	26.9258	17.6953
C	31.4838	29.0766	-1.618	N	12.3263	23.2084	11.0137	C	14.0397	26.7588	15.6025
H	32.5716	28.9014	-1.6687	N	14.758	24.1741	11.7007	H	14.2342	27.8248	15.4005
C	30.6605	28.1735	-2.1614	C	29.844	23.612	11.4348	C	13.6037	25.9128	14.5824
H	31.0567	27.2698	-2.6543	C	31.2615	23.3625	11.1125	C	13.3478	24.5628	14.8666
C	29.2098	28.4108	-2.0946	H	32.0562	23.9379	11.6167	C	13.7181	27.387	12.5116
H	28.5122	27.6856	-2.5447	C	31.5673	22.4303	10.2034	H	14.16	28.276	12.9884
C	28.7757	29.5205	-1.4856	H	32.6218	22.2307	9.9517	C	13.5493	27.2617	11.0502
C	29.6658	30.3939	-0.9616	C	30.489	21.6693	9.5337	C	13.9883	28.298	10.103
C	26.2505	29.1888	-1.5232	C	30.7603	20.6909	8.5752	H	14.401	29.2334	10.5103
H	26.2393	28.1466	-1.8793	H	31.8025	20.4638	8.2973	C	13.9103	28.0827	8.7795
C	25.0325	29.8879	-1.064	C	29.7157	19.9943	7.9651	C	13.3349	26.7912	8.3354
C	23.7094	29.2451	-1.0039	H	29.9383	19.2205	7.2113	H	13.2578	26.5623	7.26
H	23.6075	28.214	-1.3759	C	28.3913	20.2709	8.3053	C	12.9212	25.8832	9.2343
C	22.6682	29.9007	-0.4663	H	27.5761	19.7105	7.8193	H	12.489	24.9309	8.8883
C	22.9037	31.2817	0.0158	C	28.1082	21.2501	9.258	C	13.0114	31.9857	-3.3318
H	22.0816	31.8523	0.478	C	29.1611	21.9419	9.8742	C	13.1803	32.8532	-4.5123
C	24.1202	31.8391	-0.0796	C	25.5673	21.3743	9.2253	H	13.1133	32.4292	-5.5285
H	24.2802	32.8682	0.2788	H	25.3958	20.6638	8.4016	C	13.4132	34.1578	-4.3311
C	18.9131	29.2892	-0.1947	C	24.4864	22.175	9.8322	H	13.5406	34.8205	-5.2027
C	18.9199	27.9228	0.104	C	23.0945	22.1135	9.3633	C	13.5013	34.7106	-2.9604
H	17.9642	27.428	0.324	H	22.8442	21.3963	8.5663	C	13.7444	36.0648	-2.7243
C	20.0988	27.1767	0.159	C	22.1684	22.937	9.8789	H	13.8761	36.7579	-3.5711
C	21.3043	27.8541	-0.0353	C	22.6036	23.8587	10.9546	C	13.8186	36.5484	-1.4169
H	22.2482	27.2953	0.0301	H	21.8804	24.5581	11.4048	H	14.0058	37.6207	-1.239
C	21.3466	29.2295	-0.297	C	23.882	23.8649	11.371	C	13.6544	35.6837	-0.3343
C	20.1404	29.9373	-0.381	H	24.191	24.5532	12.1734	H	13.7089	36.0811	0.6924
H	20.1585	31.0113	-0.6213	C	19.3369	20.4511	-1.7628	C	13.4186	34.3266	-0.5567
N	19.4502	36.1014	10.1324	C	19.9876	19.9778	-2.9984	C	13.3344	33.8478	-1.8734
N	21.308	35.4657	8.3583	H	19.4417	19.9971	-3.9568	C	13.5227	33.3236	1.7769
N	21.8394	33.0169	9.3665	C	21.2449	19.5256	-2.9377	H	13.8445	34.2359	2.304
N	27.1533	32.6955	-2.0329	H	21.7456	19.1674	-3.852	C	13.4714	31.9955	2.4252
N	26.9731	33.2847	0.546	C	21.9621	19.5038	-1.6426	C	13.8461	31.7989	3.6945
N	27.0878	30.7269	1.4302	C	23.2729	19.0367	-1.5315	H	14.158	32.6684	4.294

N	26.8137	21.9342	12.5842	H	23.8058	18.6703	-2.4241	C	13.8844	30.4425	4.2882
N	26.9503	24.5663	12.3196	C	23.9149	19.0297	-0.2918	C	13.4951	29.4144	3.5178
N	26.8802	24.59	9.6205	H	24.9504	18.6585	-0.2129	H	13.5127	28.3789	3.8945
C	18.4807	36.3756	11.0687	C	23.253	19.4877	0.848	C	13.0688	29.6781	2.1295
C	17.6249	37.5712	10.9558	H	23.771	19.4744	1.8208	H	12.7443	28.8504	1.4799
H	16.8558	37.7788	11.7188	C	21.9428	19.9565	0.7498	C	16.0007	30.9363	7.3936
C	17.7977	38.3975	9.9183	C	21.3027	19.9635	-0.499	C	15.4988	29.9193	8.2087
H	17.1665	39.2958	9.8189	C	21.3655	20.6153	3.1555	H	15.9679	29.7392	9.1846
C	18.8333	38.1032	8.902	H	22.335	20.3662	3.6155	C	14.4298	29.1034	7.8201
C	19.0412	38.9308	7.7972	C	20.2444	21.2164	3.91	C	13.9002	29.2679	6.5347
H	18.4278	39.8367	7.6631	C	20.3258	21.5171	5.2112	H	13.0588	28.6381	6.2076
C	20.0227	38.615	6.8562	H	21.2456	21.2436	5.7492	C	14.4069	30.2478	5.6709
H	20.1797	39.2738	5.9857	C	19.2282	22.2233	5.9156	C	15.4428	31.075	6.121
C	20.8025	37.4678	7.0078	C	18.1421	22.5475	5.1928	H	15.8323	31.8462	5.4427
H	21.5704	37.2281	6.2543	H	17.2987	23.1049	5.6289	H	10.6033	33.5095	0.4929
C	20.6039	36.6323	8.1072	C	18.0784	22.1682	3.7692	H	20.1502	18.0313	2.2456
C	19.6216	36.9592	9.0549	H	17.1992	22.4315	3.1609	H	29.5545	33.3814	0.6331
C	22.2228	34.7037	7.6777	C	10.2371	26.0472	12.9579	H	18.3613	35.6864	11.9191
H	22.6857	35.0989	6.7595	C	8.7641	26.1104	12.9308	H	27.4578	31.2573	-3.5735
C	22.5102	33.3539	8.2091	H	8.2304	26.8992	13.4876	H	26.775	19.9752	11.7398
C	23.3475	32.5019	7.606	C	8.0883	25.1979	12.2239	H	29.5713	24.3723	12.1832
H	23.8851	32.8551	6.7141	H	6.9868	25.2294	12.1947	H	18.302	20.826	-1.7902
C	23.5373	31.1156	8.0989	C	8.8198	24.1437	11.485	H	10.8127	26.7872	13.5355
C	22.8244	30.7494	9.1791	C	8.1553	23.1662	10.7424	H	23.0611	36.5058	9.9741
H	22.871	29.7302	9.5918	H	7.0542	23.1649	10.6919	H	12.3057	21.7967	13.2314
C	21.9365	31.7289	9.8323	C	8.8799	22.1832	10.0662	H	12.8133	30.9098	-3.4579

Table S12. Cartesian coordinates (in Å) for the PM7 model of **1** with D_2 symmetry

C	1.4947	-8.7347	-7.603	C	-10.866	-7.2117	4.3695	H	1.5131	-6.0265	-7.5407
C	0.1312	-8.4285	-7.546	C	-9.8669	-7.5891	3.4515	H	4.9259	-5.5782	-4.9514
C	-0.8002	-9.2882	-8.1473	N	-8.7459	-4.2903	2.2351	H	3.7174	-9.4997	-6.2605
N	-0.4367	-10.429	-8.7572	C	-9.0047	-3.0006	2.4587	C	3.477	-10.714	3.8355
C	0.8906	-10.72	-8.8376	C	-9.9763	-2.5586	3.392	C	4.4187	-10.694	2.8012
C	1.8789	-9.8954	-8.288	C	-10.703	-3.4819	4.1033	C	5.2785	-9.6005	2.6425
C	1.2251	-11.984	-9.5457	H	-4.1671	-7.7713	-2.5223	C	5.1689	-8.5096	3.513
N	0.2295	-12.758	-9.8294	H	-5.5756	-5.8079	-1.8551	C	4.2191	-8.5075	4.5406
C	0.2973	-13.98	-10.512	H	-6.1572	-10.096	0.521	C	3.4004	-9.6289	4.7163
C	-0.9625	-14.68	-10.582	H	-7.9276	-8.9734	1.8981	H	5.8374	-7.6541	3.3931
C	-1.0339	-15.918	-11.262	H	-11.941	-5.5966	5.3169	H	2.7055	-9.6663	5.5615
C	0.1329	-16.437	-11.885	H	-11.426	-7.9975	4.9026	H	4.478	-11.535	2.1092
C	1.3235	-15.749	-11.818	H	-9.6751	-8.6539	3.2941	C	0.6935	-0.9029	-1.2221
C	1.4146	-14.524	-11.128	H	-8.4282	-2.2663	1.8781	C	2.0357	-1.1388	-1.5349
N	-2.0702	-14.102	-9.9871	H	-10.142	-1.4804	3.524	C	2.5776	-0.6095	-2.7161
C	-3.2283	-14.763	-10.038	H	-11.481	-3.1689	4.8212	N	1.8451	0.1043	-3.5877
C	-3.3726	-16.015	-10.686	C	-3.0696	-3.2049	1.1076	C	0.5434	0.356	-3.2772
C	-2.2884	-16.587	-11.305	C	-3.3597	-4.3815	1.8048	C	-0.0601	-0.1212	-2.1083
H	-0.2191	-7.5291	-7.0444	C	-4.657	-4.9154	1.7617	C	-0.2	1.1802	-4.2647
H	-1.8751	-9.0536	-8.1472	N	-5.6453	-4.3556	1.0429	N	0.4054	1.4167	-5.3821
H	2.935	-10.145	-8.4075	C	-5.3708	-3.2047	0.3683	C	-0.0831	2.1967	-6.4395
H	2.2832	-12.162	-9.7769	C	-4.1102	-2.5992	0.3879	C	0.7652	2.1954	-7.6062
H	0.0694	-17.397	-12.429	C	-6.5041	-2.6224	-0.3949	C	0.3969	2.9648	-8.7344
H	2.2223	-16.151	-12.314	N	-7.5756	-3.3417	-0.4657	C	-0.791	3.7429	-8.6878
H	2.3779	-14.007	-11.103	C	-8.7748	-2.9754	-1.0933	C	-1.5823	3.7401	-7.5609
H	-4.0953	-14.292	-9.5511	C	-9.7684	-4.0205	-1.107	C	-1.2379	2.9657	-6.4364
H	-4.3562	-16.506	-10.693	C	-11.036	-3.7673	-1.6817	N	1.9278	1.4458	-7.573
H	-2.3745	-17.552	-11.836	C	-11.313	-2.4802	-2.2162	C	2.6983	1.4329	-8.6624
C	-2.8838	-11.897	-4.5533	C	-10.354	-1.4925	-2.1861	C	2.3851	2.1631	-9.8368
C	-1.6107	-12.357	-4.9051	C	-9.0823	-1.7345	-1.6318	C	1.2495	2.9347	-9.873
C	-1.2858	-12.537	-6.258	N	-9.4463	-5.2399	-0.5377	H	2.6683	-1.7238	-0.8704
N	-2.1473	-12.25	-7.2486	C	-10.362	-6.2096	-0.5746	H	3.6379	-0.754	-2.9706
C	-3.3958	-11.825	-6.9121	C	-11.647	-6.0364	-1.1477	H	-1.0967	0.1297	-1.8791
C	-3.8012	-11.651	-5.5837	C	-11.991	-4.8212	-1.6873	H	-1.1969	1.5322	-3.9718
C	-4.3057	-11.549	-8.0542	H	-2.5915	-4.8879	2.386	H	-1.0667	4.3558	-9.5655
N	-3.7642	-11.553	-9.2279	H	-4.9171	-5.8212	2.3284	H	-2.4962	4.3557	-7.5265
C	-4.425	-11.346	-10.447	H	-3.9429	-1.6558	-0.1344	H	-1.8895	2.9963	-5.5591
C	-3.5437	-11.298	-11.588	H	-6.3515	-1.6287	-0.8348	H	3.6134	0.8242	-8.6212
C	-4.0885	-11.115	-12.88	H	-12.31	-2.2811	-2.6495	H	3.064	2.1087	-10.7
C	-5.4969	-11.004	-13.032	H	-10.579	-0.4925	-2.5927	H	0.9898	3.5308	-10.766
C	-6.3229	-11.063	-11.932	H	-8.3518	-0.9211	-1.6213	C	2.9476	-4.1925	-6.1758
C	-5.7943	-11.228	-10.637	H	-10.085	-7.1768	-0.1312	C	1.6521	-3.6928	-6.0116
N	-2.1845	-11.448	-11.377	H	-12.355	-6.8766	-1.141	C	1.4448	-2.3065	-5.9498
C	-1.3725	-11.384	-12.434	H	-12.992	-4.6492	-2.1212	N	2.4556	-1.4235	-6.0167
C	-1.8438	-11.188	-13.756	Zn	-7.7158	-5.1409	0.6297	C	3.7167	-1.9037	-6.1985

C	-3.1926	-11.065	-13.984	C	6.2856	-9.592	1.5661	C	3.998	-3.2717	-6.2897
H	-0.8677	-12.582	-4.1432	C	6.567	-8.4238	0.8512	C	4.7827	-0.8736	-6.2999
H	-0.304	-12.93	-6.5608	C	7.5327	-8.445	-0.167	N	4.4304	0.3402	-6.0295
H	-4.8239	-11.345	-5.3544	N	8.1909	-9.5642	-0.5149	C	5.2506	1.4753	-6.0954
H	-5.3624	-11.368	-7.8186	C	7.9265	-10.704	0.1828	C	4.6132	2.6835	-5.6316
H	-5.9196	-10.877	-14.045	C	6.9984	-10.752	1.2282	C	5.326	3.9045	-5.6702
H	-7.4157	-10.988	-12.057	C	8.6927	-11.905	-0.2363	C	6.6491	3.9214	-6.1878
H	-6.4853	-11.275	-9.7908	N	9.4077	-11.783	-1.3063	C	7.2363	2.7598	-6.6373
H	-0.2947	-11.496	-12.245	C	10.238	-12.768	-1.8592	C	6.5459	1.5335	-6.5888
H	-1.1208	-11.148	-14.583	C	10.844	-12.385	-3.1111	N	3.3086	2.6058	-5.1769
H	-3.5912	-10.931	-15.005	C	11.731	-13.279	-3.7561	C	2.7308	3.7229	-4.731
Zn	-1.747	-12.12	-9.4638	C	12.024	-14.529	-3.1476	C	3.3885	4.979	-4.7199
C	4.0509	-7.3218	5.4007	C	11.442	-14.873	-1.9484	C	4.6727	5.0764	-5.1969
C	2.7705	-6.9061	5.7804	C	10.545	-14.001	-1.3019	H	0.8005	-4.366	-5.9372
C	2.6178	-5.7517	6.5629	N	10.542	-11.14	-3.6324	H	0.4331	-1.8862	-5.8521
N	3.6627	-4.9965	6.9419	C	11.089	-10.801	-4.8017	H	5.0175	-3.6161	-6.4683
C	4.9131	-5.4083	6.5952	C	11.973	-11.649	-5.5148	H	5.7801	-1.2153	-6.603
C	5.1449	-6.5653	5.8422	C	12.305	-12.875	-4.9934	H	7.1969	4.8805	-6.2318
C	6.024	-4.5404	7.0662	H	6.0482	-7.4943	1.0776	H	8.2583	2.7818	-7.05
N	5.6806	-3.4042	7.5779	H	7.7943	-7.5316	-0.7205	H	7.0452	0.6351	-6.9616
C	6.5463	-2.4379	8.1095	H	6.8452	-11.676	1.7879	H	1.6969	3.6402	-4.365
C	5.8856	-1.2189	8.5086	H	8.6004	-12.8	0.3917	H	2.8542	5.8608	-4.3389
C	6.6446	-0.1719	9.0816	H	12.731	-15.217	-3.6467	H	5.2031	6.0448	-5.2214
C	8.0414	-0.3497	9.2726	H	11.682	-15.84	-1.476	Zn	2.4042	0.7975	-5.656
C	8.6522	-1.5247	8.8956	H	10.11	-14.31	-0.3478	C	-2.4172	-13.146	0.2422
C	7.9129	-2.5699	8.3089	H	10.828	-9.8135	-5.2087	C	-1.1132	-13.634	0.1159
N	4.5171	-1.1228	8.3285	H	12.391	-11.306	-6.4716	C	-0.5806	-14.461	1.1168
C	3.9138	0.0135	8.6828	H	13.008	-13.549	-5.5149	N	-1.2699	-14.787	2.2232
C	4.6106	1.1116	9.2478	C	5.5959	-8.1991	-4.8209	C	-2.5476	-14.332	2.3415
C	5.9648	1.0197	9.4579	C	5.4594	-9.4053	-4.1272	C	-3.1532	-13.522	1.3741
H	1.8887	-7.4683	5.4812	C	6.5638	-9.9516	-3.4551	C	-3.2651	-14.758	3.5708
H	1.6253	-5.4221	6.9044	N	7.7616	-9.3426	-3.4201	N	-2.5535	-15.357	4.4681
H	6.1652	-6.8825	5.6177	C	7.9026	-8.171	-4.1	C	-3.0205	-15.879	5.6824
H	7.0444	-4.9276	6.9495	C	6.8552	-7.5816	-4.8156	C	-1.9782	-16.399	6.5336
H	8.6283	0.4649	9.7345	C	9.2513	-7.5519	-4.0383	C	-2.321	-16.98	7.7768
H	9.7341	-1.6612	9.058	N	10.098	-8.1033	-3.2319	C	-3.6886	-17.059	8.1539
H	8.4353	-3.4889	8.0289	C	11.433	-7.7163	-3.0465	C	-4.6703	-16.567	7.3232
H	2.8272	0.0746	8.5231	C	12.115	-8.4524	-2.0109	C	-4.3436	-15.972	6.0896
H	4.0523	2.0183	9.5203	C	13.478	-8.1809	-1.7481	N	-0.6694	-16.325	6.0907
H	6.5313	1.8504	9.9143	C	14.155	-7.2015	-2.524	C	0.2919	-16.791	6.8905
C	1.1315	-2.1511	3.5773	C	13.488	-6.5155	-3.5145	C	0.0273	-17.37	8.1575
C	2.5032	-2.4103	3.4903	C	12.127	-6.7634	-3.7772	C	-1.2697	-17.477	8.5965
C	3.2635	-2.5328	4.6632	N	11.408	-9.4185	-1.3173	H	-0.5072	-13.387	-0.7534
N	2.7165	-2.4435	5.8872	C	12.04	-10.082	-0.3471	H	0.4304	-14.885	1.027
C	1.3865	-2.168	5.9787	C	13.399	-9.8538	-0.0149	H	-4.1907	-13.205	1.4888
C	0.5709	-2.0027	4.8531	C	14.121	-8.9195	-0.7164	H	-4.3395	-14.542	3.622
C	0.8505	-2.0598	7.3605	H	4.5063	-9.93	-4.1054	H	-3.9538	-17.529	9.119

N	1.6463	-2.4273	8.3106	H	6.4894	-10.915	-2.9309	H	-5.7305	-16.643	7.6157
C	1.3696	-2.3902	9.6845	H	7.021	-6.6625	-5.3802	H	-5.153	-15.6	5.4558
C	2.4066	-2.9676	10.505	H	9.4308	-6.6816	-4.6822	H	1.3269	-16.714	6.5261
C	2.2588	-2.9727	11.911	H	15.224	-7.0036	-2.3264	H	0.8664	-17.735	8.7662
C	1.1	-2.3891	12.491	H	14.022	-5.7648	-4.1209	H	-1.509	-17.942	9.569
C	0.1258	-1.8337	11.692	H	11.637	-6.2017	-4.5769	C	2.5489	-11.85	3.985
C	0.2509	-1.8356	10.289	H	11.46	-10.837	0.203	C	1.1979	-11.622	4.2649
N	3.5261	-3.4883	9.8803	H	13.859	-10.439	0.7936	C	0.3204	-12.71	4.3865
C	4.4733	-4.0384	10.643	H	15.187	-8.7349	-0.4954	N	0.7219	-13.981	4.2169
C	4.3874	-4.0952	12.057	Zn	9.5985	-9.8998	-2.2414	C	2.0403	-14.21	3.9648
C	3.2948	-3.5573	12.692	C	-2.9824	-12.26	-0.7921	C	2.9777	-13.177	3.8507
H	2.9921	-2.5146	2.5242	C	-2.7579	-12.534	-2.1462	C	2.4232	-15.639	3.8228
H	4.3497	-2.7021	4.6229	C	-3.2213	-11.661	-3.1374	N	1.4498	-16.488	3.7752
H	-0.4836	-1.7442	4.969	C	-3.9736	-10.542	-2.7649	C	1.5694	-17.882	3.6828
H	-0.1717	-1.6752	7.4712	C	-4.2325	-10.271	-1.416	C	0.3084	-18.567	3.5371
H	0.9956	-2.3814	13.591	C	-3.7218	-11.128	-0.4337	C	0.2895	-19.979	3.4595
H	-0.7655	-1.3721	12.148	H	-4.37	-9.8761	-3.5347	C	1.5122	-20.697	3.5492
H	-0.5431	-1.3795	9.6915	H	-3.8953	-10.908	0.6202	C	2.705	-20.026	3.6997
H	5.351	-4.4558	10.127	H	-2.2309	-13.45	-2.4329	C	2.7418	-18.619	3.7615
H	5.2046	-4.5606	12.626	C	-1.7177	-2.618	1.1338	N	-0.8499	-17.811	3.4947
H	3.2088	-3.5686	13.792	C	-1.1687	-2.0522	-0.023	C	-2.0115	-18.449	3.3409
Zn	3.6434	-2.9498	7.8802	C	0.1041	-1.472	0.0035	C	-2.1079	-19.86	3.2369
C	-5.0301	-9.0911	-1.0363	C	0.8289	-1.4603	1.2007	C	-0.9692	-20.625	3.3074
C	-4.8779	-7.8725	-1.7046	C	0.3144	-2.0663	2.3527	H	0.8186	-10.611	4.3962
C	-5.654	-6.767	-1.3231	C	-0.9675	-2.626	2.3158	H	-0.7401	-12.561	4.6373
N	-6.5319	-6.8168	-0.3064	H	1.7997	-0.9557	1.2406	H	4.0314	-13.403	3.6809
C	-6.6895	-8.0033	0.3433	H	-1.3914	-3.0671	3.2207	H	3.4953	-15.867	3.7782
C	-5.9721	-9.1541	0.0008	H	-1.7345	-2.0693	-0.9552	H	1.4916	-21.801	3.5043
C	-7.6831	-8.0033	1.4471	C	4.4556	-7.6026	-5.5397	H	3.649	-20.589	3.7815
N	-8.1774	-6.8543	1.7729	C	3.562	-8.4187	-6.2435	H	3.7086	-18.125	3.8881
C	-9.1571	-6.6256	2.7497	C	2.4783	-7.8638	-6.9332	H	-2.9207	-17.832	3.3001
C	-9.448	-5.2303	2.9688	C	2.3153	-6.4738	-6.9448	H	-3.0972	-20.322	3.1121
C	-10.444	-4.8665	3.905	C	3.1823	-5.6474	-6.2208	H	-1.0143	-21.727	3.2508
C	-11.153	-5.8846	4.5979	C	4.2511	-6.2179	-5.5213	Zn	-0.5368	-15.84	4.0739

Table S13. Cartesian coordinates (in Å) for the PM7 model of **1** with *T* symmetry

C	2.7607	-2.4584	3.8514	C	3.5273	-8.409	-12.4125	H	-3.0611	2.4544	-0.8538
C	2.9789	-1.6668	2.7944	C	4.0059	-8.0352	-11.1572	H	-8.336	-1.4021	-1.5526
C	4.3528	-1.2253	2.5011	N	2.4372	-4.7958	-10.7884	H	-9.4321	0.1564	-3.2591
N	5.3463	-1.5785	3.2218	C	1.6638	-3.9745	-11.3938	H	-8.2596	2.2336	-3.9108
C	5.1808	-2.3256	4.2617	C	1.1021	-4.2597	-12.7266	C	-3.9512	-4.2368	-3.2835
C	3.9721	-2.7756	4.6375	C	1.4107	-5.4115	-13.3177	C	-3.303	-3.0744	-3.1394
C	6.4066	-2.7074	5.0318	H	5.0862	-3.6553	-4.2752	C	-3.796	-2.095	-2.157
N	7.5235	-2.2779	4.5824	H	3.8252	-3.0869	-6.3781	N	-4.8233	-2.3209	-1.4334
C	8.7649	-2.3628	4.8877	H	6.1494	-7.36	-6.295	C	-5.493	-3.4181	-1.5523
C	9.6906	-1.689	4.0376	H	5.2798	-7.8339	-8.5662	C	-5.147	-4.3791	-2.4251
C	11.06	-1.7597	4.3375	H	2.3094	-7.8624	-14.1139	C	-6.6718	-3.6006	-0.6474
C	11.5018	-2.4818	5.449	H	3.8183	-9.3809	-12.8457	N	-6.9061	-2.6606	0.1868
C	10.5899	-3.14	6.2731	H	4.6729	-8.7176	-10.6084	C	-7.7498	-2.4049	1.1165
C	9.2248	-3.0814	5.9948	H	1.4094	-3.0214	-10.9043	C	-7.5931	-1.1715	1.8149
N	9.2437	-1.0319	3.0131	H	0.4406	-3.5274	-13.2169	C	-8.4969	-0.855	2.8412
C	10.0349	-0.4095	2.2219	H	1.0146	-5.6679	-14.314	C	-9.5271	-1.7396	3.1704
C	11.496	-0.3983	2.4185	C	-0.6552	-5.9154	-6.7092	C	-9.6706	-2.9452	2.4851
C	12.0073	-1.0589	3.4546	C	0.1133	-6.9062	-7.178	C	-8.7856	-3.2782	1.4602
H	2.1797	-1.3598	2.1053	C	1.3361	-6.5722	-7.9269	N	-6.6223	-0.378	1.4846
H	4.5307	-0.5967	1.6147	N	1.6904	-5.3642	-8.1403	C	-6.4419	0.7409	2.0802
H	3.9072	-3.4135	5.5297	C	0.9706	-4.3765	-7.7244	C	-7.317	1.2	3.1742
H	6.3447	-3.342	5.9288	C	-0.1731	-4.5582	-7.0438	C	-8.3281	0.4222	3.5539
H	12.5789	-2.5345	5.6783	C	1.4772	-2.9958	-8.0041	H	-2.3884	-2.8273	-3.6957
H	10.9497	-3.7088	7.1471	N	2.5957	-2.9082	-8.6166	H	-3.2424	-1.1526	-2.0229
H	8.5164	-3.6063	6.6538	C	3.3807	-1.9843	-9.0308	H	-5.7579	-5.2914	-2.4649
H	9.6114	0.1375	1.3651	C	4.5807	-2.3953	-9.6822	H	-7.2913	-4.5093	-0.6902
H	12.145	0.1495	1.7166	C	5.4679	-1.4128	-10.1489	H	-10.2328	-1.4857	3.9785
H	13.0934	-1.0783	3.6418	C	5.1755	-0.058	-9.9721	H	-10.4862	-3.6372	2.7546
C	7.3962	-4.5882	-0.3704	C	4.0013	0.3348	-9.3312	H	-8.9106	-4.2336	0.9279
C	7.5611	-4.9855	0.8971	C	3.1051	-0.6245	-8.8614	H	-5.6057	1.3845	1.765
C	7.5647	-3.9754	1.969	N	4.8198	-3.6617	-9.8247	H	-7.1309	2.1702	3.6621
N	7.4113	-2.7305	1.7277	C	5.8823	-4.0798	-10.404	H	-9.0088	0.7241	4.3667
C	7.2727	-2.3003	0.5185	C	6.8991	-3.1582	-10.943	Zn	-5.6166	-1.2202	0.0084
C	7.27	-3.1251	-0.5409	C	6.7033	-1.8474	-10.8216	C	3.6313	-13.7108	0.8757
C	7.0786	-0.8277	0.3326	H	-0.1049	-7.9676	-6.9964	C	4.5873	-13.1759	1.6449
N	7.0435	-0.1158	1.3932	H	1.9787	-7.3909	-8.2868	C	5.9853	-13.2186	1.1837
C	6.8894	1.1178	1.7027	H	-0.7294	-3.6704	-6.7132	N	6.315	-13.7475	0.0688
C	6.9135	1.4469	3.0898	H	0.9188	-2.1044	-7.6801	C	5.4296	-14.2963	-0.6936
C	6.7467	2.7877	3.47	H	5.877	0.7088	-10.3401	C	4.1269	-14.3328	-0.3706
C	6.558	3.7768	2.5013	H	3.7811	1.4072	-9.1953	C	5.9045	-14.8731	-1.9907
C	6.5329	3.4455	1.1472	H	2.1822	-0.3002	-8.3567	N	7.1502	-14.7542	-2.2485
C	6.6982	2.12	0.7473	H	6.0437	-5.1644	-10.5047	C	7.9561	-15.0635	-3.1951
N	7.0875	0.5013	3.96	H	7.8009	-3.5556	-11.4359	C	9.3233	-14.6942	-3.0302
C	7.1183	0.7474	5.216	H	7.4357	-1.1206	-11.2094	C	10.2374	-15.0135	-4.0464
C	6.9582	2.1103	5.755	Zn	3.3171	-4.6637	-9.0256	C	9.8077	-15.6785	-5.1977

C	6.7752	3.1175	4.9045	C	-0.0545	-12.3595	4.0422	C	8.4681	-16.0329	-5.3518
H	7.653	-6.0427	1.1824	C	-1.2614	-11.8147	4.238	C	7.5435	-15.7274	-4.3539
H	7.6705	-4.3093	3.013	C	-1.5542	-11.1666	5.5275	N	9.6839	-14.0741	-1.95
H	7.1378	-2.6877	-1.54	N	-0.6902	-11.1036	6.4657	C	10.8998	-13.7232	-1.7559
H	6.9541	-0.3882	-0.6687	C	0.4796	-11.6322	6.3288	C	11.964	-13.9966	-2.7388
H	6.4268	4.8279	2.8066	C	0.855	-12.2631	5.204	C	11.6472	-14.6296	-3.8658
H	6.3817	4.2341	0.3908	C	1.4327	-11.4912	7.4748	H	4.3721	-12.6643	2.5933
H	6.676	1.8728	-0.3252	N	1.0153	-10.8381	8.4912	H	6.7643	-12.7503	1.8053
H	7.2709	-0.086	5.9195	C	1.4647	-10.4713	9.6336	H	3.43	-14.808	-1.0743
H	6.9915	2.283	6.8427	C	0.5891	-9.7095	10.462	H	5.2075	-15.3567	-2.6917
H	6.6515	4.1531	5.2612	C	1.0446	-9.2876	11.7208	H	10.5305	-15.9251	-5.9926
Zn	7.2765	-1.2051	2.9833	C	2.3355	-9.6097	12.1474	H	8.1391	-16.5559	-6.2657
C	-1.1557	-13.7756	-0.675	C	3.1861	-10.3525	11.3297	H	6.489	-16.0127	-4.489
C	-0.7936	-13.8138	-1.9629	C	2.7534	-10.7831	10.076	H	11.1618	-13.2001	-0.8229
C	-1.8372	-13.8479	-2.9999	N	-0.6008	-9.4272	10.0311	H	12.9997	-13.6798	-2.536
N	-3.0799	-13.8361	-2.7086	C	-1.4239	-8.7481	10.7386	H	12.4093	-14.856	-4.6294
C	-3.4774	-13.8151	-1.4802	C	-1.0781	-8.2431	12.0798	C	7.5266	-9.3027	-2.1681
C	-2.6167	-13.7981	-0.448	C	0.1318	-8.5035	12.5692	C	6.999	-10.2435	-2.9608
C	-4.9555	-13.7808	-1.2426	H	-2.0389	-11.7944	3.4617	C	7.17	-11.6614	-2.6028
N	-5.7037	-13.7557	-2.2793	H	-2.5393	-10.6967	5.6739	N	7.8033	-12.0234	-1.5547
C	-6.9563	-13.7101	-2.5461	H	1.8691	-12.683	5.1557	C	8.3449	-11.1523	-0.7707
C	-7.3275	-13.6884	-3.9228	H	2.4472	-11.9157	7.4314	C	8.2686	-9.8313	-1.0033
C	-8.6899	-13.6385	-4.2567	H	2.6873	-9.2748	13.1371	C	9.0485	-11.6712	0.4449
C	-9.6607	-13.6075	-3.2524	H	4.2039	-10.5999	11.6758	N	9.0371	-12.9377	0.618
C	-9.2891	-13.6256	-1.9087	H	3.4357	-11.3687	9.441	C	9.4733	-13.7819	1.4774
C	-7.9412	-13.6772	-1.5547	H	-2.4253	-8.534	10.3337	C	9.1903	-15.1586	1.2371
N	-6.3991	-13.7151	-4.8272	H	-1.8138	-7.6627	12.6595	C	9.6495	-16.1146	2.1567
C	-6.6812	-13.7	-6.076	H	0.4346	-8.1471	13.5674	C	10.3688	-15.7171	3.2866
C	-8.0699	-13.6504	-6.568	C	-0.0071	-6.047	6.0665	C	10.639	-14.3685	3.5149
C	-9.0619	-13.6198	-5.6813	C	1.0676	-6.7641	6.4162	C	10.1932	-13.4022	2.6138
H	0.2552	-13.7819	-2.2894	C	0.8762	-8.0455	7.1169	N	8.5175	-15.4894	0.1792
H	-1.5338	-13.8546	-4.0584	N	-0.2848	-8.4975	7.3979	C	8.2396	-16.712	-0.0805
H	-3.0215	-13.7737	0.5734	C	-1.3482	-7.8296	7.0986	C	8.6606	-17.8183	0.7983
H	-5.3711	-13.7614	-0.2237	C	-1.2978	-6.6416	6.4748	C	9.353	-17.5335	1.8986
H	-10.7291	-13.5678	-3.5213	C	-2.6664	-8.4479	7.4463	H	6.4055	-10.006	-3.8546
H	-10.0639	-13.5993	-1.1239	N	-2.6317	-9.6017	7.9948	H	6.7114	-12.43	-3.2443
H	-7.6627	-13.6913	-0.4899	C	-3.4665	-10.4722	8.4267	H	8.7472	-9.1469	-0.2894
H	-5.8599	-13.7269	-6.8091	C	-2.9273	-11.6736	8.9737	H	9.5344	-10.9897	1.1596
H	-8.2736	-13.6402	-7.6509	C	-3.8094	-12.6524	9.4572	H	10.7261	-16.4729	4.0053
H	-10.1158	-13.5834	-6.0024	C	-5.1903	-12.4482	9.3974	H	11.2068	-14.0655	4.4109
C	-3.8789	-9.1238	-4.5389	C	-5.709	-11.272	8.8576	H	10.4135	-12.3412	2.8069
C	-4.6149	-9.4799	-3.4788	C	-4.8507	-10.2854	8.3735	H	7.6675	-16.9473	-0.9916
C	-4.9096	-10.9057	-3.257	N	-1.6404	-11.829	9.0091	H	8.4031	-18.8584	0.5412
N	-4.4813	-11.8164	-4.0432	C	-1.109	-12.889	9.4922	H	9.6895	-18.3277	2.5851
C	-3.787	-11.5176	-5.0897	C	-1.9162	-13.9963	10.0359	Zn	8.0851	-13.8382	-0.8145
C	-3.4694	-10.2511	-5.4035	C	-3.2426	-13.888	10.0226	C	7.3126	-7.8348	-2.3971
C	-3.3101	-12.652	-5.9422	H	2.0935	-6.4594	6.1667	C	7.5511	-6.9103	-1.3722

N	-3.6123	-13.8302	-5.5502	H	1.762	-8.6459	7.3769	C	7.257	-5.5486	-1.5147
C	-3.4142	-15.0368	-5.9324	H	-2.2447	-6.1345	6.2445	C	6.718	-5.1213	-2.7333
C	-3.9642	-16.0732	-5.1222	H	-3.6154	-7.9378	7.2215	C	6.4561	-6.0059	-3.7854
C	-3.7671	-17.4087	-5.5065	H	-5.8778	-13.2213	9.7784	C	6.7699	-7.3566	-3.5968
C	-3.0416	-17.71	-6.662	H	-6.8009	-11.1219	8.8134	H	6.4426	-4.0669	-2.8477
C	-2.5048	-16.6902	-7.4466	H	-5.274	-9.3619	7.9498	H	6.5332	-8.0681	-4.3965
C	-2.6898	-15.3565	-7.0842	H	-0.0114	-12.9777	9.5042	H	7.9311	-7.2732	-0.4101
N	-4.6286	-15.7549	-4.0551	H	-1.4226	-14.8926	10.4447	C	-1.8563	-6.1594	-5.8428
C	-5.1424	-16.6496	-3.2969	H	-3.8868	-14.6898	10.4192	C	-2.4132	-5.1217	-5.0866
C	-5.0141	-18.0905	-3.5815	Zn	-0.8056	-10.2164	8.2324	C	-3.4563	-5.337	-4.1772
C	-4.3404	-18.4715	-4.6643	C	-2.1945	-2.7664	2.9397	C	-3.951	-6.6406	-4.0479
H	-4.9795	-8.7565	-2.7364	C	-3.2863	-3.5314	2.8193	C	-3.4287	-7.7126	-4.7822
H	-5.4906	-11.1958	-2.3676	C	-4.3841	-3.0822	1.9485	C	-2.3873	-7.4441	-5.6784
H	-2.867	-10.0743	-6.305	N	-4.3254	-1.9853	1.2985	H	-4.7362	-6.8364	-3.3088
H	-2.7134	-12.4749	-6.8498	C	-3.3006	-1.2059	1.3955	H	-1.9372	-8.2774	-6.2294
H	-2.8898	-18.7611	-6.9578	C	-2.2442	-1.5035	2.1715	H	-1.9804	-4.1186	-5.1712
H	-1.9322	-16.9392	-8.356	C	-3.3188	0.0482	0.5771	C	0.0809	-4.7921	5.2485
H	-2.2598	-14.5615	-7.7125	N	-4.3418	0.2338	-0.1675	C	1.3023	-4.1467	5.0218
H	-5.7005	-16.3341	-2.4015	C	-4.7614	1.1079	-1.0054	C	1.4151	-3.0453	4.164
H	-5.4708	-18.8313	-2.9057	C	-6.0226	0.8627	-1.6246	C	0.2526	-2.5857	3.5309
H	-4.2186	-19.5375	-4.9175	C	-6.5168	1.7964	-2.5488	C	-0.9909	-3.1986	3.7247
Zn	-4.6515	-13.7852	-3.9108	C	-5.779	2.9426	-2.8551	C	-1.0511	-4.2934	4.5948
C	5.7597	-5.5452	-5.0316	C	-4.5452	3.173	-2.2479	H	0.3249	-1.7532	2.821
C	5.1172	-4.3734	-5.1063	C	-4.0365	2.2595	-1.3252	H	-2.007	-4.8119	4.7287
C	4.3722	-4.0414	-6.3315	N	-6.6774	-0.2135	-1.3183	H	2.2088	-4.5487	5.4891
N	4.3148	-4.8407	-7.3251	C	-7.8142	-0.4773	-1.8447	C	2.1699	-13.6069	1.2001
C	4.9314	-5.9749	-7.3052	C	-8.4518	0.4165	-2.8286	C	1.7259	-13.1628	2.4512
C	5.6558	-6.3793	-6.2481	C	-7.8212	1.5347	-3.1797	C	0.37	-12.9469	2.728
C	4.7853	-6.8523	-8.5097	H	-3.3901	-4.5072	3.3133	C	-0.5546	-13.1942	1.7048
N	4.0438	-6.4125	-9.4538	H	-5.2687	-3.7278	1.8338	C	-0.1591	-13.6378	0.4375
C	3.6379	-6.8055	-10.6039	H	-1.4011	-0.7994	2.2024	C	1.2065	-13.8476	0.2143
C	2.7704	-5.9299	-11.3211	H	-2.4822	0.7626	0.6063	H	-1.6163	-12.9852	1.8815
C	2.3031	-6.3246	-12.5844	H	-6.1723	3.6717	-3.5825	H	1.5375	-14.1598	-0.7826
C	2.6815	-7.5574	-13.122	H	-3.9704	4.0805	-2.4985	H	2.4671	-12.926	3.2231

Table S14. Cartesian coordinates (in Å) for the PM7 model of $Zn^{II}_9L_6$ (**A** and **B** as subcomponents).

C	-3.6097	-2.2519	15.3505	C	-1.9795	-12.4438	15.6916	C	-12.5215	-0.5982	19.7547
C	-4.5882	-3.1331	15.1349	C	-0.9612	-12.4715	16.6504	C	-12.3276	-0.5139	18.4323
C	-4.44	-4.0881	14.024	C	-0.1401	-11.3638	16.887	C	-11.7084	0.7006	17.8725
N	-3.4099	-4.0897	13.2659	C	-0.3513	-10.1924	16.153	N	-11.3489	1.6811	18.6084
C	-2.4396	-3.2559	13.4511	C	-1.3624	-10.1217	15.1895	C	-11.5295	1.6483	19.8861
C	-2.4552	-2.345	14.4383	C	-2.1434	-11.2604	14.9622	C	-12.0868	0.5975	20.5077
C	-1.2856	-3.328	12.5016	H	0.2636	-9.3055	16.3698	C	-11.0769	2.8363	20.6747
N	-1.36	-4.2053	11.5753	H	-2.909	-11.2111	14.1788	N	-10.534	3.7867	20.0166
C	-0.6484	-4.603	10.5866	H	-0.7847	-13.3809	17.2398	C	-10.0152	4.9317	20.2618
C	-1.1769	-5.6608	9.789	C	-9.5838	-11.5865	28.3126	C	-9.5057	5.6644	19.15
C	-0.4263	-6.1241	8.6972	C	-8.6514	-12.4091	27.8249	C	-8.9253	6.9219	19.3785
C	0.8153	-5.5552	8.4025	C	-9.047	-13.7786	27.4528	C	-8.8507	7.4395	20.6743
C	1.3231	-4.5201	9.1861	N	-10.249	-14.1971	27.58	C	-9.3508	6.7152	21.7556
C	0.5946	-4.0441	10.2756	C	-11.1754	-13.4329	28.0536	C	-9.9326	5.4648	21.5514
N	-2.3332	-6.1638	10.0918	C	-10.9343	-12.1698	28.4348	N	-9.59	5.15	17.9624
C	-2.8492	-7.1133	9.4051	C	-12.5545	-14.0047	28.1549	C	-9.1468	5.7706	16.9339
C	-2.1702	-7.6958	8.2332	N	-12.7054	-15.2077	27.753	C	-8.5144	7.0991	17.0283
C	-0.9803	-7.2158	7.8794	C	-13.6553	-16.061	27.6484	C	-8.4013	7.6711	18.2247
H	-5.4925	-3.1593	15.7624	C	-13.324	-17.3398	27.1107	H	-12.5963	-1.3221	17.7383
H	-5.2496	-4.81	13.8343	C	-14.3382	-18.3013	26.9796	H	-11.539	0.7576	16.7858
H	-1.6164	-1.6408	14.548	C	-15.6467	-18.0035	27.3692	H	-12.1958	0.6441	21.5993
H	-0.4228	-2.6514	12.5969	C	-15.9614	-16.7505	27.8935	H	-11.1995	2.8793	21.7675
H	1.3998	-5.925	7.5441	C	-14.9696	-15.7807	28.033	H	-8.393	8.4275	20.8469
H	2.3037	-4.0768	8.9431	N	-12.0988	-17.5793	26.7591	H	-9.2856	7.1327	22.7747
H	1.0079	-3.2266	10.8858	C	-11.7529	-18.7121	26.2729	H	-10.3231	4.9039	22.4142
H	-3.8338	-7.5098	9.699	C	-12.7152	-19.8124	26.0804	H	-9.24	5.2972	15.944
H	-2.6452	-8.5129	7.667	C	-13.9862	-19.6188	26.4244	H	-8.1447	7.6023	16.1206
H	-0.4345	-7.6201	7.0111	H	-7.6094	-12.089	27.6736	H	-7.9346	8.6626	18.3443
C	-1.6321	-8.8781	14.4076	H	-8.2843	-14.4572	27.0403	C	-6.2661	1.1719	17.8528
C	-0.7049	-7.9534	14.1407	H	-11.7605	-11.5687	28.842	C	-6.644	1.592	19.0632
C	-1.0844	-6.7875	13.3197	H	-13.3937	-13.4112	28.5489	C	-7.9349	2.2873	19.1973
N	-2.2673	-6.6352	12.8552	H	-16.439	-18.7627	27.2626	N	-8.6978	2.497	18.1934
C	-3.1906	-7.5039	13.1034	H	-16.9974	-16.5263	28.199	C	-8.366	2.1057	17.0073
C	-2.9687	-8.5964	13.8485	H	-15.2323	-14.7954	28.4477	C	-7.2135	1.4625	16.7601
C	-4.5422	-7.2656	12.5089	H	-10.7008	-18.8716	25.9896	C	-9.3263	2.3949	15.8967
N	-4.6715	-6.2221	11.7855	H	-12.3797	-20.7739	25.6595	N	-10.4036	3.0038	16.2157
C	-5.5947	-5.6518	11.1052	H	-14.7408	-20.4125	26.2988	C	-11.4575	3.4343	15.6283
C	-5.251	-4.4442	10.4297	C	-11.166	-14.689	22.5282	C	-12.4362	4.0788	16.4407
C	-6.2359	-3.7907	9.6725	C	-12.2208	-14.3438	23.2742	C	-13.6048	4.5649	15.834
C	-7.5273	-4.3178	9.5876	C	-12.2329	-14.716	24.7011	C	-13.7985	4.4177	14.458
C	-7.8537	-5.4995	10.2514	N	-11.2737	-15.3643	25.2449	C	-12.8365	3.7854	13.6716
C	-6.891	-6.1657	11.0085	C	-10.2462	-15.7284	24.5535	C	-11.6686	3.2942	14.2537
N	-4.0423	-3.9847	10.5327	C	-10.1185	-15.4377	23.2514	N	-12.2206	4.1984	17.7139

C	-3.6877	-2.905	9.9427	C	-9.174	-16.4968	25.2593	C	-13.0692	4.7697	18.4838
C	-4.62	-2.1173	9.1153	N	-9.356	-16.7206	26.5029	C	-14.3388	5.3252	17.9807
C	-5.8727	-2.545	8.9777	C	-8.736	-17.301	27.4617	C	-14.6092	5.229	16.681
H	0.3332	-8.0517	14.4925	C	-9.3561	-17.2747	28.7452	H	-6.0302	1.4175	19.96
H	-0.317	-6.0342	13.0825	C	-8.7059	-17.8969	29.8225	H	-8.2522	2.6304	20.1942
H	-3.8021	-9.2896	14.0313	C	-7.4746	-18.5299	29.6329	H	-6.9737	1.1576	15.7305
H	-5.3752	-7.9641	12.6806	C	-6.8754	-18.5499	28.3743	H	-9.1105	2.0928	14.8606
H	-8.2961	-3.7989	8.9917	C	-7.5024	-17.9359	27.2907	H	-14.7181	4.8029	13.9875
H	-8.8756	-5.9081	10.1774	N	-10.4987	-16.6765	28.8824	H	-12.9996	3.6742	12.5863
H	-7.1634	-7.0973	11.5279	C	-11.0932	-16.6267	30.0153	H	-10.9169	2.7984	13.6206
H	-2.6514	-2.5505	10.0559	C	-10.5245	-17.2382	31.2305	H	-12.8442	4.8511	19.5587
H	-4.2785	-1.1927	8.6229	C	-9.3531	-17.8642	31.1442	H	-15.0446	5.8102	18.674
H	-6.6046	-1.9897	8.3686	H	-13.0765	-13.7896	22.8618	H	-15.5453	5.6302	16.2595
Zn	-3.0137	-5.2168	11.6854	H	-13.0955	-14.4221	25.319	Zn	-10.4661	3.3861	18.1183
C	-4.9851	0.4484	17.6234	H	-9.2016	-15.755	22.7361	C	-13.8199	-3.0181	23.9651
C	-3.8546	0.7333	18.3948	H	-8.2742	-16.8445	24.7297	C	-12.9308	-2.4129	24.7568
C	-2.6468	0.0445	18.2247	H	-6.9705	-19.0177	30.4834	C	-13.1096	-2.5037	26.2154
C	-2.5854	-0.929	17.2183	H	-5.9034	-19.0526	28.2357	N	-14.087	-3.14	26.7388
C	-3.6923	-1.2198	16.4161	H	-7.0181	-17.9593	26.3024	C	-14.9708	-3.7318	26.0046
C	-4.8881	-0.5321	16.635	H	-12.0631	-16.1103	30.0865	C	-14.9138	-3.7195	24.6629
H	-1.6664	-1.5123	17.0625	H	-11.0665	-17.1752	32.1879	C	-16.0764	-4.4515	26.7107
H	-5.7667	-0.7702	16.0162	H	-8.8899	-18.3375	32.0256	N	-16.0485	-4.4315	27.9881
H	-3.9279	1.5306	19.1466	Zn	-11.0303	-15.9577	27.1203	C	-16.7454	-4.8946	28.9583
C	2.5598	-7.8184	23.4376	C	-6.4564	-8.0168	30.0225	C	-16.2918	-4.6068	30.2794
C	3.2268	-7.498	22.321	C	-6.0552	-6.7407	30.061	C	-17.0264	-5.1005	31.3689
C	3.8626	-8.563	21.531	C	-4.7226	-6.426	30.6063	C	-18.1794	-5.8604	31.1558
N	3.8025	-9.7881	21.8798	N	-3.9309	-7.3343	31.0324	C	-18.6146	-6.138	29.8607
C	3.1807	-10.1474	22.9525	C	-4.2704	-8.5802	30.9907	C	-17.9007	-5.657	28.7639
C	2.5584	-9.2637	23.752	C	-5.4622	-8.9871	30.5244	N	-15.2151	-3.9014	30.438
C	3.1788	-11.6086	23.2792	C	-3.2771	-9.5792	31.495	C	-14.7688	-3.618	31.6043
N	3.7896	-12.3801	22.4625	N	-2.1752	-9.1143	31.9437	C	-15.4412	-4.0711	32.8357
C	4.0358	-13.6318	22.3434	C	-1.0926	-9.5721	32.453	C	-16.5501	-4.7993	32.7289
C	4.7868	-14.0413	21.2024	C	-0.1056	-8.6202	32.8434	H	-12.0633	-1.87	24.3514
C	5.0775	-15.4046	21.0383	C	1.093	-9.0818	33.4094	H	-12.3723	-2.0159	26.8717
C	4.6364	-16.3379	21.9802	C	1.3091	-10.4513	33.583	H	-15.6931	-4.2339	24.0806
C	3.9023	-15.9281	23.0924	C	0.3379	-11.3751	33.1994	H	-16.8656	-4.9768	26.1517
C	3.6005	-14.5792	23.274	C	-0.861	-10.9382	32.6374	H	-18.7511	-6.246	32.0159
N	5.1805	-13.1462	20.3506	N	-0.3432	-7.3574	32.6693	H	-19.5252	-6.7407	29.7038
C	5.8578	-13.4654	19.3119	C	0.5099	-6.4649	33.0087	H	-18.2551	-5.8849	27.747
C	6.2329	-14.86	19.015	C	1.8099	-6.8082	33.6134	H	-13.8511	-3.0155	31.6924
C	5.8538	-15.818	19.8578	C	2.1044	-8.0908	33.8124	H	-15.027	-3.8076	33.8221
H	3.3299	-6.4682	21.9541	H	-6.685	-5.9135	29.703	H	-17.0865	-5.1607	33.6216
H	4.4052	-8.2913	20.6122	H	-4.4028	-5.3733	30.6546	C	-11.2593	-6.897	28.8755
H	2.0438	-9.6407	24.6462	H	-5.695	-10.0611	30.5426	C	-12.4849	-7.2352	28.4542
H	2.6749	-11.9929	24.179	H	-3.4929	-10.6584	31.4825	C	-13.5283	-6.2017	28.3723
H	4.8692	-17.4074	21.846	H	2.2529	-10.8072	34.0281	N	-13.3028	-4.988	28.6942
H	3.5599	-16.6726	23.831	H	0.518	-12.4539	33.3429	C	-12.1409	-4.614	29.1138

H	3.0207	-14.2696	24.1569	H	-1.6196	-11.6787	32.341	C	-11.1103	-5.4685	29.2287
H	6.1746	-12.672	18.617	H	0.2665	-5.4038	32.8435	C	-11.9781	-3.1689	29.4701
H	6.8179	-15.0962	18.1115	H	2.5189	-6.0119	33.8919	N	-13.0047	-2.4215	29.3204
H	6.1128	-16.8743	19.6782	H	3.064	-8.3954	34.2613	C	-13.3139	-1.189	29.4851
C	0.9195	-11.4314	17.9293	C	-0.0721	-6.5348	27.5857	C	-14.6473	-0.804	29.1568
C	0.7317	-12.0613	19.0915	C	-0.3158	-7.7985	27.9558	C	-15.0246	0.5377	29.3227
C	1.8313	-12.077	20.0703	C	-0.9594	-8.0544	29.2578	C	-14.1047	1.4747	29.8002
N	2.9537	-11.5118	19.8355	N	-1.3042	-7.1081	30.0447	C	-12.8028	1.0901	30.1173
C	3.1747	-10.8978	18.7194	C	-1.1067	-5.8748	29.7163	C	-12.4065	-0.2377	29.9603
C	2.2463	-10.8122	17.7513	C	-0.5229	-5.5203	28.561	N	-15.4727	-1.7009	28.7146
C	4.5227	-10.2726	18.5392	C	-1.5508	-4.8247	30.6849	C	-16.6799	-1.4048	28.4078
N	5.3436	-10.389	19.5121	N	-2.0777	-5.2365	31.7724	C	-17.2101	-0.035	28.5338
C	6.5408	-10.0264	19.7908	C	-2.5726	-4.7233	32.8365	C	-16.4039	0.9245	28.9821
C	7.0509	-10.3976	21.0701	C	-3.0408	-5.6266	33.8349	H	-12.7591	-8.2562	28.1574
C	8.3567	-10.0147	21.4151	C	-3.5862	-5.1034	35.0178	H	-14.5314	-6.4862	28.0182
C	9.1372	-9.2821	20.5172	C	-3.6671	-3.7211	35.2056	H	-10.1484	-5.0781	29.5872
C	8.6308	-8.9232	19.2687	C	-3.2061	-2.8451	34.2239	H	-11.0185	-2.775	29.8376
C	7.3364	-9.2934	18.9052	C	-2.6579	-3.3433	33.0427	H	-14.4077	2.527	29.9274
N	6.2961	-11.0744	21.8789	N	-2.9463	-6.9029	33.6253	H	-12.0848	1.8383	30.4933
C	6.7095	-11.43	23.0374	C	-3.3486	-7.7529	34.4944	H	-11.3755	-0.5273	30.2148
C	8.0594	-11.0968	23.5283	C	-3.9427	-7.343	35.78	H	-17.3465	-2.1996	28.0385
C	8.8742	-10.4022	22.7377	C	-4.0639	-6.0442	36.0443	H	-18.2554	0.1814	28.2605
H	-0.2237	-12.5484	19.3408	H	-0.0503	-8.6605	27.3277	H	-16.7563	1.9628	29.0938
H	1.6762	-12.5874	21.0335	H	-1.1414	-9.0959	29.5658	Zn	-14.5218	-3.4304	28.6491
H	2.4794	-10.2836	16.8144	H	-0.3724	-4.4501	28.3655	C	-13.7139	-2.9924	22.4807
H	4.7848	-9.7348	17.6157	H	-1.4221	-3.7535	30.4678	C	-13.2182	-1.863	21.8242
H	10.1616	-8.9837	20.7958	H	-4.0967	-3.3167	36.1369	C	-13.0918	-1.8123	20.431
H	9.2564	-8.3446	18.5679	H	-3.2733	-1.7555	34.383	C	-13.5019	-2.9381	19.7031
H	6.9499	-9.002	17.9168	H	-2.2955	-2.6403	32.2772	C	-13.9878	-4.0954	20.3259
H	6.0344	-12.0044	23.691	H	-3.2487	-8.8275	34.2756	C	-14.0838	-4.1043	21.7213
H	8.3792	-11.4207	24.5319	H	-4.278	-8.1043	36.5026	H	-13.4464	-2.9202	18.6079
H	9.892	-10.1302	23.0614	H	-4.5018	-5.6909	36.9922	H	-14.4243	-5.0109	22.2427
Zn	4.561	-11.3821	20.9863	Zn	-2.13	-7.1757	31.8468	H	-12.9306	-0.9987	22.4367
C	1.3083	-3.032	24.2235	C	-7.8058	-8.4365	29.5235	C	-9.8484	-14.6722	18.9576
C	0.3885	-2.1236	24.5607	C	-8.8667	-7.5217	29.4458	C	-10.6157	-13.652	18.3907
C	0.5204	-0.7557	24.0293	C	-10.1351	-7.8875	28.9755	C	-11.5903	-12.9799	19.1358
N	1.4808	-0.4168	23.2559	C	-10.3227	-9.2208	28.597	C	-11.7758	-13.3234	20.4793
C	2.3913	-1.265	22.9122	C	-9.3074	-10.1734	28.6979	C	-11.0177	-14.3354	21.0823
C	2.3842	-2.5352	23.3428	C	-8.0501	-9.7633	29.1481	C	-10.0841	-15.0139	20.2916
C	3.4673	-0.7838	21.9904	H	-11.2899	-9.5452	28.1943	H	-12.5104	-12.7557	21.0696
N	3.3901	0.4324	21.6073	H	-7.2444	-10.5073	29.2131	H	-9.5189	-15.8504	20.723
C	4.046	1.232	20.8513	H	-8.6971	-6.4894	29.7747	H	-10.4537	-13.3686	17.3397
C	3.5455	2.5612	20.7221	C	-14.3853	-5.2888	19.5162	C	-8.7971	-15.3828	18.1745
C	4.2352	3.4658	19.8996	C	-15.2332	-6.221	19.9636	C	-7.7098	-15.9011	18.7538
C	5.3902	3.0645	19.2231	C	-15.5794	-7.351	19.0836	C	-6.7109	-16.5775	17.9109
C	5.8718	1.7628	19.3552	N	-15.0994	-7.4649	17.9045	N	-6.8524	-16.6764	16.6455
C	5.2026	0.8477	20.1669	C	-14.2769	-6.5862	17.4354	C	-7.8878	-16.1866	16.0464

N	2.4699	2.8969	21.3646	C	-13.8777	-5.5209	18.1488	C	-8.8694	-15.5532	16.7105
C	1.983	4.0779	21.2795	C	-13.785	-6.7855	16.0366	C	-7.9538	-16.3429	14.5596
C	2.605	5.1278	20.4521	N	-14.2316	-7.8073	15.4135	N	-6.9721	-16.943	14.0026
C	3.7107	4.8353	19.7713	C	-14.1009	-8.3423	14.2569	C	-6.6169	-17.2783	12.8186
H	-0.4716	-2.3694	25.2013	C	-14.8375	-9.5378	14.0075	C	-5.3743	-17.9646	12.6837
H	-0.2421	-0.0074	24.2959	C	-14.7214	-10.1538	12.7516	C	-4.9488	-18.3462	11.4019
H	3.1952	-3.2071	23.0264	C	-13.8989	-9.5991	11.7675	C	-5.7325	-18.055	10.2823
H	4.2747	-1.4519	21.6543	C	-13.1832	-8.4295	12.0198	C	-6.9469	-17.3852	10.4238
H	5.9272	3.7799	18.5786	C	-13.2826	-7.8016	13.2608	C	-7.3893	-16.9967	11.6877
H	6.7839	1.4561	18.8158	N	-15.593	-10.0216	14.9442	N	-4.673	-18.2154	13.7456
H	5.5939	-0.1768	20.2622	C	-16.2746	-11.0909	14.7668	C	-3.5514	-18.8292	13.6746
H	1.0681	4.319	21.843	C	-16.2464	-11.8402	13.4975	C	-2.9853	-19.2874	12.3926
H	2.1561	6.1328	20.4031	C	-15.4861	-11.3872	12.5035	C	-3.6648	-19.0538	11.2724
H	4.2097	5.5877	19.1388	H	-15.6984	-6.163	20.9583	H	-7.5275	-15.8229	19.8358
C	-1.4764	0.3302	19.1148	H	-16.2881	-8.1107	19.4492	H	-5.8121	-17.0004	18.3861
C	-0.2383	-0.1129	18.8676	H	-13.1664	-4.8159	17.6951	H	-9.7341	-15.162	16.1537
C	0.8298	0.1753	19.841	H	-13.084	-6.077	15.5698	H	-8.8042	-15.9518	13.9804
N	0.6133	0.8432	20.9088	H	-13.8148	-10.086	10.782	H	-5.3915	-18.3565	9.2783
C	-0.5609	1.3095	21.1733	H	-12.5385	-7.9988	11.2349	H	-7.5594	-17.1622	9.5335
C	-1.6124	1.1047	20.3658	H	-12.7129	-6.8781	13.446	H	-8.3492	-16.4666	11.784
C	-0.7217	2.0935	22.4379	H	-16.9042	-11.4642	15.5895	H	-2.9894	-19.0217	14.6019
N	0.319	2.2237	23.1669	H	-16.8473	-12.7564	13.3819	H	-2.0166	-19.8126	12.3712
C	0.6348	2.7748	24.2793	H	-15.4335	-11.9137	11.5364	H	-3.2808	-19.3774	10.2911
C	1.9827	2.6243	24.7186	C	-12.4064	-11.9084	18.5038	C	-2.8818	-13.6232	15.4722
C	2.3672	3.2101	25.9348	C	-11.8826	-11.007	17.6699	C	-3.921	-13.5957	14.6283
C	1.4406	3.928	26.6953	C	-12.7804	-10.018	17.0486	C	-4.7971	-14.7742	14.5278
C	0.1242	4.0689	26.2585	N	-14.0384	-10.0038	17.2806	N	-4.5958	-15.8273	15.2201
C	-0.2797	3.493	25.0547	C	-14.5778	-10.856	18.0899	C	-3.5957	-15.907	16.0332
N	2.8135	1.95	23.9862	C	-13.86	-11.7974	18.7261	C	-2.7278	-14.8969	16.2067
C	4.0334	1.7863	24.3392	C	-16.0593	-10.7723	18.2831	C	-3.441	-17.1798	16.8049
C	4.5721	2.347	25.5919	N	-16.6738	-9.8708	17.6175	N	-4.3251	-18.0806	16.6064
C	3.7607	3.0476	26.3808	C	-17.8782	-9.4599	17.4676	C	-4.587	-19.2596	17.0329
H	0.0144	-0.6941	17.9693	C	-18.0875	-8.3753	16.5654	C	-5.7474	-19.8975	16.5046
H	1.8444	-0.2056	19.6451	C	-19.3937	-7.8985	16.3725	C	-6.0717	-21.188	16.951
H	-2.5874	1.5079	20.671	C	-20.4649	-8.4794	17.056	C	-5.268	-21.8308	17.896
H	-1.6918	2.5271	22.7241	C	-20.2511	-9.5397	17.9355	C	-4.1344	-21.1999	18.4066
H	1.7498	4.3879	27.6484	C	-18.9622	-10.0304	18.1412	C	-3.7942	-19.9173	17.978
H	-0.5991	4.6377	26.8669	N	-17.0692	-7.8612	15.948	N	-6.4711	-19.2686	15.6313
H	-1.322	3.6124	24.722	C	-17.2077	-6.8881	15.1276	C	-7.5221	-19.8001	15.1289
H	4.7055	1.2082	23.6859	C	-18.5221	-6.2897	14.831	C	-7.9786	-21.1499	15.5071
H	5.6281	2.1854	25.862	C	-19.6002	-6.7799	15.438	C	-7.2727	-21.8378	16.4015
H	4.1193	3.4867	27.3263	H	-10.8064	-10.9909	17.4379	H	-4.1705	-12.7203	14.0139
Zn	1.848	1.3219	22.3815	H	-12.3547	-9.2816	16.3496	H	-5.6616	-14.7378	13.8464
C	1.264	-4.4521	24.6794	H	-14.3645	-12.5146	19.3916	H	-1.9095	-15.0343	16.9264
C	0.6324	-4.8288	25.8668	H	-16.5875	-11.4634	18.9577	H	-2.6124	-17.3213	17.5153
C	0.6049	-6.1623	26.2972	H	-21.4879	-8.0993	16.9006	H	-5.5292	-22.8445	18.2421
C	1.2524	-7.1215	25.502	H	-21.1042	-9.9919	18.4687	H	-3.5062	-21.7165	19.1519

C	1.8792	-6.7885	24.2929	H	-18.8088	-10.8689	18.8378	H	-2.8968	-19.4311	18.3905
C	1.8532	-5.4467	23.8988	H	-16.3155	-6.4833	14.6248	H	-8.1068	-19.2306	14.3898
H	1.2772	-8.1639	25.8414	H	-18.6025	-5.4531	14.1187	H	-8.8895	-21.5736	15.0538
H	2.2976	-5.1493	22.9415	H	-20.6037	-6.3655	15.2473	H	-7.5763	-22.8499	16.7154
H	0.158	-4.0471	26.4731	Zn	-15.4502	-8.8385	16.5188	Zn	-5.648	-17.5021	15.3081

Table S15. Cartesian coordinates (in Å) for the PM7 model of $Zn^{II}{}_9L_6$ **2** (**A** and **C** as subcomponents).

C	-12.1919	0.0624	20.351	C	0.0412	-15.6037	23.5304	C	-16.414	-4.1242	12.7322
C	-11.2434	-0.3994	19.5222	C	1.1771	-15.8679	22.7641	C	-17.1125	-5.2324	12.2263
C	-10.0613	0.4368	19.2479	C	1.1146	-16.812	21.7377	C	-16.8222	-5.7221	10.9523
N	-9.937	1.6934	19.7932	C	-0.0798	-17.4812	21.4701	C	-15.8604	-5.0826	10.1686
C	-10.8939	2.1243	20.6901	C	-1.2233	-17.1996	22.2165	C	-15.1852	-3.9646	10.6587
C	-11.9791	1.3907	20.9713	N	0.0472	-14.6513	24.5465	C	-15.4575	-3.4871	11.9402
C	-10.652	3.4617	21.271	C	1.24	-14.0569	24.9325	N	-18.0416	-5.8533	13.0563
N	-9.5578	4.1576	20.8218	C	2.4527	-14.3042	24.1195	C	-18.8357	-6.8783	12.5622
C	-8.7695	5.1689	21.3431	C	2.4195	-15.1391	23.078	C	-18.5311	-7.4294	11.222
C	-7.5964	5.4446	20.6215	H	-6.6352	-12.2893	26.2894	C	-17.5608	-6.9033	10.4705
C	-6.6788	6.3764	21.1083	H	-4.086	-12.0711	26.0665	H	-16.7117	-4.4481	20.0862
C	-6.9512	7.0603	22.2943	H	-6.2921	-16.0928	24.2001	H	-17.7684	-5.7455	18.1479
C	-8.1297	6.8076	22.9969	H	-3.9888	-16.9814	23.4621	H	-15.125	-1.6946	17.1047
C	-9.0366	5.859	22.526	H	2.0103	-17.0312	21.1339	H	-15.4952	-2.1186	14.6129
N	-7.3547	4.7154	19.4597	H	-0.1231	-18.2242	20.6562	H	-15.6317	-5.4593	9.1584
C	-6.2604	5.0212	18.6636	H	-2.1682	-17.7153	21.9797	H	-14.4256	-3.4643	10.035
C	-5.2629	5.9914	19.1699	C	1.4163	-13.2602	26.2161	H	-14.9031	-2.6152	12.3235
C	-5.445	6.6137	20.3372	H	3.4064	-13.8255	24.3988	C	-20.087	-7.3947	13.2561
H	-11.3219	-1.3959	19.0581	H	3.3334	-15.3251	22.4904	H	-19.1381	-8.2579	10.8196
H	-9.2611	0.0587	18.5943	C	-1.7248	-11.1107	21.1977	H	-17.3552	-7.3133	9.4681
H	-12.7378	1.7991	21.6577	C	-2.0114	-12.3851	20.8915	Zn	-17.6876	-5.2594	14.9381
H	-11.3454	3.9402	21.9808	C	-2.1179	-13.3768	21.9777	C	-12.8352	-12.8589	17.563
H	-6.2336	7.8022	22.6809	N	-1.8926	-13.0261	23.2893	C	-13.689	-12.7606	18.5935
H	-8.3372	7.349	23.9351	C	-1.6398	-11.7023	23.5846	C	-14.0673	-13.9772	19.3334
H	-9.9525	5.6483	23.1022	C	-1.5526	-10.7698	22.6288	N	-13.5898	-15.2134	18.9654
C	-6.0771	4.5146	17.2413	C	-1.4426	-11.3988	25.0171	C	-12.6568	-15.2832	17.9501
H	-4.3766	6.2343	18.5598	N	-1.4639	-12.4514	25.8961	C	-12.2888	-14.2005	17.2509
H	-4.701	7.3441	20.6953	C	-1.7501	-12.5617	27.2461	C	-12.129	-16.6365	17.6783
C	-5.4242	0.7895	22.3118	C	-1.9348	-13.8732	27.7121	N	-12.6383	-17.6754	18.4166
C	-6.2654	1.6235	22.9428	C	-2.3139	-14.0984	29.0361	C	-12.173	-18.9328	18.7608
C	-7.2754	2.356	22.1573	C	-2.4734	-13.0163	29.9034	C	-12.9149	-19.5973	19.7513
N	-7.3499	2.2234	20.7901	C	-2.2652	-11.7142	29.4482	C	-12.4873	-20.8367	20.2298
C	-6.4923	1.3419	20.1647	C	-1.908	-11.485	28.12	C	-11.3408	-21.4273	19.6956
C	-5.5736	0.6458	20.845	N	-1.7925	-14.9171	26.8015	C	-10.6183	-20.7797	18.6936
C	-6.6484	1.2403	18.699	C	-1.852	-16.2315	27.2415	C	-11.0301	-19.5315	18.2287
N	-7.575	2.0567	18.1014	C	-2.2748	-16.4955	28.6359	N	-14.0401	-18.9589	20.2687
C	-8.3125	1.9528	16.9343	C	-2.5216	-15.4893	29.4781	C	-14.8544	-19.6188	21.1785
C	-9.3926	2.8435	16.8278	H	-2.1603	-12.7068	19.8483	C	-14.4087	-20.925	21.7154
C	-10.2564	2.7694	15.7344	H	-2.3664	-14.4226	21.7433	C	-13.2784	-21.4914	21.2871
C	-10.02	1.8303	14.7291	H	-1.3477	-9.7284	22.9226	H	-14.1033	-11.7893	18.9082
C	-8.9318	0.9623	14.8204	H	-1.2144	-10.3865	25.3871	H	-14.7517	-13.9033	20.1912
C	-8.0804	1.0192	15.9232	H	-2.765	-13.1878	30.9523	H	-11.5794	-14.3204	16.4171
N	-9.604	3.7422	17.8697	H	-2.3961	-10.8632	30.1374	H	-11.3949	-16.843	16.8834

C	-10.5826	4.7188	17.7534	H	-1.7683	-10.4507	27.7654	H	-11.0025	-22.4093	20.0644
C	-11.5261	4.6458	16.6144	C	-1.3912	-17.4245	26.4184	H	-9.7128	-21.2512	18.2763
C	-11.3922	3.7071	15.6743	H	-2.3485	-17.5348	28.9981	H	-10.4403	-19.0195	17.4509
H	-6.2141	1.7796	24.0321	H	-2.8205	-15.7005	30.5179	C	-16.2414	-19.1424	21.5812
H	-7.9778	3.0342	22.664	Zn	-1.8524	-14.1512	24.9488	H	-15.0344	-21.457	22.4518
H	-4.9211	-0.0486	20.2931	C	-10.8598	-13.9601	28.0277	H	-12.9665	-22.471	21.685
H	-6.0172	0.5871	18.0757	C	-12.1325	-13.5346	28.0386	C	-11.0319	-15.6735	23.3083
H	-10.6933	1.7721	13.8586	C	-12.78	-13.2291	29.3261	C	-10.4502	-16.3469	22.3016
H	-8.7515	0.2209	14.0241	N	-12.1124	-13.397	30.5161	C	-11.278	-16.853	21.1922
H	-7.2366	0.3137	15.9948	C	-10.7865	-13.7803	30.4796	N	-12.6394	-16.6658	21.1741
C	-10.676	5.9409	18.6542	C	-10.1634	-14.0738	29.3302	C	-13.2219	-15.9841	22.2215
H	-12.3249	5.4002	16.5159	C	-10.1166	-13.8424	31.7951	C	-12.502	-15.4927	23.2382
H	-12.0917	3.6749	14.8229	N	-10.8636	-13.5345	32.9047	C	-14.6877	-15.8243	22.1268
Zn	-8.5601	3.1133	19.4622	C	-10.5324	-13.0326	34.1523	N	-15.3201	-16.3574	21.0306
C	-15.2275	-2.1751	19.8778	C	-11.6075	-12.5289	34.9027	C	-16.4777	-15.97	20.374
C	-15.8034	-2.0895	21.1508	C	-11.3764	-11.9312	36.1425	C	-16.5944	-16.4068	19.0444
C	-15.2021	-1.3631	22.1868	C	-10.0771	-11.8677	36.6486	C	-17.6815	-16.0035	18.2673
C	-14.0068	-0.6892	21.9071	C	-9.0118	-12.3914	35.9165	C	-18.6728	-15.1975	18.8289
C	-13.4291	-0.7155	20.6355	C	-9.237	-12.9705	34.6683	C	-18.5758	-14.7899	20.159
C	-14.0416	-1.4756	19.6362	N	-12.8827	-12.5954	34.3475	C	-17.4795	-15.1727	20.9307
H	-13.4886	-0.1333	22.7013	C	-13.9861	-12.2101	35.0959	N	-15.5716	-17.1926	18.5212
H	-13.5827	-1.5126	18.6372	C	-13.7629	-11.5522	36.4035	C	-15.6989	-17.7401	17.2519
H	-16.7664	-2.5886	21.3269	C	-12.5291	-11.3948	36.8884	C	-16.8297	-17.3052	16.4006
C	-9.0651	-4.2218	30.3017	H	-12.6988	-13.3877	27.105	C	-17.7528	-16.462	16.8683
C	-8.9834	-2.8817	30.2413	H	-13.817	-12.8629	29.3435	H	-9.3672	-16.543	22.2815
C	-7.67	-2.2249	30.3607	H	-9.114	-14.4063	29.3622	H	-10.8074	-17.4079	20.3671
N	-6.5225	-2.9494	30.5761	H	-9.0637	-14.1438	31.9139	H	-13.0289	-14.9341	24.0262
C	-6.6213	-4.3251	30.6247	H	-9.8892	-11.4044	37.6309	H	-15.2788	-15.3057	22.8981
C	-7.792	-4.9622	30.4845	H	-7.9875	-12.3385	36.3219	H	-19.5383	-14.8835	18.223
C	-5.3544	-5.0327	30.9043	H	-8.3847	-13.3656	34.092	H	-19.3633	-14.1551	20.5983
N	-4.2545	-4.2607	31.1795	C	-15.4248	-12.5266	34.7172	H	-17.4081	-14.83	21.9757
C	-2.8895	-4.4752	31.2397	H	-14.6265	-11.2219	37.0051	C	-14.8215	-18.8598	16.7131
C	-2.1114	-3.3304	31.477	H	-12.3788	-10.9174	37.8706	H	-16.9368	-17.7132	15.3813
C	-0.7195	-3.4306	31.511	C	-11.8468	-8.3667	31.1391	H	-18.5988	-16.1599	16.2294
C	-0.1084	-4.6723	31.3271	C	-10.795	-9.0534	31.6224	Zn	-13.9788	-17.0283	19.7281
C	-0.8824	-5.8104	31.1014	C	-10.9724	-10.4419	32.0856	C	-12.4581	-11.6713	16.7444
C	-2.2721	-5.7128	31.0543	N	-12.2006	-11.0565	32.0594	C	-13.3811	-10.6484	16.5087
N	-2.7655	-2.1099	31.6407	C	-13.2596	-10.3584	31.5201	C	-13.0742	-9.5456	15.7034
C	-2.0406	-0.9808	32.0003	C	-13.1404	-9.0944	31.0929	C	-11.8028	-9.5063	15.1143
C	-0.5618	-1.0602	32.0189	C	-14.5265	-11.1135	31.4296	C	-10.8346	-10.4903	15.357
C	0.0661	-2.2096	31.7623	N	-14.5303	-12.4129	31.8719	C	-11.1828	-11.5637	16.1854
H	-9.8727	-2.2468	30.1116	C	-15.278	-13.5113	31.4791	H	-11.5611	-8.6897	14.4225
H	-7.5994	-1.1298	30.2852	C	-14.7378	-14.7617	31.8208	H	-10.443	-12.3424	16.4182
H	-7.7921	-6.0611	30.5328	C	-15.3648	-15.9345	31.3972	H	-14.3775	-10.7359	16.9607
H	-5.2875	-6.1281	30.9996	C	-16.5532	-15.86	30.669	C	-0.5361	-7.9527	19.4504
H	0.9899	-4.7578	31.3629	C	-17.1092	-14.6194	30.3578	C	-1.3099	-7.9988	18.2843
H	-0.3936	-6.7883	30.9557	C	-16.4719	-13.4459	30.7584	C	-2.1855	-9.0594	18.0263

H	-2.875	-6.6155	30.8628	N	-13.54	-14.7853	32.5298	C	-2.3202	-10.078	18.9724
C	-2.6624	0.3227	32.4764	C	-13.023	-15.9934	32.9761	C	-1.5818	-10.054	20.1577
H	0.0317	-0.1686	32.2826	C	-13.6574	-17.2508	32.5187	C	-0.6773	-9.0073	20.3601
H	1.167	-2.2565	31.7957	C	-14.7487	-17.2261	31.7502	H	-3.0429	-10.8882	18.7888
C	-3.7689	-1.5266	26.4427	H	-9.786	-8.6212	31.6822	H	-0.0518	-9.0369	21.2621
C	-3.4659	-2.757	26.8811	H	-10.1099	-11.0026	32.4755	H	-1.2294	-7.1999	17.5338
C	-3.6938	-3.087	28.3001	H	-14.0397	-8.6131	30.6846	H	-7.0496	4.428	16.7114
N	-4.1908	-2.1514	29.179	H	-15.4515	-10.6748	31.023	H	-5.4506	5.2107	16.6412
C	-4.499	-0.8931	28.7024	H	-17.0576	-16.7827	30.339	H	-5.572	3.5243	17.229
C	-4.3185	-0.5584	27.4179	H	-18.0494	-14.5667	29.7836	H	-11.1643	6.7924	18.1312
C	-5.0289	0.0479	29.7107	H	-16.9124	-12.4721	30.4895	H	-9.6701	6.296	18.9639
N	-5.1387	-0.3961	31.0055	C	-11.9161	-16.1146	34.0123	H	-11.2771	5.726	19.5643
C	-5.9972	-0.0376	32.0319	H	-13.2498	-18.2215	32.8481	H	-3.5201	0.1377	33.1576
C	-6.1162	-0.9742	33.0717	H	-15.2201	-18.1698	31.43	H	-1.933	0.9371	33.0488
C	-7.0394	-0.7649	34.0974	Zn	-12.6957	-12.9723	32.3872	H	-3.007	0.9409	31.6193
C	-7.8049	0.4022	34.1133	C	-10.1635	-14.2823	26.7503	H	-3.3465	-3.8501	33.8815
C	-7.6571	1.3529	33.1037	C	-10.8669	-14.8295	25.6723	H	-4.25	-4.4429	35.2973
C	-6.7586	1.1323	32.0609	C	-10.2434	-15.122	24.4534	H	-4.6589	-5.0765	33.6746
N	-5.3497	-2.1349	32.9981	C	-8.8674	-14.8869	24.3471	H	-21.257	-2.0106	27.9276
C	-5.3727	-3.0445	34.0463	C	-8.1296	-14.3767	25.4186	H	-22.4068	-1.0951	26.9211
C	-6.3616	-2.856	35.1323	C	-8.7911	-14.0651	26.6088	H	-21.0961	-2.0176	26.1258
C	-7.1686	-1.7928	35.1455	H	-8.3442	-15.0828	23.3996	H	-19.075	0.2044	30.505
H	-3.069	-3.5248	26.1967	H	-8.2152	-13.6501	27.45	H	-19.1485	-0.2803	32.2168
H	-3.4642	-4.0969	28.6716	H	-11.9347	-15.0548	25.8026	H	-17.5672	-0.2773	31.3802
H	-4.5675	0.4621	27.0853	C	-9.477	-10.4046	14.7281	H	2.4694	-13.2947	26.5723
H	-5.3008	1.0872	29.4672	C	-8.6891	-11.4855	14.5977	H	0.7913	-13.6694	27.0383
H	-8.5301	0.5762	34.9249	C	-7.3654	-11.3517	13.9646	H	1.1556	-12.1902	26.065
H	-8.2646	2.2732	33.1216	N	-6.924	-10.1449	13.4783	H	-0.5174	-17.1669	25.7827
H	-6.6704	1.8792	31.2553	C	-7.7493	-9.0471	13.6062	H	-1.0702	-18.2662	27.0707
C	-4.3554	-4.1607	34.2265	C	-8.9473	-9.1217	14.2029	H	-2.2087	-17.8054	25.7685
H	-6.4069	-3.5792	35.9639	C	-7.2234	-7.7995	13.0137	H	-16.0833	-12.5533	35.6132
H	-7.89	-1.6571	35.9679	N	-6.0222	-7.8678	12.3522	H	-15.5084	-13.5237	34.2347
Zn	-4.6908	-2.3279	31.113	C	-5.0576	-6.9235	12.0438	H	-15.8383	-11.7574	34.0291
C	-13.9649	-4.268	29.1428	C	-3.8114	-7.4402	11.6529	H	-11.9831	-15.31	34.7752
C	-14.8904	-4.922	28.4257	C	-2.7495	-6.5743	11.3864	H	-11.9814	-17.0787	34.5632
C	-16.0972	-4.2024	27.9837	C	-2.9407	-5.1945	11.4769	H	-10.9124	-16.0764	33.5357
N	-16.3051	-2.8849	28.3198	C	-4.1852	-4.6801	11.8395	H	-1.4921	-10.9626	9.9732
C	-15.3159	-2.2168	29.0145	C	-5.2423	-5.5424	12.1261	H	-3.2384	-11.2208	10.2064
C	-14.2008	-2.8342	29.43	N	-3.6635	-8.8242	11.591	H	-2.0895	-11.4666	11.582
C	-15.5992	-0.7925	29.2882	C	-2.4861	-9.3725	11.0998	H	-6.9146	-8.2206	8.3982
N	-16.8108	-0.296	28.8757	C	-1.3387	-8.4741	10.836	H	-5.6018	-7.9926	9.5799
C	-17.2761	0.9745	28.5823	C	-1.4519	-7.1539	10.9965	H	-7.3272	-7.8011	10.0879
C	-18.5145	1.0237	27.9211	H	-9.0032	-12.4826	14.9417	H	-19.0442	-3.1942	12.5975
C	-19.038	2.2507	27.5106	H	-6.717	-12.2345	13.8656	H	-20.067	-1.7651	12.8907
C	-18.3434	3.4288	27.7903	H	-9.5446	-8.2016	14.286	H	-18.4207	-1.8189	13.5916
C	-17.1264	3.3826	28.4699	H	-7.7833	-6.851	13.0281	H	-20.8115	-7.814	12.5236
C	-16.59	2.1571	28.8627	H	-2.1083	-4.5061	11.2578	H	-20.6182	-6.5821	13.796

N	-19.1621	-0.1793	27.6473	H	-4.3309	-3.589	11.907	H	-19.8425	-8.205	13.9769
C	-20.4307	-0.1708	27.0841	H	-6.2168	-5.1244	12.4266	H	-16.8688	-19.9839	21.9491
C	-20.995	1.1175	26.6208	C	-2.3232	-10.831	10.7006	H	-16.7837	-18.6973	20.7199
C	-20.3284	2.2603	26.7987	H	-0.3886	-8.8934	10.4642	H	-16.1868	-18.3921	22.3998
H	-14.76	-5.981	28.1494	H	-0.5944	-6.496	10.7799	H	-14.5437	-19.5791	17.5125
H	-16.8526	-4.7259	27.3798	C	-2.9657	-9.0998	16.7625	H	-15.3499	-19.4472	15.9302
H	-13.4592	-2.2683	30.0159	C	-3.5978	-8.0131	16.2977	H	-13.8937	-18.4584	16.2509
H	-14.9079	-0.144	29.8495	C	-4.3368	-8.105	15.0261	C	-0.9367	-1.436	22.2705
H	-18.756	4.4012	27.4755	N	-4.3633	-9.2736	14.2999	C	-0.9067	-1.4557	20.927
H	-16.5823	4.3169	28.6878	C	-3.7143	-10.3831	14.8057	C	0.3404	-1.8106	20.2291
H	-15.6186	2.1313	29.3828	C	-3.0485	-10.346	15.9684	N	1.4883	-2.103	20.924
C	-21.3378	-1.3891	27.0104	C	-3.7825	-11.5896	13.9552	C	1.4137	-2.1736	22.3008
H	-21.9948	1.1422	26.1553	N	-4.4211	-11.4786	12.745	C	0.307	-1.8295	22.9757
H	-20.7675	3.2137	26.4618	C	-5.0369	-12.3962	11.9108	C	2.6301	-2.6863	22.9649
C	-15.8222	-1.3025	23.5486	C	-5.8618	-11.846	10.916	N	3.6809	-3.0707	22.1685
C	-15.5045	-0.3354	24.4262	C	-6.5987	-12.6847	10.0782	C	4.6531	-4.0446	22.335
C	-16.1277	-0.3283	25.7609	C	-6.4826	-14.0697	10.2079	C	5.3104	-4.4484	21.161
N	-17.0407	-1.287	26.1263	C	-5.64	-14.6167	11.1753	C	6.246	-5.4832	21.201
C	-17.3809	-2.2546	25.2049	C	-4.9202	-13.7822	12.0291	C	6.5564	-6.088	22.4199
C	-16.8213	-2.3073	23.9883	N	-5.9626	-10.4584	10.8395	C	5.9282	-5.6675	23.592
C	-18.3888	-3.2294	25.6717	C	-6.6921	-9.8733	9.8137	C	4.9754	-4.6502	23.5508
N	-18.8799	-3.0826	26.9466	C	-7.4991	-10.7431	8.9277	N	4.9514	-3.8323	19.9659
C	-19.3747	-4.0204	27.8397	C	-7.4764	-12.0705	9.0662	C	5.6423	-4.1248	18.7986
C	-19.3297	-3.6521	29.1946	H	-3.5863	-7.0635	16.8576	C	6.6219	-5.2351	18.8123
C	-19.7258	-4.5581	30.1799	H	-4.8789	-7.2272	14.6449	C	6.8896	-5.8964	19.9408
C	-20.2056	-5.8159	29.8119	H	-2.5276	-11.2492	16.325	H	-1.797	-1.2313	20.32
C	-20.2844	-6.1705	28.4656	H	-3.2921	-12.5379	14.2266	H	0.364	-1.8317	19.1295
C	-19.8681	-5.2762	27.4804	H	-7.0571	-14.7366	9.5445	H	0.3282	-1.8695	24.0753
N	-18.8274	-2.3943	29.5162	H	-5.5528	-15.7118	11.2731	H	2.7095	-2.7932	24.0583
C	-18.8644	-1.9481	30.8303	H	-4.2732	-14.2262	12.8031	H	7.3009	-6.8998	22.46
C	-19.2555	-2.9012	31.8939	C	-6.6297	-8.3958	9.459	H	6.1778	-6.1491	24.5522
C	-19.6368	-4.1441	31.5916	H	-8.0989	-10.2946	8.1179	H	4.4734	-4.341	24.4819
H	-14.795	0.4686	24.1789	H	-8.0713	-12.7065	8.3903	C	5.5525	-3.2949	17.5269
H	-15.8745	0.4663	26.478	Zn	-5.2198	-9.6743	12.5303	H	7.1678	-5.4954	17.8898
H	-17.1155	-3.1262	23.3151	C	-14.0871	-8.464	15.46	H	7.6324	-6.711	19.9401
H	-18.7553	-4.0522	25.0376	C	-13.744	-7.2673	14.9502	C	0.4181	-6.8271	19.7206
H	-20.5279	-6.5321	30.5853	C	-14.7778	-6.2377	14.7375	C	0.944	-6.6285	20.9424
H	-20.6678	-7.1644	28.1805	N	-16.0995	-6.4822	15.0221	C	1.8763	-5.5112	21.1674
H	-19.9218	-5.5759	26.4212	C	-16.4205	-7.6771	15.6312	N	2.2412	-4.669	20.1469
C	-18.648	-0.5016	31.2485	C	-15.5153	-8.6464	15.8204	C	1.6263	-4.8304	18.9223
H	-19.2723	-2.5713	32.9464	C	-17.8278	-7.8029	16.0639	C	0.7947	-5.8509	18.6682
H	-19.9415	-4.8379	32.3923	N	-18.6658	-6.7447	15.8183	C	1.9245	-3.7756	17.9312
Zn	-17.8577	-1.6799	27.9133	C	-19.8064	-6.2732	16.446	N	2.7327	-2.7372	18.325
C	-12.7503	-4.9614	29.6531	C	-20.2066	-4.9814	16.0678	C	2.7639	-1.4044	17.9446
C	-12.8365	-6.2735	30.1252	C	-21.2954	-4.374	16.6945	C	3.4146	-0.5368	18.8375
C	-11.7254	-6.952	30.6396	C	-22.0064	-5.0701	17.6734	C	3.4349	0.8371	18.5925
C	-10.5132	-6.2488	30.6939	C	-21.626	-6.3634	18.0318	C	2.838	1.3422	17.4366

C	-10.38	-4.9367	30.2176	C	-20.5249	-6.9643	17.4227	C	2.2175	0.4798	16.5331
C	-11.5155	-4.3116	29.6894	N	-19.4499	-4.318	15.1056	C	2.1774	-0.8909	16.7863
H	-9.6361	-6.7295	31.1415	C	-19.8712	-3.0884	14.6201	N	3.9747	-1.0824	19.9884
H	-11.4503	-3.2926	29.2819	C	-21.0102	-2.4146	15.2846	C	4.7187	-0.2842	20.8456
H	-13.8209	-6.7581	30.1053	C	-21.6675	-3.0079	16.284	C	4.7353	1.1787	20.6178
C	-2.1854	-1.0755	23.0126	H	-12.7024	-7.0154	14.701	C	4.1065	1.7142	19.5688
C	-2.3997	-1.4805	24.3357	H	-14.4971	-5.2517	14.3377	H	0.6933	-7.2664	21.8025
C	-3.5643	-1.1257	25.0258	H	-15.8608	-9.5868	16.2732	H	2.3043	-5.3498	22.1679
C	-4.5495	-0.3895	24.3625	H	-18.2233	-8.706	16.5554	H	0.3692	-5.9323	17.6574
C	-4.3752	0.0197	23.0385	H	-22.8726	-4.5994	18.1664	H	1.5014	-3.7796	16.9142
C	-3.1775	-0.3049	22.3954	H	-22.1912	-6.9078	18.8067	H	2.8571	2.4253	17.2333
H	-5.4881	-0.1581	24.8892	H	-20.222	-7.979	17.7281	H	1.7484	0.8849	15.6208
H	-3.011	0.076	21.3782	C	-19.3164	-2.4365	13.3629	H	1.6694	-1.5583	16.0714
H	-1.64	-2.0803	24.8579	H	-21.3479	-1.4278	14.9256	C	5.6272	-0.8251	21.9392
C	-6.6621	-14.1768	25.2971	H	-22.5237	-2.5015	16.7592	H	5.3064	1.8325	21.2984
C	-6.0572	-13.0927	25.8039	C	-15.8733	-2.9699	18.792	H	4.1405	2.8034	19.4023
C	-4.5939	-12.9688	25.6838	C	-16.5885	-4.0753	19.0568	Zn	3.1953	-2.9096	20.2492
N	-3.8425	-13.9624	25.1003	C	-17.1975	-4.8271	17.9443	H	6.4848	-3.3739	16.9254
C	-4.4907	-15.0493	24.5477	N	-17.0702	-4.4017	16.6429	H	5.4132	-2.2163	17.7533
C	-5.8208	-15.1908	24.6222	C	-16.2781	-3.3005	16.3872	H	4.7149	-3.6379	16.8812
C	-3.6082	-16.0534	23.9178	C	-15.7171	-2.5841	17.3701	H	6.459	-0.1204	22.1598
N	-2.2539	-15.846	23.9991	C	-16.0941	-2.9765	14.9576	H	6.0983	-1.7854	21.6395
C	-1.1694	-16.2548	23.2421	N	-16.7216	-3.7777	14.0372	H	5.0675	-0.9792	22.8874

11 References

1. Castilla, A. M.; Ousaka, N.; Bilbeisi, R. A.; Valeri, E.; Ronson, T. K.; Nitschke, J. R. High-fidelity stereochemical memory in a $Fe^{II}{}_4L_4$ tetrahedral capsule. *J. Am. Chem. Soc.* **2013**, *135*, 17999−18006.

2. van der Waals radii in the book: Andrei, S. Batsanov. Small Molecule Applications of X-ray Diffraction. In Encyclopedia of Spectroscopy and Spectrometry, 2nd ed.; Lindon, J.C., Ed.; Academic Press: Oxford, UK, 2010; pp. 2550–2558.

3. Takezawa, H.; Murase, T.; Resnati, G.; Metrangolo, P.; Fujita, M. Recognition of polyfluorinated compounds through self-aggregation in a cavity. *J. Am. Chem. Soc.* **2014,** *136*, 1786-1788.

4. Allan, D.; Nowell, H.; Barnett, S.; Warren, M.; Wilcox, A.; Christensen, J.; Saunders, L.; Peach, A.; Hooper, M.; Zaja, L.; Patel, S.; Cahill, L.; Marshall, R.; Trimnell, S.; Foster, A.; Bates, T.; Lay, S.; Williams, M.; Hathaway, P.; Winter, G.; Gerstel, M.; Wooley, R. A novel dual air-bearing fixed-χ diffractometer for small-molecule single-crystal X-ray diffraction on beamline I19 at diamond light source. *Crystals* **2017**, *7*, 336.

5. (a) Winter, G. xia2: an expert system for macromolecular crystallography data reduction. *J. Appl. Crystallogr*. **2010**, *43*, 186−190; (b) Winter, G.; Waterman, D. G.; Parkhurst, J. M.; Brewster, A. S.; Gildea, R. J.; Gerstel, M.; Fuentes-Montero, L.; Vollmar, M.; Michels-Clark, T.; Young, I. D.; Sauter, N. K.; Evans, G. DIALS: implementation and evaluation of a new integration package. *Acta Crystallogr*. **2018**, *D74*, 85−97.

6. Farrugia, L., WinGX and ORTEP for Windows: an update. *J. Appl. Crystallogr*. **2012**, *45*, 849−854.

7. Sheldrick, G., SHELXT-integrated space-group and crystal-structure determination. *Acta. Crystallogr*. **2015**, *A71*, 3−8.

8. Sheldrick, G. M. Crystal structure refinement with SHELXL. *Acta. Crystallogr*. **2015**, *C71*, 3−8.

9. van der Sluis, P.; Spek, A. L. BYPASS: an effective method for the refinement of crystal structures containing disordered solvent regions. *Acta Crystallogr*. **1990**, *A46*,

194−201.

10. Spek, A. L. PLATON: A multipurpose crystallographic tool. Utrecht University: Utrecht, The Netherlands, 2008.

11. Stewart, J. J. P. Optimization of parameters for semiempirical methods VI: more modifications to the NDDO approximations and re-optimization of parameters. *J. Mol. Mod*. **2013**, *19*, 1–32.

12. Maglic, J. B.; Lavendomme, R. MoloVol: an easy-to-use program to calculate various volumes and surface areas of chemical structures and identify cavities. *J. Appl. Crystallogr*. **2022**, *55*, 1033–1044.

13. J. J. P. MOPAC2016; Stewart, Stewart Computational Chemistry: Colorado Springs, CO, USA, http://openmopac.net/MOPAC2016.html (accessed June 7, 2023).

14. (a) Fujitsu Ltd. *SCIGRESS*: Tokyo, Japan, 2013. (b) Stewart, J. J. P. Optimization of parameters for semiempirical methods V: modification of NDDO approximations and application to 70 elements. *J. Mol. Model.* **2007**, *13*, 1173−1213.
